# Synthesis of Highly Functionalizable Symmetrically and Unsymmetrically Substituted Triarylboranes from Bench‐Stable Boron Precursors

**DOI:** 10.1002/chem.202100632

**Published:** 2021-05-17

**Authors:** Matthias Ferger, Sarina M. Berger, Florian Rauch, Markus Schönitz, Jessica Rühe, Johannes Krebs, Alexandra Friedrich, Todd B. Marder

**Affiliations:** ^1^ Institut für Anorganische Chemie and Institute for Sustainable Chemistry & Catalysis with Boron Julius-Maximilians-Universität Würzburg Am Hubland 97074 Würzburg Germany

**Keywords:** boranes, borylation, chromophore, functionalization, synthetic methods

## Abstract

A novel and convenient methodology for the one‐pot synthesis of sterically congested triarylboranes by using bench‐stable aryltrifluoroborates as the boron source is reported. This procedure gives systematic access to symmetrically and unsymmetrically substituted triarylboranes of the types BAr_2_Ar’ and BArAr'Ar’’, respectively. Three unsymmetrically substituted triarylboranes as well as their iridium‐catalyzed C−H borylation products are reported. These borylated triarylboranes contain one to three positions that can subsequently be orthogonally functionalized in follow‐up reactions, such as Suzuki‐Miyaura cross‐couplings or Sonogashira couplings.

## Graphical abstract

**A widely applicable one‐pot synthetic approach** to air‐ and moisture‐stable triarylboranes with up to three different aromatic rings starting from bench‐stable boron precursors is reported. In addition, these compounds are shown to be easily post‐functionalized, thus making them versatile building blocks for the syntheses of functional materials.

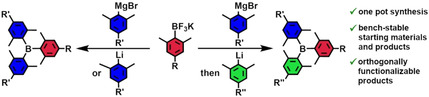

## Introduction

**Figure 1:**
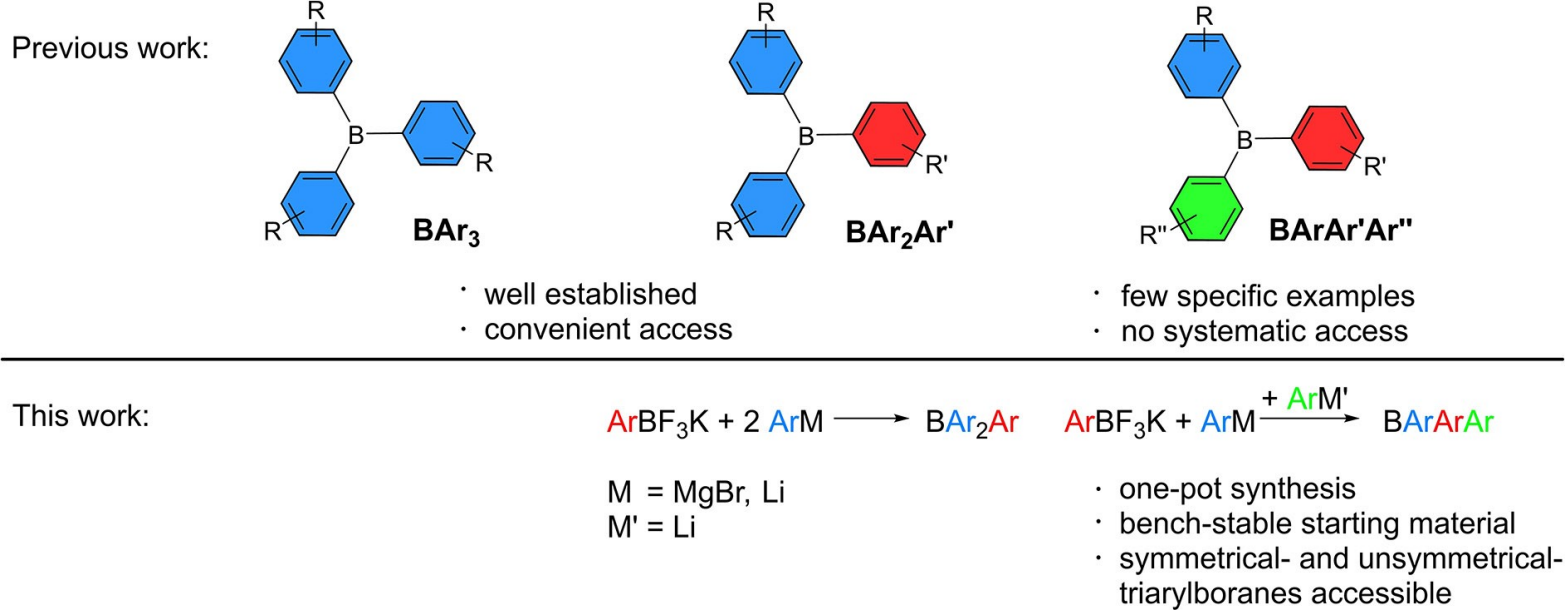
General types of triarylboranes^[16]^ and a schematic summary of this work.

Triarylboranes or compounds containing this structural motif can potentially find applications in various fields as functional materials.[^1^, ^2^, ^3^] The interesting properties of these functional materials originate from the vacant p_*z*_ orbital of the boron center that can efficiently conjugate with adjacent π‐systems. The electronic and optical, physical, biological, and chemical properties of such systems can be tuned by variation of the substituents on the three aryl rings attached to the boron atom.[^4^, ^5^, ^6^, ^7^, ^8^, ^9^] In general, there are three types of triarylboranes (Figure 1). The first type bears three identical substituents on the boron center (BAr_3_) resulting in a high symmetry (*D*_3_) and, due to its simplicity, this type of triarylborane was the first to be reported in 1885.^[10]^ A reliable methodology for the synthesis of these compounds was reported by Krause and coworkers in 1922 wherein aryl Grignard reagents were reacted with gaseous boron trifluoride to form the corresponding triarylborane.^[11]^ By changing the boron source to the more convenient boron trifluoride etherate and using aryl metalates of different reactivity,[^12^, ^13^, ^14^] this strategy became widely applicable. Less symmetrical triarylboranes of the type BAr_2_Ar’ (*C*_2_) have been systematically accessible since 1972.^[15]^ In this procedure, sterically demanding aryl metalates, for example, Grignard reagents, are reacted with BX_3_ species to form the corresponding XBAr_2_ derivative. Due to the steric congestion, the reaction stops after the attachment of two aryl moieties to the boron center. Then, a second, more reactive aryl metalate, for example, an organolithium reagent, is used to attach the third aryl ring to the boron center. This is still the most common way to synthesize *C*_2_ symmetrically substituted triarylboranes.^[16]^ The third class of triarylborane bears three different aryl substituents (BArAr'Ar’’) and will, in the following, be referred to as unsymmetrically substituted triarylboranes. Only a few examples of unsymmetrically substituted BArAr'Ar’’ triarylboranes have been reported in the literature, and no general synthetic methodology is known. We have recently published a detailed review on the historical development of synthetic strategies for the preparation of triarylboranes.^[16]^ The unsymmetrically substituted triarylboranes reported to date have been synthesized *via* extensive, multistep procedures,[^17^, ^18^] from symmetrically substituted precursors,[^19^, ^20^, ^21^] or with sterically less demanding substituents attached to the boron center,[^22^, ^23^, ^24^, ^25^] mainly starting from highly reactive, and therefore not bench‐stable boron halides. In contrast, the work presented herein provides a systematic, one‐pot approach to BArAr'Ar’’ triarylboranes starting from bench‐stable potassium aryltrifluoroborates as the boron source.

**Figure 2:**
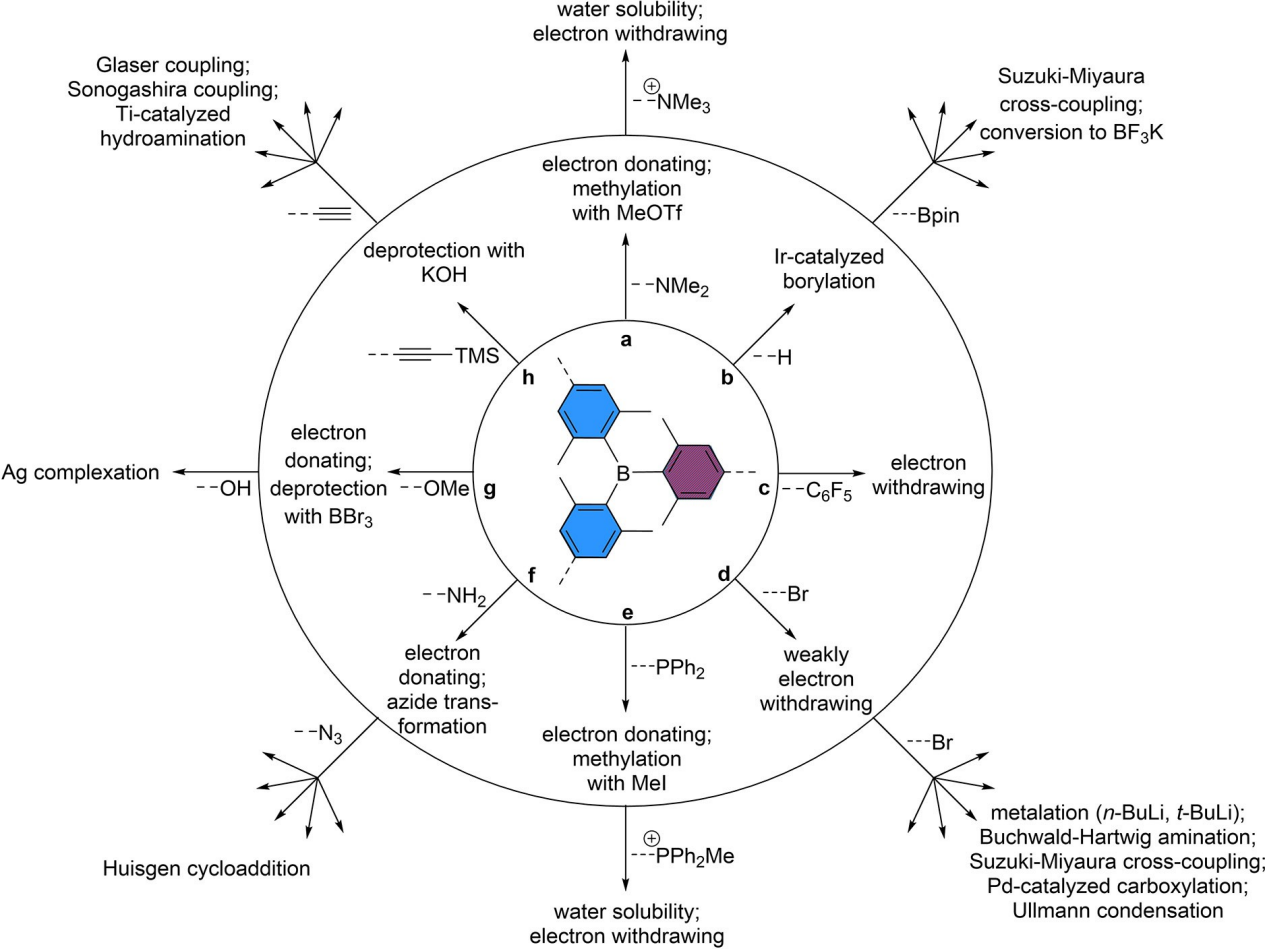
Overview of reported follow‐up reactions, to which the BXyl_3_ moiety (BAr_3_ or BAr_2_Ar’) is tolerant, and selected functional groups that have been attached. Examples of each path are given in the following references: a,[^26^, ^27^, ^28^, ^29^, ^30^] b,[^27^, ^29^, ^30^, ^31^, ^32^, ^33^] c,[^34^, ^35^] d,[^36^, ^37^, ^38^, ^39^, ^40^, ^41^, ^42^, ^43^, ^44^, ^45^, ^46^, ^47^, ^48^, ^49^, ^50^, ^51^] e,[^52^, ^53^, ^54^, ^55^, ^56^, ^57^] f,[^42^, ^58^, ^59^, ^60^, ^61^, ^62^] g,[^63^, ^64^, ^65^, ^66^, ^67^] and h.[^68^, ^69^, ^70^, ^71^, ^72^, ^73^, ^74^, ^75^]

The optional functional groups to incorporate, and the possibilities to do follow‐up reactions on triarylboranes, have increased considerably over the past years. The applicability of triarylboranes in many fields is limited by their instability towards hydrolysis. One of the easiest and most common ways to stabilize triarylboranes kinetically is to introduce *ortho*‐methyl groups around the boron center. It was demonstrated that compounds bearing six *ortho*‐methyl groups around the boron are stable in pure water for several days.[^26^, ^27^] Due to the kinetic stabilization of the boron center, the BXyl_3_ (Xyl=2,6‐MeC_6_H_3_) moiety exhibits a high tolerance to different reaction conditions employed in functionalization reactions. An overview of the numerous reported reaction conditions and selected functional groups attached to the BXyl_3_ moiety is given in Figure 2.

The most notable effect on the properties of triarylboranes is obtained by functionalization of the *para*‐position on the 2,6‐dimethylphenyl substituents. This can be achieved, for example, by iridium‐catalyzed C−H borylation which exhibits very high, sterically driven regioselectivity.[^76^, ^77^] The resulting boronate ester moieties can be employed in various functional group transformations as well as Suzuki‐Miyaura cross‐coupling reactions (Figure 2b).[^27^, ^29^, ^30^, ^31^, ^32^, ^33^] In this way, triarylboranes can also be attached to chromophores or stationary phases. Even deprotonation or lithium‐halide exchange reactions can be employed to functionalize triarylboranes due to their high tolerance towards different reaction conditions (Figure 2d).[^36^, ^37^, ^38^, ^39^, ^40^, ^41^, ^42^, ^43^, ^44^, ^45^, ^46^, ^47^, ^48^, ^49^, ^50^, ^51^] By reaction with different electrophiles, various functional groups, for example, CO_2_H or COH, can be introduced. In general, introduction of electron donating or accepting groups has a strong effect on the optoelectronic properties of triarylboranes.[^29^, ^35^] By carefully choosing the specific substituents, the properties can be fine‐tuned to meet different requirements. For example, water solubility of triarylboranes can be facilitated by the introduction of cationic ammonium or phosphonium substituents (Figure 2e and f).[^42^, ^52^, ^53^, ^54^, ^55^, ^56^, ^57^, ^58^, ^59^, ^60^, ^61^, ^62^]

Due to the limited synthetic access to unsymmetrically substituted triarylboranes, structure‐property relationship studies have been mostly limited to symmetrically substituted triarylboranes (BAr_3_ or BAr_2_Ar’). Consequently, even though a plethora of functional groups is available, a maximum of two can be realized at once. Thus, a systematic synthetic methodology to access unsymmetrically substituted triarylboranes would greatly enhance the options available for designing functional materials.

The main boron sources for triarylborane syntheses are boron trihalides, mostly employed in the form of boron trifluoride etherate.^[16]^ In recent years, we and others have employed aryltrifluoroborates as convenient, bench‐stable precursors for the synthesis of triarylboranes.[^78^, ^79^, ^80^] Previously, aryltrifluoroborates were found to be efficient reagents for cross‐coupling reactions.[^81^, ^82^] Aryltrifluroborate salts are easily accessible through fluorination of the corresponding boronate ester or boronic acid by reaction with KHF_2_.^[83]^

Based on these results, we present a synthetic route to symmetrically and unsymmetrically substituted, sterically congested and, therefore, air‐stable triarylboranes starting from bench‐stable boron precursors, namely potassium aryltrifluoroborates.

## Results and Discussion

### Synthesis of symmetrically substituted triarylboranes

**Scheme 1:**
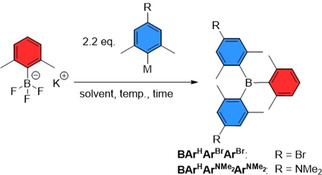
Synthesis of the C_2_ symmetric triarylboranes BAr^H^Ar^Br^Ar^Br^ and BAr^H^Ar${}^{\mathrm{NMe}{}_{2}}$ Ar${}^{\mathrm{NMe}{}_{2}}$ .

To optimize the formation of *para*‐substituted BXyl_3_ from potassium aryltrifluoroborates, our first approach was to examine the synthesis of the compound BAr^H^Ar^Br^Ar^Br^ (Ar refers to 2,6‐dimethylphenl, and the superscripts refer to the substituent *para* to the boron center) under different reaction conditions (Scheme 1, Table 1).

**Table 1:** Summary of conditions applied for the synthesis of the symmetrically substituted triarylboranes BAr^H^Ar^Br^Ar^Br^ and BAr^H^Ar${}^{\mathrm{NMe}{}_{2}}$ Ar${}^{\mathrm{NMe}{}_{2}}$ .

	BAr_2_Ar’	M	R	Solvent	*T* [°C]	*t*	Yield [%]^[c]^
1	BAr^H^Ar^Br^Ar^Br^	MgBr	Br	THF	90	2 d	19
2		MgBr	Br	THF	200^[d]^	1 h	19
3		Li	Br	THF	RT	3 d	23
4^[a]^		Li	Br	Et_2_O	RT	2 d	43
5^[b]^		Li	Br	Et_2_O	RT	3 d	51
6	BAr^H^Ar${}^{\mathrm{NMe}{}_{2}}$ Ar${}^{\mathrm{NMe}{}_{2}}$	Li	NMe_2_	THF	RT	18 h–2 d	63

[a] Activation of Ar^H^BF_3_K performed in Et_2_O; [b] Activation of Ar^H^BF_3_K performed in THF; [c] Isolated yield; [d] Microwave irradiation in a sealed tube.

Therefore, 2.2 equivalents of (4‐bromo‐2,6‐dimethylphenyl)metalate were treated with potassium (2,6‐dimethylphenyl)trifluoroborate Ar^H^BF_3_K. Using a Grignard reagent in THF as the metalate, BAr^H^Ar^Br^Ar^Br^ was isolated in 19 % yield after stirring for 2 days at 90 °C in a sealed vessel (Table 1). The reaction time can be significantly reduced to 1 h when using microwave irradiation at 200 °C. Exchanging the Grignard reagent for the more reactive corresponding aryl lithium reagent, BAr^H^Ar^Br^Ar^Br^ was isolated in a slightly higher yield (23 %) after stirring for 3 days at room temperature. It is well documented that by reaction of trimethylsilyl chloride (TMSCl) and aryltrifluoroborates, the reactivity of the latter is increased due to partial formation of difluoroboranes.^[83]^ Therefore, it was possible to increase the isolated yield of BAr^Br^Ar^Br^Ar^H^ to 43 % when activating Ar^H^BF_3_K with TMSCl in diethyl ether prior to reaction with the aryl lithium reagent. This yield was further increased to 51 % by changing the solvent for the activation to THF, as the solubility of 4‐bromo‐2,6‐dimethylphenyl lithium is low in diethyl ether.

To demonstrate further the utility of our reaction, the reaction conditions were applied in the synthesis of BAr^H^Ar${}^{\mathrm{NMe}{}_{2}}$ Ar${}^{\mathrm{NMe}{}_{2}}$ . This compound is frequently used in our work as a starting material for the synthesis of water‐soluble chromophores for biological applications.[^27^, ^29^, ^30^, ^74^, ^75^] BAr^H^Ar${}^{\mathrm{NMe}{}_{2}}$ Ar${}^{\mathrm{NMe}{}_{2}}$ was previously only accessible in a yield of 33 % over two steps, as we reported in 2016.[^27^, ^84^] Using our new approach, 4‐(*N*,*N*‐dimethylamino)‐2,6‐dimethylphenyl lithium was reacted with Ar^H^BF_3_K according to Scheme 1, to give BAr^H^Ar${}^{\mathrm{NMe}{}_{2}}$ Ar${}^{\mathrm{NMe}{}_{2}}$ in a good yield of up to 63 % in one step. This reaction shows that the activation of the potassium aryltrifluoroborates is not required for highly reactive lithiated species, such as 4‐(*N*,*N*‐dimethylamino)‐2,6‐dimethylphenyl lithium.

From these reactions, it can be concluded that sterically very congested triarylboranes can be synthesized from Ar^H^BF_3_K and two equivalents of aryl Grignard reagent at elevated temperatures. In contrast, at room temperature, the reaction of Ar^H^BF_3_K with two equivalents of an aryl Grignard reagent stops after the attachment of one additional aryl ring to the boron center, for reaction times of up to five days. This leads to a diarylfluoroborane (BFArAr’) with four *ortho*‐methyl groups. To synthesize symmetrically substituted triarylboranes (BAr_2_Ar’) from aryltrifluoroborates at room temperature without a loss in yield, more reactive lithiated arenes must be used.

### Synthesis of unsymmetrically substituted triarylboranes

**Scheme 2:**
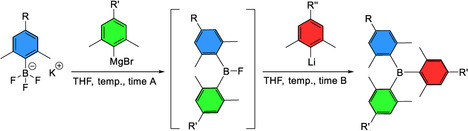
Summary of synthesis of the unsymmetrically substituted triarylboranes BAr^H^Ar^Br^Ar^Me^, BAr^H^Ar^Br^Ar${}^{\mathrm{SiMe}{}_{3}}$ , and BAr^H^Ar^Me^Ar${}^{\mathrm{NMe}{}_{2}}$ .

As aryl Grignard and aryl lithium reagents differ significantly in their reactivities towards potassium aryltrifluoroborates, this approach was used for the synthesis of unsymmetrically substituted triarylboranes according to Scheme 2 and Table 2.

**Table 2:** Summary of conditions applied for the synthesis of the unsymmetrically substituted triarylboranes BAr^H^Ar^Br^Ar^Me^, BAr^H^Ar^Br^Ar${}^{\mathrm{SiMe}{}_{3}}$ , and BAr^H^Ar^Me^Ar${}^{\mathrm{NMe}{}_{2}}$ .

	BArAr'Ar’’	R	R’	R’’	*T*	*t* A	*t* B	Yield [%]^[b]^
1	BAr^H^Ar^Br^Ar^Me^	H	Br	Me	RT	18 h	3 d	18
2		H	Br	Me	RT	30 min	18 h	18
3		Me	Br	H	RT	30 min	18 h	35
4		Br	H	Me	RT	30 min	18 h	3
5	BAr^H^Ar^Br^Ar${}^{\mathrm{SiMe}{}_{3}}$	H	Br	SiMe_3_	RT	30 min	18 h	16
6	BAr^H^Ar^Me^Ar${}^{\mathrm{NMe}{}_{2}}$	H	Me	NMe_2_	RT	18 h	3 d	trace
7		H	Me	NMe_2_	70 °C	18 h	2 d	5
8		H	NMe_2_	Me	RT	18 h	3 d	10
9^[a]^		H	NMe_2_	Me	RT	18 h	18 h–3 d	51

[a] Intermediate diaryl fluoroborane was isolated. [b] Isolated yield.

**Scheme 3:**
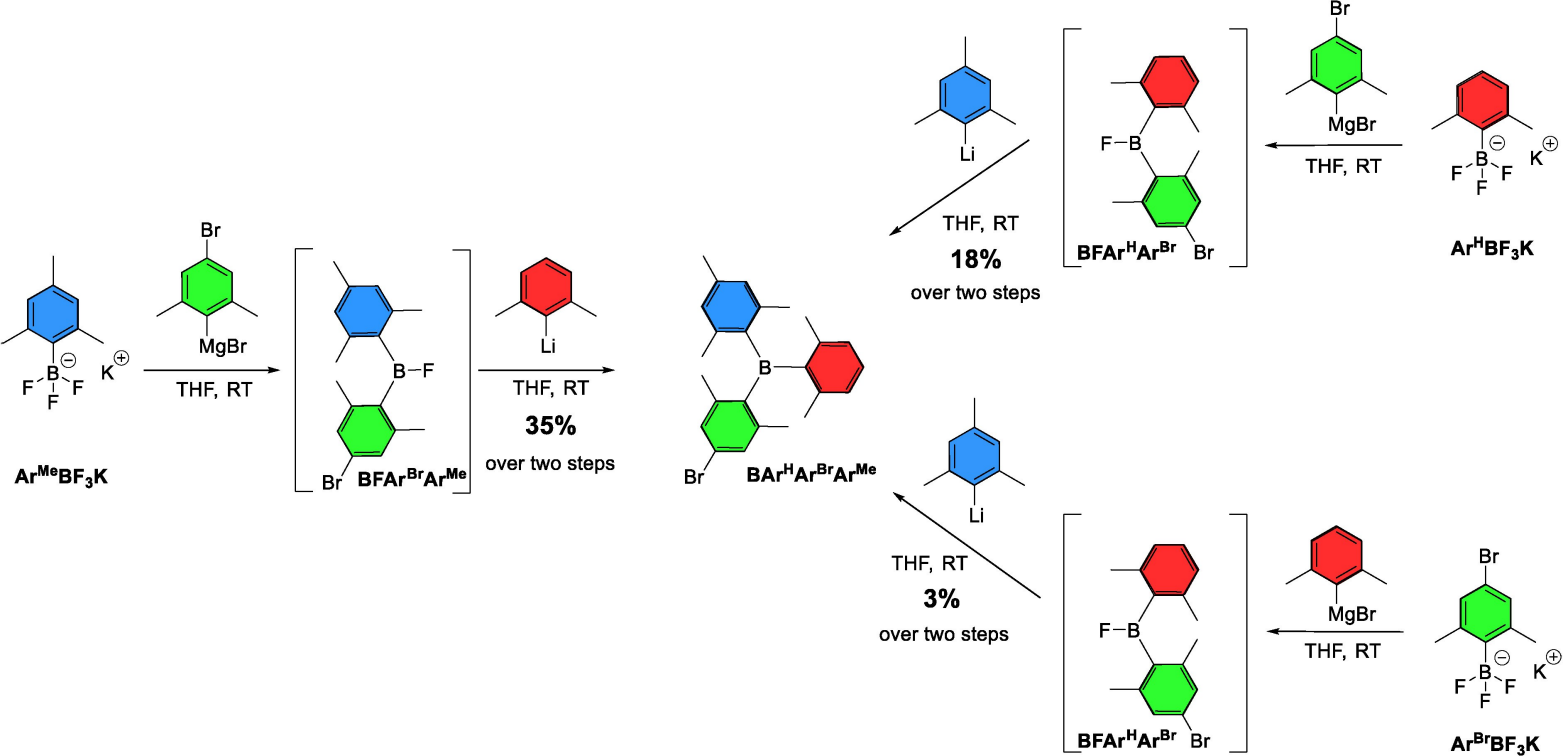
Different reaction sequences to prepare BAr^H^Ar^Br^Ar^Me^.

To this end, Ar^H^BF_3_K was reacted with one equivalent of (4‐bromo‐2,6‐dimethylphenyl)magnesium bromide at room temperature, to form the corresponding fluoroborane (BFArAr’), as indicated by in situ ^19^F and ^11^B NMR spectroscopy. Then, the more reactive 2,4,6‐trimethylphenyl lithium (MesLi) was added and the unsymmetrically substituted triarylborane BAr^H^Ar^Br^Ar^Me^ was isolated in 18 % yield. The formation of BFArAr’ is rather fast, and the same yields were obtained for shorter reaction times (Table 2 entry 1 vs. entry 2). Thus, the synthesis of BAr^H^Ar^Br^Ar^Me^ is possible in a one pot process within 24 h. To optimize the conditions further, the influence of the *para* substituents on the potassium aryltrifluoroborate was examined. The synthesis of BAr^H^Ar^Br^Ar^Me^ was carried out starting from the respective aryltrifluoroborate Ar^H^BF_3_K, potassium mesityltrifluoroborate Ar^Me^BF_3_K and potassium 4‐bromo‐(2,6‐dimethylphenyl)trifluoroborate Ar^Br^BF_3_K. Our reaction procedure was performed starting from each potassium aryltrifluoroborate according to Scheme 3 and Table 2, entries 2–4.

For each reaction, the desired product BAr^H^Ar^Br^Ar^Me^ was isolated. Using Ar^H^BF_3_K as the starting material, we obtained a yield of 18 % over two steps (see above). Starting from Ar^Me^BF_3_K, the yield doubled to 35 % over two steps. With Ar^Br^BF_3_K as the starting material, the yield over two steps decreased to 3 %. These drastic differences in yield were surprising, as the electronic differences of the potassium aryltrifluoroborates were considered minor in comparison to the equal steric demand at the boron centers of all aryltrifluoroborates. The electronic differences for substituents at the *para* position can be assessed by the Hammett values and are *σ*_p_=−0.17 for CH_3_, 0 for H and 0.23 for bromide.^[85]^ The trend found in the isolated yields of BAr^H^Ar^Br^Ar^Me^ are in good accordance with the trend given by the *para* Hammett values of the respective substituents in the potassium aryltrifluoroborates Ar^Me^BF_3_K, Ar^H^BF_3_K and Ar^Br^BF_3_K. It is well documented that electron donating groups (EDGs) enhance solvolysis of aryltrifluoroborates, whereas electron withdrawing groups (EWGs) hinder solvolysis.[^86^, ^87^] In analogy to their benzotrifluoride analogues,[^88^, ^89^] this is explained by the proclivity of aryltrifluoroborate to lose a fluorine atom in the first step of the solvolysis. The first step of the solvolysis is described as an equilibrium between the aryltrifluoroborate and the short‐lived aryldifluoroborane. This intermediate is stabilized by electron donating groups, whereas electron withdrawing groups stabilize the negative charge at the boron in the case of aryltrifluoroborates. Even though the mechanism of our triarylborane synthesis is unknown, both a dissociative as well as an associative mechanism would energetically benefit from electron donating groups at the *para* position of the aryltrifluoroborate salts, as B−F bond cleavage seems to be the critical factor. Thus, while our method provides considerable flexibility, to enhance the yield, electron‐rich potassium aryltrifluoroborates should be chosen as the boron source, if possible.

The unsymmetrically substituted triarylborane BAr^H^Ar^Br^Ar^Me^ allows further orthogonal functionalization at two positions, namely the *para* bromide and the *para* proton (Figure 2). The mesitylene unit does not possess a position that can be conveniently functionalized. To obtain a triarylborane that can be orthogonally functionalized at all three aromatic rings, the *para* methyl group of mesitylene was exchanged for a trimethylsilyl group. This moiety permits many different functionalization reactions[^90^, ^91^, ^92^, ^93^, ^94^] and should be robust enough to tolerate the reaction conditions required for the triarylborane formation. We chose Ar^H^BF_3_K as the starting material and synthesized compound BAr^H^Ar^Br^Ar${}^{\mathrm{SiMe}{}_{3}}$ in a yield of 16 % over two steps in a one pot synthesis (Scheme 2, Table 2 entry 5).

To test whether our methodology can be applied for the synthesis of a compound containing an electron donating group, triarylborane BAr^H^Ar^Me^Ar${}^{\mathrm{NMe}{}_{2}}$ was chosen as a model system in analogy to BAr^H^Ar${}^{\mathrm{NMe}{}_{2}}$ Ar${}^{\mathrm{NMe}{}_{2}}$ . Therefore, in the first step, Ar^H^BF_3_K was reacted with a slight excess of mesitylmagnesium bromide. The conversion of the aryl BF_3_K salt to the diarylfluoroborane BFAr^H^Ar^Me^ was monitored by in situ ^19^F and ^11^B NMR spectroscopy. Then, a THF solution of 4‐(*N*,*N*‐dimethylamino)‐2,6‐dimethylphenyl lithium was added. Stirring at room temperature for 3 days resulted only in trace amounts of BAr^H^Ar^Me^Ar${}^{\mathrm{NMe}{}_{2}}$ , detected *via* high‐resolution mass‐spectrometry (HRMS). Therefore, the reaction temperature in the second step was increased to 70 °C to give BAr^H^Ar^Me^Ar${}^{\mathrm{NMe}{}_{2}}$ in a 5 % isolated yield (Scheme 2, Table 2 entry 7). This was further improved as the choice of Grignard and aryl lithium reagent was changed. When the 4‐*N*,*N*‐dimethylamino‐2,6‐dimethylphenyl motif was introduced as the Grignard reagent and the mesityl moiety was added as the lithium reagent, the reaction sequence gave the triarylborane BAr^H^Ar^Me^Ar${}^{\mathrm{NMe}{}_{2}}$ in 10 % isolated yield in a one pot approach at room temperature (Scheme 2, Table 2 entry 8). This demonstrates that the choice of aryl Grignard and aryl lithium reagents is crucial to improve the isolated yields.

Given the fact that using more electron‐rich potassium aryltrifluoroborates as starting materials results in higher yields (see above), the synthesis of BAr^H^Ar^Me^Ar${}^{\mathrm{NMe}{}_{2}}$ from potassium (*N*,*N*‐dimethylamino‐2,6‐dimethylphenyl)trifluoroborate Ar${}^{\mathrm{NMe}{}_{2}}$ BF_3_K should give higher yields. However, it was not possible to synthesize Ar${}^{\mathrm{NMe}{}_{2}}$ BF_3_K by our methodology as proteodeborylation during fluorination of the intermediate ArB(OMe)_2_ occurs. This indicates that not all potassium aryltrifluoroborates are readily available.

However, to increase the isolated yield, the isolation of the proposed intermediate fluoroborane BFAr^H^Ar${}^{\mathrm{NMe}{}_{2}}$ from a reaction mixture of Ar^H^BF_3_K and (4‐(*N*,*N*‐dimethylamino)‐2,6‐dimethylphenyl)magnesium bromide was attempted. It was possible to isolate BFAr^H^Ar${}^{\mathrm{NMe}{}_{2}}$ in ca. 89 % yield, still containing residual Grignard reagent. At room temperature, the remaining Grignard reagent will not form triarylboranes (see above). Therefore, without further purification, the fluoroborane BFAr^H^Ar${}^{\mathrm{NMe}{}_{2}}$ was added to a solution of mesityl lithium. After stirring at room temperature for 3 days, BAr^H^Ar^Me^Ar${}^{\mathrm{NMe}{}_{2}}$ was isolated in up to 51 % yield. Thus, the isolated yield can be increased from 10 % in the one pot approach to 51 % by one additional step, in which purely inorganic metal salts are separated from BFAr^H^Ar${}^{\mathrm{NMe}{}_{2}}$ . Further experiments showed that the reaction time of the second step can be decreased from 3 days to 18 h without any loss in yield.

Interestingly, activation of Ar^H^BF_3_K prior to reaction with the Grignard reagent did not improve the yield of BFAr^H^Ar${}^{\mathrm{NMe}{}_{2}}$ . This might be attributed to the increased reactivity of (4‐(*N*,*N*‐dimethylamino)‐2,6‐dimethylphenyl)magnesium bromide compared to Grignard reagents without strong electron donating groups, as similar behavior was observed for the synthesis of BAr^H^Ar${}^{\mathrm{NMe}{}_{2}}$ Ar${}^{\mathrm{NMe}{}_{2}}$ (see above). In turn, increasing the reactivity of Ar^H^BF_3_K by activation with trimethylsilyl chloride does not affect the isolated yield of BAr^H^Ar^Me^Ar${}^{\mathrm{NMe}{}_{2}}$ .

However, it is apparent that the yield depends not only on the choice of potassium aryltrifluoroborate, but also on the choice of Grignard and aryl lithium reagent (Table 2). In addition, all triarylboranes synthesized bear at least one position that can be functionalized after the formation of the triarylborane, making these compounds versatile building blocks for the synthesis of larger, boron‐containing compounds.

### Selected examples of post‐functionalization

**Scheme 4:**
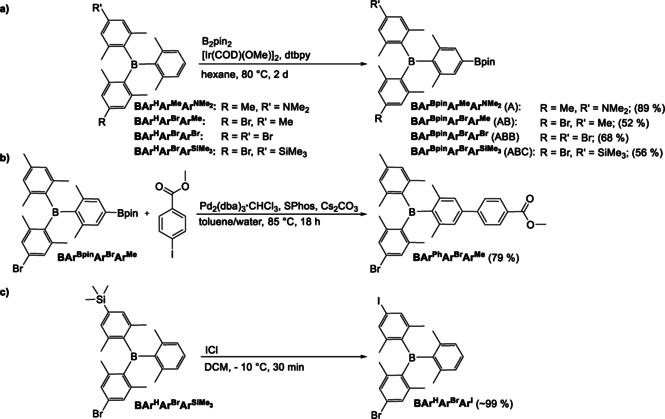
a) Synthesis of highly functionalizable triarylboranes BAr^Bpin^Ar^Me^Ar${}^{\mathrm{NMe}{}_{2}}$ , BAr^Bpin^Ar^Br^Ar^Me^, BAr^Bpin^Ar^Br^Ar^Br^ and BAr^Bpin^Ar^Br^Ar${}^{\mathrm{SiMe}{}_{3}}$ obtained by iridium‐catalyzed C−H borylation. The letters A, B and C in brackets indicate possible orthogonal post‐functionalization reactions. b) Selective Suzuki‐Miyaura cross‐coupling of BAr^Bpin^Ar^Br^Ar^Me^ with iodo methylbenzoate. c) Conversion of the trimethylsilyl group of BAr^H^Ar^Br^Ar${}^{\mathrm{SiMe}{}_{3}}$ to iodide with iodine monochloride.

Iridium‐catalyzed C−H borylation using [Ir(COD)OMe]_2_ as the precatalyst, 4,4’‐di‐*tert‐*butylbipyridine as the ligand and B_2_pin_2_ as the boron source[^76^, ^77^] exhibits very high sterically driven selectivity and has been applied in our group for the synthesis of many different functional materials,[^80^, ^95^, ^96^, ^97^, ^98^] including triarylboranes.[^27^, ^29^, ^30^] To demonstrate the utility of our novel compounds, we applied iridium‐catalyzed C−H borylation to BAr^H^Ar^Me^Ar${}^{\mathrm{NMe}{}_{2}}$ , BAr^H^Ar^Br^Ar^Me^, BAr^H^Ar^Br^Ar^Br^ and BAr^H^Ar^Br^Ar${}^{\mathrm{SiMe}{}_{3}}$ . This borylation takes place selectively at the *para* C−H bond of the 2,6‐dimethylphenyl ring, thus yielding triarylboranes BAr^Bpin^Ar^Me^Ar${}^{\mathrm{NMe}{}_{2}}$ , BAr^Bpin^Ar^Br^Ar^Me^, BAr^Bpin^Ar^Br^Ar^Br^ and BAr^Bpin^Ar^Br^Ar${}^{\mathrm{SiMe}{}_{3}}$ . The products shown in Scheme 4a were synthesized in up to 89 % yield. These compounds represent a small library of A, AB, ABB, and ABC type, orthogonally functionalizable triarylboranes, wherein each letter indicates a different, orthogonally functionalizable group, in this case Bpin, bromine or trimethyl silyl.

As shown in Figure 2, borylated triarylboranes have already been employed successfully in Suzuki‐Miyaura cross‐coupling reactions with different aryl halides. However, the bromide, which is present in three of our four borylated triarylboranes, might lead to unwanted side reactions. To see if this can be circumvented by choice of an appropriate, more reactive aryl halide, we employed BAr^Bpin^Ar^Br^Ar^Me^ in a Suzuki‐Miyaura cross‐coupling reaction with methyl iodobenzoate (Scheme 4b). The coupling product BAr^Ph^Ar^Br^Ar^Me^ was isolated in a good yield of 79 % and the presence of the bromide in BAr^Ph^Ar^Br^Ar^Me^ was confirmed.

As mentioned above, the trimethylsilyl group permits many functionalization reactions. However, this functional group has not been used for functionalizations when attached to a BXyl_3_ moiety. To demonstrate the applicability of this functional group in our system, we converted the trimethylsilyl group of BAr^H^Ar^Br^Ar${}^{\mathrm{SiMe}{}_{3}}$ with iodine monochloride into iodide (Scheme 4c). The reaction was performed open to air and was complete within half an hour. The compound BAr^H^Ar^Br^Ar^I^ was isolated in quantitative yield.

## Conclusion

In summary, we have provided herein a generally applicable synthetic route to highly functionalizable, kinetically stable, symmetrically and unsymmetrically substituted triarylboranes. We show that it is possible to synthesize the same unsymmetrically substituted triarylborane starting from the three possible aryl BF_3_K salts in one‐pot syntheses, but the most electron‐rich aryl BF_3_K should be chosen, if possible. In a different example, it was shown that the choice of aryl lithium and aryl Grignard reagent also influences the yield of the triarylboranes. Furthermore, we have demonstrated that the isolated yield of the triarylborane can be improved with one additional step in which purely inorganic metal salts are separated from the intermediately formed diaryl fluoroborane prior to reaction with the aryl lithium reagent. To illustrate the utility of our method, the triarylboranes synthesized were functionalized by iridium‐catalyzed C−H borylation leading to versatile A‐, AB‐, ABB‐ and ABC‐type systems that can be further functionalized orthogonally and, thus, be used as building blocks in the design of boron‐containing functional materials. Further, the trimethylsilyl group was found to be conveniently convertible into a halide in triarylborane chemistry.

## Conflict of interest

The authors declare no conflict of interest.

## Supporting information

As a service to our authors and readers, this journal provides supporting information supplied by the authors. Such materials are peer reviewed and may be re‐organized for online delivery, but are not copy‐edited or typeset. Technical support issues arising from supporting information (other than missing files) should be addressed to the authors.

Supplementary
