# Supplementary material for: Synthesis of Highly Functionalizable Symmetrically and Unsymmetrically Substituted Triarylboranes from Bench‐Stable Boron Precursors
Source: Chemistry. 2021 May 17;27(35):9094–101. doi: 10.1002/chem.202100632 (PMC8360097; doi:10.1002/chem.202100632)
Supplement: Supplementary file 1 — Supplementary [file CHEM-27-9094-s001.pdf]

Chemistry–A European Journal

Supporting Information

Synthesis of Highly Functionalizable Symmetrically and Unsymmetrically Substituted Triarylboranes from Bench-Stable Boron Precursors

Matthias Ferger[+], Sarina M. Berger[+], Florian Rauch, Markus Schönitz, Jessica Rühe, Johannes Krebs, Alexandra Friedrich, and Todd B. Marder*

Table of Contents

Experimental Section…………………………………………………………………………...........S2

Single-crystal X-ray Diffraction Data...S20

NMR Spectra...S23

References……………………………………………………………………………………….S47

Experimental Section

General Information

Unless otherwise noted, the following conditions apply: each reaction was performed using standard Schlenk or glovebox (Innovative Technology Inc.) techniques under argon. Only oven-dried and additionally flame-dried glassware was used. The work up procedure was performed open to the air. Solvents used for reactions (THF, CH_2Cl_2, hexane, and Et_2O) were dried, deoxygenated and argon saturated using an Innovative Technology Inc. Pure Solvent Purification System. Deuterated solvents (CD_2Cl_2, C_6D_6, acetone-d_6) used for nuclear magnetic resonance spectroscopy were purchased from Sigma Aldrich. Trimethylsilyl chloride was distilled and stored under argon prior to use. *n*-Butyllithium (2.5 M solution in hexane), *t*-butyllithium (1.7 M solution in pentane) and KHF_2 were purchased from Sigma Aldrich and used as received, and 2-iodo-5-bromo-1,3-dimethylbenzene was purchased from Apollo Scientific Limited and passed through a silica plug with hexane prior to use. *N*,*N*,3,5-Tetramethylaniline was purchased from TCI or Sigma Aldrich and used as received. B_2pin_2 was kindly provided by AllylChem Co. Ltd. (Dalian, China). $[Ir(COD)(\mu\text{-}OMe)]_2$,[1] 4-bromo-*N*,*N*,3,5-tetramethylaniline,[2] and 4-(*N*,*N*-dimethylamino)-2,6-dimethylphenyllithum,[3] $Pd_2(dba)_3 \cdot CHCl_3$[4] were synthesized according to literature procedures.

Reaction progress was monitored using a GC-MS system (Agilent 7890A gas chromatograph (column: HP-5MS 5% phenyl methyl siloxane, 10 m, 0.25 mm, film 0.25 µm; injector: 250 °C; oven: 40 °C to 180 °C (20 °C / min), 180 °C to 280 °C (50 °C / min); carrier gas: He (1.2 mL / min) equipped with an Agilent 5975C inert MSD detector operating in EI mode and an Agilent 7693A series liquid handling system functioning as auto sampler) or by thin layer chromatography (TLC) using plates pre-coated with a layer of either silica (Polygram® Sil G/UV254) with fluorescent indicator UV254 or aluminum oxide, purchased from Marchery-Nagel. Automated flash chromatography was performed using a Biotage® Isolera Four system on silica gel (Biotage SNAP cartridges HP-Sil and KP-Sil with the cartridge size depending on substance mass according to the Biotage handbook), obtained from Biotage. Solvents were generally removed using a rotary evaporator *in vacuo* at a maximum temperature of 50 °C. Column chromatography was performed using silica gel 60 (0.040 – 0.063 mm) or aluminum oxide 90 (basic, activity I) purchased from Macherey-Nagel as the stationary phase.

^{1}H, $^{13}C\{^{1}H\}$, $^{11}B\{^{1}H\}$ NMR spectra were obtained, unless otherwise stated, at ambient temperature using a Bruker Avance 300 III (operating at 300 MHz for ^{1}H, 75 MHz for $^{13}C\{^{1}H\}$ and 96 MHz for $^{11}B\{^{1}H\}$), or a Bruker Avance 500 NMR spectrometer (operating at 500 MHz for ^{1}H, 125 MHz for $^{13}C\{^{1}H\}$, 160 MHz for $^{11}B\{^{1}H\}$, and 470.6 MHz for ^{19}F). Chemical shifts (δ)

were referenced to solvent peaks as follows. ^{1}H NMR spectra were referenced via residual proton resonances of CD_2Cl_2 (5.32 ppm), C_6D_6 (7.16 ppm) and acetone-d_6 (2.05 ppm). ^{13}C NMR spectra were referenced to CD_2Cl_2 (53.84 ppm) C_6D_6 (128.06 ppm) and acetone-d_6 (206.06 ppm and 29.84 ppm). ^{11}B NMR signals are quoted relative to external $BF_3{\cdot}OEt_2$.

Elemental analyses were performed on an Elementar vario MICRO cube elemental analyzer. High-resolution mass spectrometry was performed with a High-resolution Thermo Fisher Scientific Exactive Plus Orbitrap MS System. ESI measurements were performed with a HESI source at 50 °C. APCI and ASAP measurements were performed with an ACPI source and Corona needle at 400 °C, unless otherwise noted. LIFDI measurements were performed with a Linden CMS *LIFDI 700* unit.

The X-ray crystallographic data for **$BAr^{H}Ar^{Br}Ar^{SiMe_3}$** were collected on a BRUKER X8-APEX II diffractometer with a CCD area detector and graphite monochromated Mo-K_α radiation. The data for **$BAr^{H}Ar^{Br}Ar^{Br}$**, **$BAr^{H}Ar^{Me}Ar^{NMe_2}$**, and **$BAr^{Bpin}Ar^{Me}Ar^{NMe_2}$** were collected on a BRUKER X8-APEX II diffractometer with a CCD area detector and multi-layer mirror monochromated Mo-K_α radiation. The structures were solved using the intrinsic phasing method (SHELXT),[5] refined with the SHELXL program[6] and expanded using Fourier techniques. All non-hydrogen atoms were refined anisotropically. Hydrogen atoms were included in structure factors calculations. All hydrogen atoms were assigned to idealized geometric positions.

Crystallographic data have been deposited with the Cambridge Crystallographic Data Center as supplementary publication no. (see **Table S1**). These data can be obtained free of charge from The Cambridge Crystallographic Data Centre *via* www.ccdc.cam.ac.uk/data_request/cif.

Crystal structures were depicted using Diamond 4.5 software by *Crystal Impact*.

Synthesis

General procedure (GP) 1: synthesis of potassium aryl trifluoroborates

$B(OMe)_3$ (2.0 equiv.) was dissolved in THF (0.50 mL/mmol) and cooled to 0 °C. At this temperature, an aryl Grignard reagent (1.0 equiv.) was added dropwise. The reaction was allowed to warm to r.t. overnight. After addition of Et_2O (3 mL/mmol), the resulting solid was collected by filtration in air and washed with Et_2O (9 mL/mmol). The solvent of the filtrate was removed *in vacuo*. The resulting oil was redisolved in THF (1 mL/mmol) and a solution of KHF_2 (3 equiv.) in water (0.5 mL/mmol) was added slowly. The reaction mixture was stirred at r.t. for 30 – 60 min. The solvent was evaporated to dryness *in vacuo*. The resulting solid was extracted with acetone and the solid removed by filtration. The solvent was removed *in vacuo.*

GP 2: synthesis of symmetrically substituted triarylboranes with six *ortho*-methyl groups

A) Reaction with aryl **Grignard** reagent: A potassium aryl trifluoroborate (1.0 equiv.) was added to an aryl Grignard (2.2 equiv.) in THF (1.7 mL/mmol). The reaction mixture was stirred in a sealed vessel at 90 °C for 2 d. After the addition of water, the aqueous phase was extracted with Et_2O. The combined organic phases were washed with brine and dried over $MgSO_4$.The solvent was removed *in vacuo*. The resulting crude product was purified *via* column chromatography and recrystallization using the conditions described below.

B) Reaction with aryl **lithium** reagent: A potassium aryl trifluoroborate (1.0 equiv.) was dissolved in THF (5 mL/mmol) and added dropwise to a solution of an aryl lithium reagent (2.2 equiv.) in THF (2 mL/mmol). The reaction mixture was stirred at r.t. for 2 d. After the addition of water, the aqueous phase was extracted with hexane. The combined organic phases were washed with water and brine, dried over $MgSO_4$ and the solvent was removed *in vacuo*. The resulting crude product was purified *via* column chromatography using the conditions described below.

C) Reaction with aryl **lithium** reagent after **activation** of the potassium aryl trifluoroborate: A potassium aryl trifluoroborate (1.0 equiv.) was dissolved in THF (5 mL/mmol) and trimethylsilyl chloride (5.0 equiv.) was added dropwise. The activation reaction was stirred at r.t. overnight. After removing the solvent *in vacuo*, the resulting solid was redisolved in Et_2O or THF (5 mL/mmol). This solution was added dropwise to a solution of an aryl lithium reagent (2.0 equiv.) in Et_2O or THF (3 mL/mmol), respectively. The reaction was stirred at r.t. for 2 d. After the addition of water, the aqueous phase was extracted with Et_2O. The combined organic phases were washed with brine, dried over $MgSO_4$ and the solvent was removed *in vacuo.*

The resulting crude product was purified *via* column chromatography using the conditions described below.

GP 3: one-pot synthesis of unsymmetrically substituted triarylboranes

A potassium aryl trifluoroborate (1.0 equiv.) was added to a solution of an aryl Grignard solution (1.0 equiv.) in THF (1.5 mL/mmol). After stirring at r.t. for 30 minutes, the solution was cooled to -78 °C and a solution of an aryl lithium reagent (1.2 equiv.) was added. The dark colored reaction mixture was allowed to warm to r.t. and stirred overnight. Water was added, and the aqueous phase was extracted with Et_2O. The combined organic phases were washed with brine and dried over $MgSO_4$. The solvent was removed *in vacuo* and the crude product was purified by automated flash column chromatography and recrystallization using the conditions described below.

GP 4: Sequential synthesis of unsymmetrically substituted triarylboranes

A) Synthesis of an unsymmetrically substituted diaryl**fluoroborane** with four *ortho*-methyl groups: A solution of an aryl Grignard reagent (1.3 equiv.) in THF (0.7 mL/mmol) was added to a solution of a potassium aryl trifluoroborate (1.0 equiv.) in THF (2 mL/mmol) and stirred at r.t. overnight. The solvent was removed *in vacuo* and the resulting solid was extracted with hot hexane. Unless otherwise stated, no further purification was performed.

B) Synthesis of an unsymmetrically substituted **triarylborane** with six *ortho*-methyl groups from an unsymmetrically substituted fluoroborane with four *ortho*-methyl groups: A solution of an unsymmetrically substituted diarylfluoroborane (1.0 equiv.) in THF (3 mL/mmol) was slowly added to a solution of an aryl lithium reagent (1.2 equiv.) in THF (2 mL/mmol). The dark colored reaction mixture was stirred at r.t. for 1 d. After addition of water, the aqueous phase was extracted with hexane. The combined organic phases were washed with water and brine, dried over $MgSO_4$ and the solvent was removed *in vacuo*. The resulting crude product was purified as described below.

GP 5: Ir-catalyzed CH borylation of triarylboranes

In a Young's-tube, a triarylborane (1.0 equiv.), B_2pin_2 (1.2 equiv.), $[Ir(COD)(\mu\text{-}OMe)]_2$ (2 mol%) and dtbpy (4 mol%) were dissolved in hexane (10 mL/mmol). The reaction mixture was stirred at 80 °C for 2 d, until TLC showed consumption of the starting material. After cooling to r.t., work up was performed as described below.

Potassium (2,6-dimethylphenyl) trifluoroborate (Ar^{H}BF$_3$K)

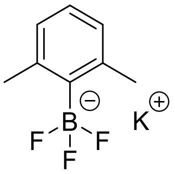

Compound **Ar^{H}BF$_3$K** was synthesized according to GP 1 using B(OMe)$_3$ (21.0 mL, 189 mmol), 2,6-dimethylphenyl magnesium bromide freshly prepared from 2-bromo-1,3-dimethylbenzene (12.6 mL, 94.3 mmol) and magnesium (5.7 g, 236 mmol) and KHF$_2$ (22.6 g, 289 mmol) yielding **Ar^{H}BF$_3$K** as a colorless solid (19.1 g, 90.0 mol, 96%). The NMR spectra match those reported in the literature.[7]

^{1}H NMR (300 MHz, acetone-d$_6$) δ = 6.81–6.74 (m, 1H), 6.73–6.66 (m, 2H), 2.38 (m, 6H) ppm.

^{11}B{^{1}H} NMR (96 MHz, acetone-d$_6$) δ = 4.2 (q, J = 60 Hz) ppm.

Potassium (mesityl) trifluoroborate (Ar^{Me}BF$_3$K)

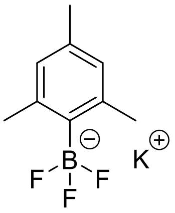

Compound **Ar^{Me}BF$_3$K** was synthesized according to GP 1 using B(OMe)$_3$ (11.4 mL, 100 mmol), 2,4,6-trimethylphenyl magnesium bromide freshly prepared from 2-bromo-1,3,5-trimethylbenzene (7.5 mL, 50.2 mmol) and magnesium (3.05 g, 125 mmol) and KHF$_2$ (11.7 g, 151 mmol). For further purification, the resulting solid was dissolved in acetone and precipitated with hexane, yielding **Ar^{Me}BF$_3$K** as a colorless solid (5.32 g, 23.5 mmol, 46%). The NMR spectra match those reported in the literature.[8]

^{1}H NMR (300 MHz, acetone-d$_6$) δ = 6.54 (s br, 2H), 2.34 (m, 6H), 2.12 (s br, 3H) ppm.

^{11}B{^{1}H} NMR (96 MHz, acetone-d$_6$) δ = 7.0 (q, J = 56 Hz) ppm.

Potassium (4-bromo-2,6-dimethylphenyl) trifluoroborate ($Ar^{Br}BF_3K$)

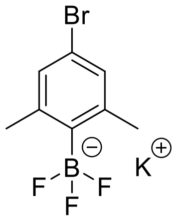

Compound **$Ar^{Br}BF_3K$** was synthesized according to GP 1 using $B(OMe)_3$ (3.6 mL, 32.2 mmol), 4-bromo-2,6-trimethylphenyl magnesium iodide freshly prepared from 4-bromo-1-iodo-2,6-dimethylbenzene (5.00 g, 16.1 mmol) and *i*-PrMgCl·LiCl (21.1 mL, 0.8 M, 16.9 mmol) and KHF_2 (3.77 g, 48.2 mmol). For further purification, the resulting solid was dissolved in acetone and precipitated with hexane, yielding **$Ar^{Br}BF_3K$** as a colorless solid (1.59 g, 5.46 mmol, 34%).

^{1}H NMR (500 MHz, acetone-d_6) δ = 6.89 (m, 2H), 2.38 (m, 6H) ppm.

^{11}B{^{1}H} NMR (160 MHz, acetone-d_6) δ = 4 (q, J = 57 Hz) ppm.

^{13}C{^{1}H} NMR (125 MHz, acetone-d_6) δ = 145.4, 129.7, 119.0, 23.4 (m) ppm.

^{19}F NMR (470 MHz, acetone-d_6) δ = -132.70 (m, 3F) ppm.

HRMS (ESI-) m/z: $[M-K]^-$ found: 252.9834; calc. for $[C_8H_8BBrF_3]^-$: 252.9829 (|Δ| = 1.98 ppm).

***Bis*[4-bromo-2,6-dimethylphenyl]-2,6-dimethylphenylborane ($BAr^HAr^{Br}Ar^{Br}$)**

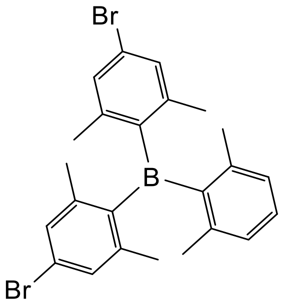

a) Compound **$BAr^HAr^{Br}Ar^{Br}$** was synthesized according to GP 2A using **Ar^HBF_3K** (313 mg, 1.47 mmol) and 4-bromo-2,6-trimethylphenyl magnesium iodide freshly prepared from 4-bromo-1-iodo-2,6-dimethylbenzene (1.01 g, 3.24 mmol) and *i*-PrMgCl·LiCl (4.2 mL, 0.8 M, 3.40 mmol). Automated flash column chromatography was performed using hexane as the eluent. The crude product was recrystallized from hot hexane yielding **$BAr^HAr^{Br}Ar^{Br}$** as a colorless solid (138 mg, 0.29 mmol, 19%).

b) Compound **$BAr^HAr^{Br}Ar^{Br}$** was synthesized according to GP 2C using **Ar^HBF_3K** (500 mg, 2.36 mmol) and trimethylsilyl chloride (1.5 mL, 11.8 mmol) in THF for the activation. Afterwards, the solvent was changed to Et_2O and the solution was added to the 4-bromo-2,6-dimethyphenyllithium freshly prepared from 4-bromo-1-iodo-2,6-dimethylbenzene (1.47 g, 4.72 mmol) and *n*-buthyllithium (1.9 mL, 2.5 M, 4.75 mmol) in Et_2O. The reaction mixture was stirred at -78 °C for 8 h. Afterwards, the reaction was stirred at r.t. for 2 d. After work up as described above, column chromatography was performed using hexane as the eluent. The crude product was recrystallized from hot hexane yielding **$BAr^HAr^{Br}Ar^{Br}$** as a colorless solid (586 mg, 1.21 mmol, 51%).

^{1}H NMR (300 MHz, CD_2Cl_2) δ = 7.16 (m, 1H), 7.12 (br s, 4H), 6.94 (m, 2H), 2.00 (s, 6H), 1.99 (s, 6H), 1.97 (s, 6H) ppm.

^{11}B{^{1}H} NMR (96 MHz, CD_2Cl_2) δ = 78 (br) ppm.

^{13}C{^{1}H} NMR (75 MHz, CD_2Cl_2) δ = 146.3, 145.6, 143.2, 143.0, 140.8, 131.0, 131.0, 130.5, 128.3, 124.3, 23.1, 22.9, 22.8 ppm.

HRMS (EI^+) m/z: $[M-C_8H_9]^+$ found: 378.9684; calc. for $[C_{16}H_{16}BBr_2]$: 378.9686 (|Δ| = 0.53 ppm).

Elemental analysis Calc. (%) for $C_{24}H_{25}BBr_2$: C 59.55, H 5.21; found: C 59.51, H 5.35.

Bis[4-(_N_,_N_-dimethylamino)-2,6-dimethylphenyl]-2,6-dimethylphenylborane ($BAr^HAr^{NMe_2}Ar^{NMe_2}$)

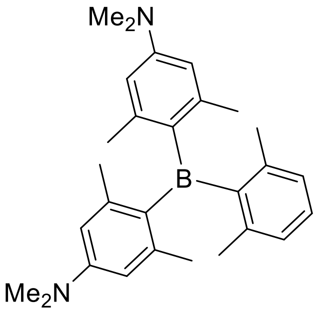

Compound **$BAr^HAr^{NMe_2}Ar^{NMe_2}$** was synthesized according to GP 2B using **Ar^HBF_3K** (2.56 g, 12.1 mmol) and 4-(*N*,*N*-dimethylamino)-2,6-dimethylphenyllithum (4.14 g, 26.7 mmol).

The crude product was purified by distillation (2×10^{-2} mbar, 90 °C) and column chromatography (aluminum oxide, basic, 10% EtOAc in hexane) yielding **$BAr^HAr^{NMe_2}Ar^{NMe_2}$** as a yellow solid (2.13 g, 5.17 mmol, 43%). The NMR spectrum matches that reported in literature.[9]

^{1}H NMR (300 MHz, CD_2Cl_2) δ = 7.12 (dd, *J* = 7.6 Hz, 1H), 6.92 (d, *J* = 7.6 Hz, 2H), 6.36 (s, 4H), 2.98 (s, 12H), 2.08 (s, 6H), 2.06 (s, 6H), 1.97 (s, 6H) ppm.

(4-Bromo-2,6-dimethylphenyl)-(mesityl)-2,6-dimethylphenylborane ($BAr^{H}Ar^{Br}Ar^{Me}$)

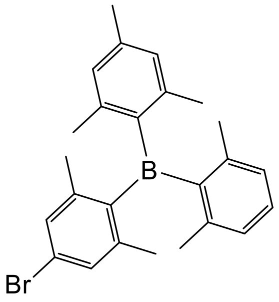

Compound **$BAr^{H}Ar^{Br}Ar^{Me}$** was synthesized according to GP 3 using **$Ar^{Me}BF_3K$** (2.04 g, 9.02 mmol), 4-bromo-2,6-trimethylphenyl magnesium iodide freshly prepared from 4-bromo-1-iodo-2,6-dimethylbenzene (2.81 g, 9.02 mmol) and *i*-PrMgCl·LiCl (11.8 mL, 0.8 M, 9.47 mmol) and 2,6-dimethylphenyllithium freshly prepared from 2-bromo-1,3-dimethylbenzene (1.5 mL, 10.8 mmol) and *t*-butyllithium (12.7 mL, 1.7 M, 21.7 mmol). Automated flash column chromatography was performed using hexane as the eluent. The crude product was recrystallized from hot hexane yielding **$BAr^{H}Ar^{Br}Ar^{Me}$** as a colorless solid (1.32 g, 3.16 mmol, 35%).

^{1}H NMR (300 MHz, CD_2Cl_2) δ = 7.14 (m, 1H), 7.11 (m, 2H), 6.93 (m, 2H), 6.77 (s, 2H), 2.27 (s, 3H), 2.00 (s, 6H), 1.99 (s, 3H), 1.97 (s, 6H) 1.96 (s, 3H) ppm.

^{11}B{^{1}H} NMR (96 MHz, CD_2Cl_2) δ = 78 (br) ppm.

^{13}C{^{1}H} NMR (75 MHz, CD_2Cl_2) δ = 147.1, 146.4, 143.7, 143.1, 143.0, 141.2, 141.0, 140.8, 140.6, 140.4, 130.8, 130.7, 130.0, 129.2, 129.2, 128.1, 128.1, 123.8, 23.0, 23.0, 22.9, 22.8, 22.7, 21.4 ppm.

HRMS (EI^{+}) m/z: $[M-C_8H_9]^{+}$ found: 315.0736; calc. for $[C_{17}H_{19}BBr]$: 315.0737 (|Δ| = 0.32 ppm).

Elemental analysis Calc. (%) for $C_{25}H_{28}BBr$: C 71.63, H 6.73; found: C 72.03, H 7.01.

(4-Bromo-2,6-dimethylphenyl)-[(4-trimethylsilyl)-2,6-dimethylphenyl]-2,6-dimethylphenylborane ($BAr^{H}Ar^{Br}Ar^{SiMe_3}$)

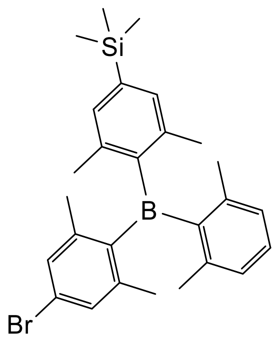

Compound $\mathbf{BAr^{H}Ar^{Br}Ar^{SiMe_3}}$ was synthesized according to GP 3 using $\mathbf{Ar^{H}BF_3K}$ (2.97 g, 14.0 mmol), 4-bromo-2,6-trimethylphenyl magnesium iodide freshly prepared from 4-bromo-1-iodo-2,6-dimethylbenzene (4.35 g, 14.0 mmol) and *i*-PrMgCl·LiCl (11.3 mL, 1.3 M, 14.7 mmol) and 4-trimethylsilyl-2,6-dimethylphenyllithium freshly prepared from 2-bromo-1,3-dimethyl-4-trimethylsilylbenzene (4.3 mL, 16.8 mmol) and *t*-butyllithium (19.8 mL, 1.7 M, 33.6 mmol). Automated flash column chromatography was performed using EtOAc in hexane (0% to 10%) as the eluent. The crude product was recrystallized from hot hexane yielding $\mathbf{BAr^{H}Ar^{Br}Ar^{SiMe_3}}$ as a colorless solid (1.07 g, 2.24 mmol, 16%).

^{1}H NMR (500 MHz, CD_2Cl_2) δ = 7.15 (m, 1H), 7.12 (m, 2H), 7.07 (m, 2H), 6.93 (m, 2H), 2.02 (s, 3H), 2.02 (s, 3H), 2.00 (s, 6H), 1.99 (s, 3H), 1.98 (s, 3H), 0.25 (s, 9H) ppm.

^{11}B{^{1}H} NMR (160 MHz, CD_2Cl_2) δ = 78 (br) ppm.

^{13}C{^{1}H} NMR (125 MHz, CD_2Cl_2) 147.1, 146.8, 146.1, 143.1, 143.1, 142.8, 140.9, 140.7, 139.6, 139.4, 133.2, 133.1, 130.8, 130.8, 130.2, 128.2, 123.9, 23.1, 23.1, 23.0, 23.0, 22.8, 22.7, -1,11 ppm.

HRMS (EI^+) m/z: $[M+H]^+$ found: 479.1756; calc. for $[C_{27}H_{34}BBrSi_4]$: 479.1759 (|Δ| = 0.62 ppm).

Elemental analysis Calc. (%) for $C_{27}H_{34}BBrSi$: C 67.93, H 7.18; found: C 68.52, H 7.17.

[4-(*N*,*N*-dimethylamino)-2,6-dimethylphenyl]-(2,6-dimethylphenyl)-fluoroborane ($BFAr^{H}Ar^{NMe_2}$)

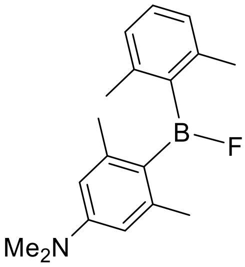

Compound **$BFAr^{H}Ar^{NMe_2}$** was synthesized according to GP 4A using **$Ar^{H}BF_3K$** (5.00 g, 23.6 mmol) and 4-*N*,*N*-dimethylamino-2,6-dimethlyphenyl magnesium bromide freshly prepared from 4-bromo-*N*,*N*,2,6-tetramethylaniline (6.99 g, 60.6 mmol) and magnesium (1.49 g, 61.3 mol).

The crude product was extracted a second time with hot hexane. The solvent was removed *in vacuo* yielding **$BFAr^{H}Ar^{NMe_2}$** as a yellow oil (5.57 g, 19.7 mmol, 83%) which was used without further purification. It contained a small amount of residual *N*,*N*,3,5-tetramethylaniline.

^{1}H NMR (500 MHz, C_6D_6) δ = 7.13 (m, 1H), 6.93 (m, 2H), 6.28 (s, 2H), 2.46 (s, 6H), 2.45 (s, 3H), 2.45 (s, 3H), 2.34 (s, 3H), 2.34 (s, 3H) ppm. (*N*,*N*,3,5-tetramethylaniline: δ = 6.48 (s, 1H), 6.42 (s, 2H), 2.59 (s, 6H), 2.26 (s, 6 H) ppm.)

^{11}B{^{1}H} NMR (160 MHz, C_6D_6) δ = 52.3 (s) ppm.

^{13}C{^{1}H} NMR (125 MHz, C_6D_6) δ = 152.8, 147.9, 147.8, 141.6, 140.0, 140.0, 129.5, 129.5, 127.4, 122.5, 112.3, 39.3, 24.4, 24.3, 22.3 ppm. (*N*,*N*,3,5-tetramethylaniline: δ = 151.4, 138.4, 119.4, 111.4, 40.5, 22.0 ppm.)

^{19}F NMR (470 MHz, C_6D_6) δ = -23.7 (s) ppm.

HRMS (LIFDI) m/z: $[M]^+$ found: 283.1898; calc. for [$C_{18}H_{23}BFN$]: 283.1902 (|Δ| = 1.41 ppm).

[4-(*N*,*N*-dimethylamino)-2,6-dimethylphenyl]-(mesityl)-2,6-dimethylphenylborane ($BAr^HAr^{Me}Ar^{NMe_2}$)

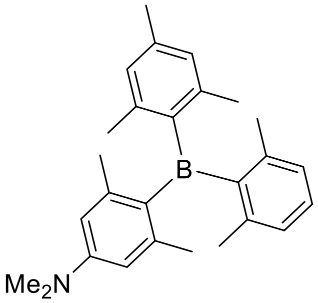

Compound **$BAr^HAr^{Me}Ar^{NMe_2}$** was synthesized according to GP 4B using **$BFAr^HAr^{NMe_2}$** (5.57 g, 19.7 mmol) and 2,4,6-trimethylphenyllithum freshly prepared from 2,4,6-trimethylphenyl bromide (5.6 mL, 28.3 mmol) and *tert*-butyllithium (56.6 mmol, 1.7 M, 33.0 mL).

The crude product was purified by distillation (5×10^{-2} mbar, 80 °C) and column chromatography (aluminum oxide, neutral, pure hexane, then pure CH_2Cl_2) yielding **$BAr^HAr^{Me}Ar^{NMe_2}$** as a yellow-greenish solid (4.30 g, 11.2 mmol, 48%).

^{1}H NMR (500 MHz, CD_2Cl_2) δ = 7.20 (m, 1H), 6.90 (m, 2H), 6.75 (s, 2H), 6.32 (s, 2H), 3.00 (s, 6H), 2.27 (s, 3H), 2.02 (br s, 6H), 1.97 (s, 6H), 1.95 (s, 3H) ppm.

^{11}B{^{1}H} NMR (160 MHz, CD_2Cl_2) δ = 75 (br) ppm.

^{13}C{^{1}H} NMR (125 MHz, CD_2Cl_2) δ = 152.0, 148.8, 145.4, 143.7, 143.6, 140.8, 140.5, 140.3, 139.0, 135.2, 129.0, 128.8, 127.7, 111.9, 111.9, 40.1, 24.0, 23.9, 23.1, 22.8, 22.7, 21.3 ppm.

HRMS (EI^+) m/z: $[M+H]^+$ found: 384.2853; calc. for $[C_{27}H_{35}BN]$: 384.2857 ($|\Delta|$ = 1.04 ppm).

Elemental analysis Calc. (%) for $C_{27}H_{34}BN$: C 84.59, H 8.94, N 3.65; found: C 84.36, H 9.35, N 3.75.

Bis[4-bromo-2,6-dimethylphenyl]-[4-(pinacolboryl)-2,6-dimethylphenyl]borane ($BAr^{Bpin}Ar^{Br}Ar^{Br}$)

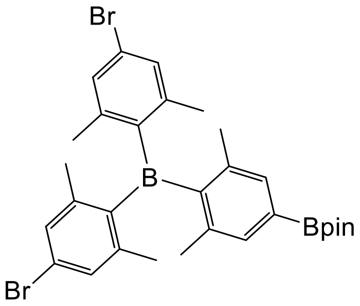

Compound **$BAr^{Bpin}Ar^{Br}Ar^{Br}$** was synthesized according to GP 5 using **$BAr^{H}Ar^{Br}Ar^{Br}$** (115 mg, 238 μmol), B_2pin_2 (72 mg, 285 μmol), dtbpy (3 mg, 4.75 μmol), and $[Ir(COD)(\mu\text{-OMe})]_2$ (3 mg, 9.50 μmol).

Work up was performed by removing the solvent *in vacuo*. The crude product was purified by automated flash column chromatography using hexane/EtOAc (10:1 to 9:1) as the eluent. The resulting compound was recrystallized from hexane yielding **$BAr^{Bpin}Ar^{Br}Ar^{Br}$** as a colorless solid (98 mg, 0.16 mmol, 68%).

^{1}H NMR (300 MHz, CD_2Cl_2) δ = 7.32 (s, 2H), 7.11 (m, 4H), 2.01 (s, 6H), 1.99 (s, 6H), 1.96 (s, 6H), 1.32 (s, 12H) ppm.

^{11}B{^{1}H} NMR (96 MHz, CD_2Cl_2) δ = 78 (br), 31 (br) ppm.

^{13}C{^{1}H} NMR (75 MHz, CD_2Cl_2) δ = 149.4, 145.3, 143.2, 143.1, 139.8, 134.4, 131.0, 131.0, 124.4, 84.2, 25.1, 23.0, 22.9, 22.8 ppm.

HRMS (EI$^-$) m/z: $[M]^-$ found: 610.1262; calc. for $[C_{30}H_{36}B_2Br_2O_2]$: 610.1253 (|Δ| = 1.48 ppm).

Elemental analysis Calc. (%) for $C_{30}H_{36}B_2Br_2O_2$: C 59.07, H 5.95; found: C 59.65, H 6.15.

(4-Bromo-2,6-dimethylphenyl)-(mesityl)-[4-(pinacolboryl)-2,6-dimethylphenyl]borane ($\mathbf{BAr^{Bpin}Ar^{Br}Ar^{Me}}$)

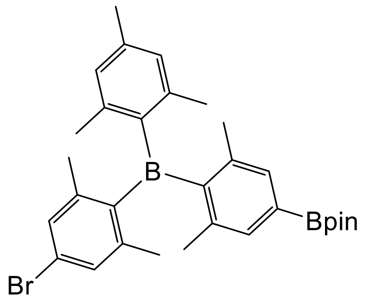

Compound $\mathbf{BAr^{Bpin}Ar^{Br}Ar^{Me}}$ was synthesized according to GP 5 using $\mathbf{BAr^{H}Ar^{Br}Ar^{Me}}$ (800 mg, 1.91 mmol), B_2pin_2 (582 mg, 2.29 mmol), dtbpy (20 mg, 76.3 *μ*mol), and [Ir(COD)(*μ*-OMe)]$_2$ (25 mg, 38.2 *μ*mol).

The work up was performed by removing the solvent *in vacuo*. The crude product was purified by automated flash column chromatography using hexane/EtOAc (10:1 to 9:1) as the eluent yielding $\mathbf{BAr^{Bpin}Ar^{Br}Ar^{Me}}$ as a colorless solid (542 mg, 0.99 mmol, 52%).

^{1}H NMR (300 MHz, CD_2Cl_2) *δ* = 7.30 (s, 2H), 7.10 (s, 2H), 6.77 (m, 2H), 2.27 (s, 3H), 2.01 (s, 6H), 1.99 (s, 3H), 1.97 (s, 3H) 1.95 (s, 3H), 1.94 (s, 3H), 1.32 (s, 12H) ppm.

^{11}B{^{1}H} NMR (96 MHz, CD_2Cl_2) *δ* = 78 (br), 31 (br) ppm.

^{13}C{^{1}H} NMR (75 MHz, CD_2Cl_2) *δ* = 150.2, 146.1, 143.4, 143.1, 143.0, 141.2, 141.1, 140.5, 139.8, 139.6, 134.2, 134.2, 130.8, 130.8, 129.2, 123.9, 84.1, 25.1, 23.1, 23.0, 22.9, 22.8, 22.7, 21.37 ppm.

HRMS (EI^+) m/z: $[M-C_8H_8Br]^+$ found: 361.2503; calc. for $[C_{23}H_{31}B_2BrO_2]$: 361.2505 (|Δ| = 0.55 ppm).

Elemental analysis Calc. (%) for $C_{31}H_{39}B_2BrO_2$: C 68.30, H 7.21; found: C 68.34, H 7.43.

(4-Bromo-2,6-dimethylphenyl)-[4-(trimethylsilyl)-2,6-dimethylphenyl]-[4-(pinacolboryl)-2,6-dimethylphenyl]borane ($BAr^{Bpin}Ar^{Br}Ar^{SiMe_3}$)

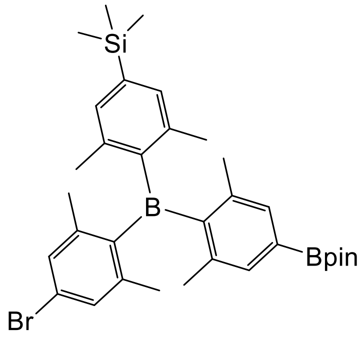

Compound $\mathbf{BAr^{Bpin}Ar^{Br}Ar^{SiMe_3}}$ was synthesized according to GP 5 using $\mathbf{BAr^{H}Ar^{Br}Ar^{SiMe_3}}$ (546 mg, 1.14 mmol), B_2pin_2 (349 mg, 1.37 mmol), dtbpy (12 mg, 45.8 *µ*mol), and $[Ir(COD)(\mu\text{-}OMe)]_2$ (15 mg, 22.9 *µ*mol).

The work up was performed by passing the reaction mixture through a silica pad using EtOAc as the eluent. The crude product was recrystallized from EtOAc yielding $\mathbf{BAr^{Bpin}Ar^{Br}Ar^{SiMe_3}}$ as a colorless solid (388 mg, 0.64 mmol, 56%).

^{1}H NMR (300 MHz, CD_2Cl_2) δ = 7.31 (m, 2H), 7.11 (s, 2H), 7.06 (m, 2H), 2.03 (s, 3H), 2.01 (s, 6H), 1.98 (s, 3H), 1.97 (s, 6H), 1.32 (s, 12H), 0.24 (s, 9H) ppm.

^{11}B{^{1}H} NMR (96 MHz, CD_2Cl_2) δ = 78 (br), 31 (br) ppm.

^{13}C{^{1}H} NMR (75 MHz, CD_2Cl_2) δ = 150.0, 146.9, 145.9, 143.2, 143.1, 142.9, 139.9, 139.7, 139.6, 139.5, 134.2, 133.2, 133.2, 130.8, 130.8, 124.0, 84.1, 25.1, 23.1, 23.0, 22.9, 22.9, 22.8, 22.7, -1.1 ppm.

HRMS (EI^+) m/z: $[M+H]^+$ found: 603.2622; calc. for $[C_{33}H_{47}B_2BrO_2Si]$: 603.2631 (|Δ| = 1.49 ppm).

Elemental analysis: Calc. (%) for $C_{33}H_{45}B_2BrO_2Si$: C 65.70, H 7.52; found: C 65.31, H 7.58.

[4-(*N*,*N*-dimethylamino)-2,6-dimethylphenyl]-(mesityl)-[4-(pinacolboryl)-2,6-dimethylphenyl]borane (BAr^{Bpin}Ar^{Me}Ar^{NMe_2})

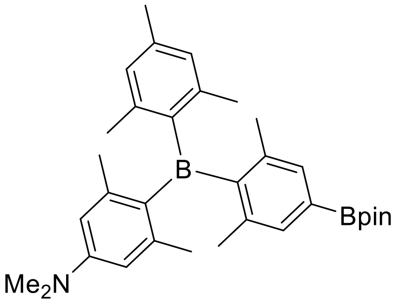

Compound **BAr^{Bpin}Ar^{Me}Ar^{NMe_2}** was synthesized according to GP 5 using **BAr^{H}Ar^{Me}Ar^{NMe_2}** (1.08 g, 2.82 mmol), B_2pin_2 (822 mg, 3.24 mmol), dtbpy (24 mg, 88 *μ*mol), and [Ir(COD)(*μ*-OMe)]$_2$ (24 mg, 36 *μ*mol).

The work up was performed by removing the solvent *in vacuo*. The crude product was purified by automated flash column chromatography (NH-column) using EtOAc in hexane (0–100%) as the eluent yielding **BAr^{Bpin}Ar^{Me}Ar^{NMe_2}** as a yellow solid (1.18 g, 2.32 mmol, 89%).

^{1}H NMR (500 MHz, CD_2Cl_2) *δ* = 7.30 (s, 2H), 6.75 (s, 2H), 6.32 (m, 2H), 2.97 (s, 6H), 2.27 (s, 3H), 2.12–1.89 (m, 18H), 1.34 (s, 12H) ppm.

^{11}B{^{1}H} NMR (160 MHz, CD_2Cl_2) *δ* = 75.7 (br s), 31.0 (s) ppm.

^{13}C{^{1}H} NMR (125 MHz, CD_2Cl_2) *δ* = 152.3, 152.1, 145.2, 143.8, 143.7, 139.5, 139.1, 135.0, 133.9, 111.9, 84.0, 40.1, 25.2, 24.1, 24.0, 21.4 ppm.

HRMS (EI$^+$) m/z: [M+H]$^+$ found: 510.3702; calc. for [$C_{33}H_{45}B_2NO_2$]: 510.3709 (|Δ| = 1.37 ppm).

Elem. Anal. Calc. (%) for $C_{33}H_{45}B_2NO_2$: C 77.82, H 8.91, N 2.75; found: C 77.50, H 9.20, N 2.85.

4-Bromo-2,6-dimethylphenyl-4-iodo-2,6-dimethylphenyl-2,6-dimethylphenylborane ($BAr^{H}Ar^{Br}Ar^{I}$)

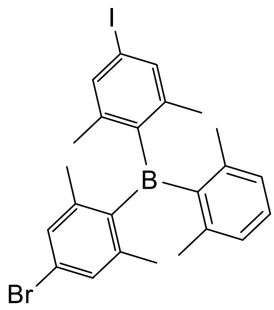

Compound **$BAr^{H}Ar^{Br}Ar^{SiMe_3}$** (50.0 mg, 105 μmol, 1.0 eq) was dissolved in CH_2Cl_2 (2 mL) and cooled
to -10 °C. A solution of ICl (18.7 mg, 115 μmol, 1.1 eq) in CH_2Cl_2 (2 mL) was added dropwise. The reaction mixture was stirred for 30 min. The volatiles were removed giving **$BAr^{H}Ar^{Br}Ar^{I}$** as a colorless solid (56 mg, ~99%).

^{1}H NMR (300 MHz, CD_2Cl_2, r.t., ppm): δ = 7.35 (s, 2H), 7.17 (m, 1H), 6.95 (s, 2H), 6.94 (m, 2H), 2.00 (s, 6H), 2.00 (s, 3H), 1.97 (s, 3H), 1.96 (s, 3H), 1.95 (s, 3H).

^{11}B{^{1}H} NMR (96 MHz, CD_2Cl_2, r.t., ppm): δ = 78.

^{13}C{^{1}H} NMR (75 MHz, CD_2Cl_2, r.t., ppm): δ = 146.3, 146.1, 145.6, 143.2, 143.0, 142.9, 142.8, 140.8, 137.0, 137.0, 131.0, 131.0, 130.5, 128.3, 124.3, 97.0, 23.1, 23.1, 22.9, 22.7, 22.7, 22.5.

HRMS (EI^{+}) m/z: $[M-C_8H_9]^{+}$ found: 424.9562; calc. for $[C_{16}H_{16}BBrI]$: 424.9568 (|Δ| = 1.41 ppm).

Elem. Anal. Calc. (%) for $C_{24}H_{25}BBrI$: C 54.28, H 4.75; found: C 53.96, H 4.89.

4-Bromo-2,6-dimethylphenyl-4-[4-(methoxycarbonyl)phenyl]-2,6-dimethylphenyl-2,4,6-trimethylphenylborane ($BAr^{Ph}Ar^{Br}Ar^{Me}$)

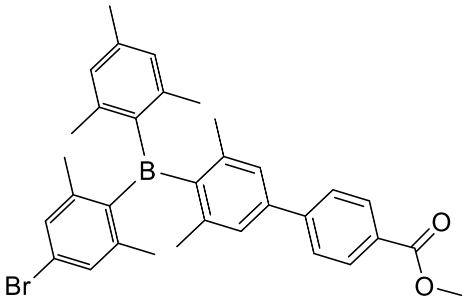

$BAr^{Bpin}Ar^{Br}Ar^{Me}$ (50.0 mg, 91.7 *μ*mol, 1.0 eq), methyl 4-iodobenzoate (36.1 mg, 138 *μ*mol, 1.5 eq), SPhos (5 mg, 12.8 *μ*mol, 14 mol%), $Pd_2(dba)_3{\cdot}CHCl_3$ (7 mg, 6.4 *μ*mol, 7 mol%) and $CsCO_3$ (179 mg, 550 mmol, 6.0 eq) were dissolved in a mixture of toluene (2 mL) and water (1 mL). The mixture was stirred for 18 h at 85 °C, until the starting material was consumed according to TLC (EtOAc/hexane 1:9). The organic phase was separated and the aqueous phase was extracted with CH_2Cl_2 (2 x 5 mL). The organic phases were combined and, after removing the solvent *in vacuo*, the solid was purified by automated flash column chromatography (stepwise gradient from 0% to 4% EtOAc in hexane) to afford compound **$BAr^{Ph}Ar^{Br}Ar^{Me}$** as a colorless solid (40 mg, 79%).

^{1}H NMR (300 MHz, CD_2Cl_2, r.t., ppm): *δ* = 8.05 (m, 2H), 7.72 (m, 2H), 7.25 (s, 2H), 7.13 (s, 2H), 6.79 (s, 2H), 3.91 (s, 3H), 2.28 (s, 3H), 2.09 (s, 6H), 2.02 (s, 6H), 2.01 (s, 3H), 2.00 (s, 3H).

^{11}B{^{1}H} NMR (96 MHz, CD_2Cl_2, r.t., ppm): *δ* = 77.

^{13}C{^{1}H} NMR (75 MHz, CD_2Cl_2, r.t., ppm): *δ* = 167.2, 147.0, 146.2, 145.5, 143.6, 143.1, 143.0, 141.7, 141.5, 141.2, 141.1, 140.9, 140.6, 130.8, 130.3, 129.5, 129.2, 129.2, 127.1, 126.8, 123.9, 52.4, 23.2, 23.1, 22.8, 21.4.

HRMS (EI^+) m/z: $[M+H]^+$ found: 553.1899; calc. for $[C_{33}H_{35}BBrO_2]$: 553.1908 (|Δ| = 1.63 ppm).

Elem. Anal. Calc. (%) for $C_{33}H_{34}BBrO_2$: C 71.63, H 6.19; found: C 71.25, H 6.33.

Single-crystal X-ray Diffraction Data

Table S 1. Single-crystal X-ray diffraction data and structure refinements of **BAr^{H}Ar^{Br}Ar^{Br}**, **BAr^{H}Ar^{Br}Ar^{SiMe_3}**, **BAr^{H}Ar^{Me}Ar^{NMe_2}** and **BAr^{Bpin}Ar^{Me}Ar^{NMe_2}**.

Data	**BAr^{H}Ar^{Br}Ar^{Br}**	**BAr^{H}Ar^{Br}Ar^{SiMe_3}**	**BAr^{H}Ar^{Me}Ar^{NMe_2}**	**BAr^{Bpin}Ar^{Me}Ar^{NMe_2}**
CCDC number	2045568	2045569	2045567	2045566
Empirical formula	$C_{24}H_{25}BBr_2$	$C_{27}H_{34}BBrSi$	$C_{55}H_{70}B_2Cl_2N_2$	$C_{33}H_{45}B_2NO_2$
Formula weight (g·mol^{-1})	484.07	477.35	851.65	509.32
Temperature (K)	100(2)	100(2)	100(2)	296(2)
Radiation, λ (Å)	Mo-K$_{\alpha}$ 0.71073	Mo-K$_{\alpha}$ 0.71073	Mo-K$_{\alpha}$ 0.71073	Mo$_{K\alpha}$0.71073
Crystal system	Monoclinic	Triclinic	Triclinic	Monoclinic
Space group	*Cc*	$P\bar{1}$	$P\bar{1}$	$P2_1/c$
Unit cell dimensions				
a (Å)	11.538(7)	8.307(5)	8.193(4)	8.102(5)
b (Å)	12.002(7)	12.676(6)	16.849(8)	22.572(5)
c (Å)	16.047(10)	13.537(7)	18.995(9)	16.990(4)
α (°)	90	106.688(15)	113.933(11)	90
β (°)	107.553(17)	98.395(15)	92.463(16)	91.67(2)
γ (°)	90	107.363(14)	95.778(10)	90
Volume (Å^3)	2119(2)	1260.7(12)	2374.3(19)	3106(2)
Z	4	2	2	4
Calculated density (Mg·m^{-3})	1.518	1.258	1.191	1.089
Abs. coefficient (mm^{-1})	3.832	1.689	0.176	0.065
F(000)	976	500	916	1104
Theta range for collection	2.511 to 26.298°	2.618 to 28.386°	1.367 to 26.395°	1.501 to 26.773°
Reflections collected	31909	52032	79205	54729
Independent reflections	4298	6242	9726	6616
Minimum/maximum transmission	0.3396/0.4778	0.6240/ 0.7457	0.7101/0.7454	0.7208/0.7449
Refinement method	Full-matrix least-squares on F^2	Full-matrix least-squares on F^2	Full-matrix least-squares on F^2	Full-matrix least-squares on F^2
Data / parameters / restrains	4298 / 251 / 2	6242 / 280 / 0	9726 / 596 / 120	6616 / 356 / 0
Goodness-of-fit on F^2	1.023	1.057	1.016	1.034
Final R indices [I>2σ(I)]	R_1 = 0.0277, wR^2 = 0.0686	R_1 = 0.0319, wR^2 = 0.0818	R_1 = 0.0502, wR^2 = 0.1184	R_1 = 0.0437, wR^2 = 0.1128
R indices (all data)	R_1 = 0.0286, wR^2 = 0.0689	R_1 = 0.0401, wR^2 = 0.0854	R_1 = 0.0846, wR^2 = 0.1361	R_1 = 0.0527, wR^2 = 0.1202
Max./min. residual electron density (e·Å^{-3})	0.874 / –0.284	0.491 / –0.567	0.390 / –0.314	0.332 / –0.252

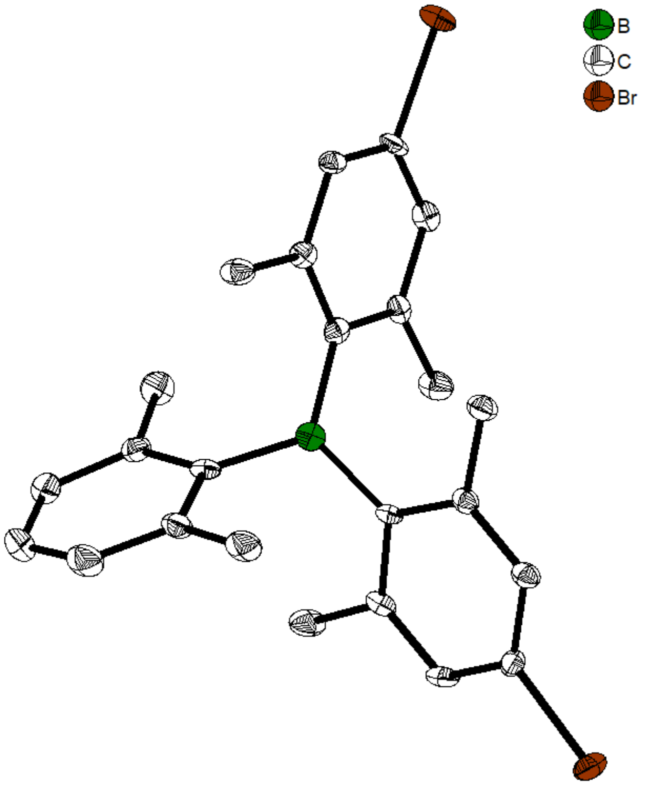

Figure S1. Molecular structure of **BAr^{H}Ar^{Br}Ar^{Br}** in the solid state at 100 K. Atomic displacement ellipsoids are drawn at the 50% probability level, and H atoms are omitted for clarity.

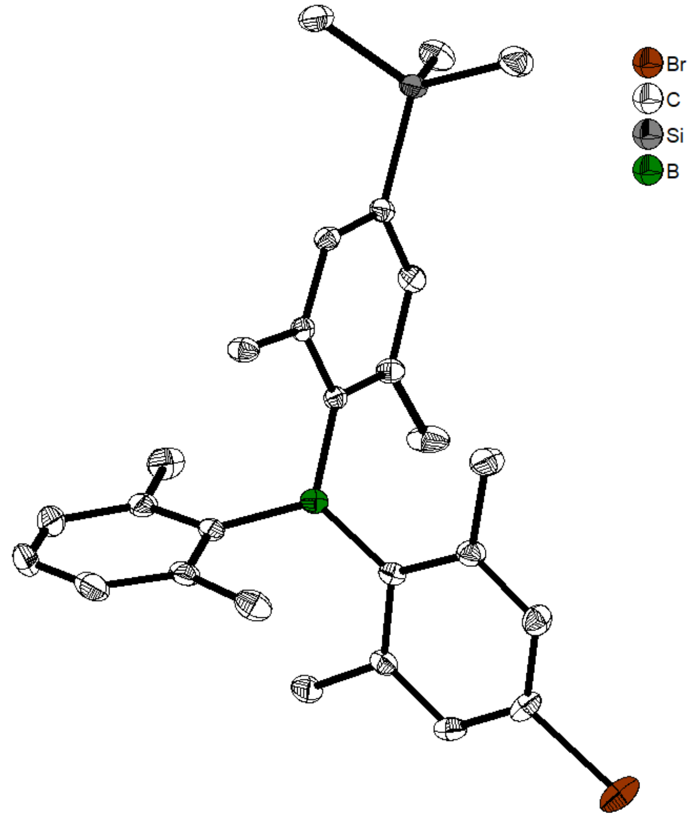

Figure S2. Molecular structure of **$BAr^{H}Ar^{Br}Ar^{SiMe_3}$** in the solid state at 100 K. Atomic displacement ellipsoids are drawn at the 50% probability level, and H atoms are omitted for clarity.

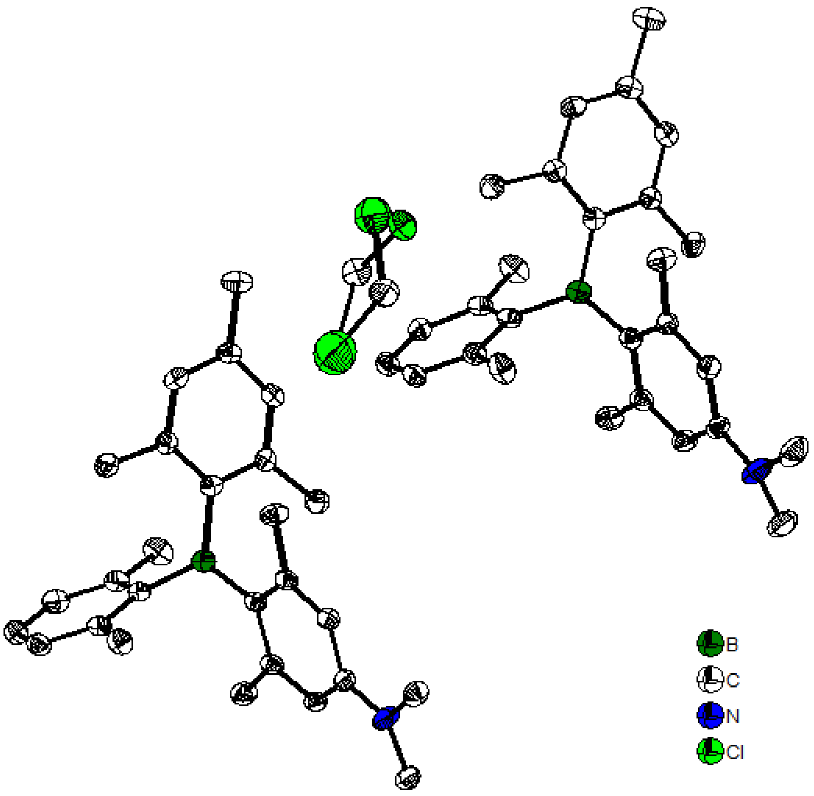

Figure S3. Molecular structure of **BAr^{H}Ar^{Me}Ar^{NMe_2}** in the solid state at 100 K. Atomic displacement ellipsoids are drawn at the 50% probability level, and H atoms are omitted for clarity.

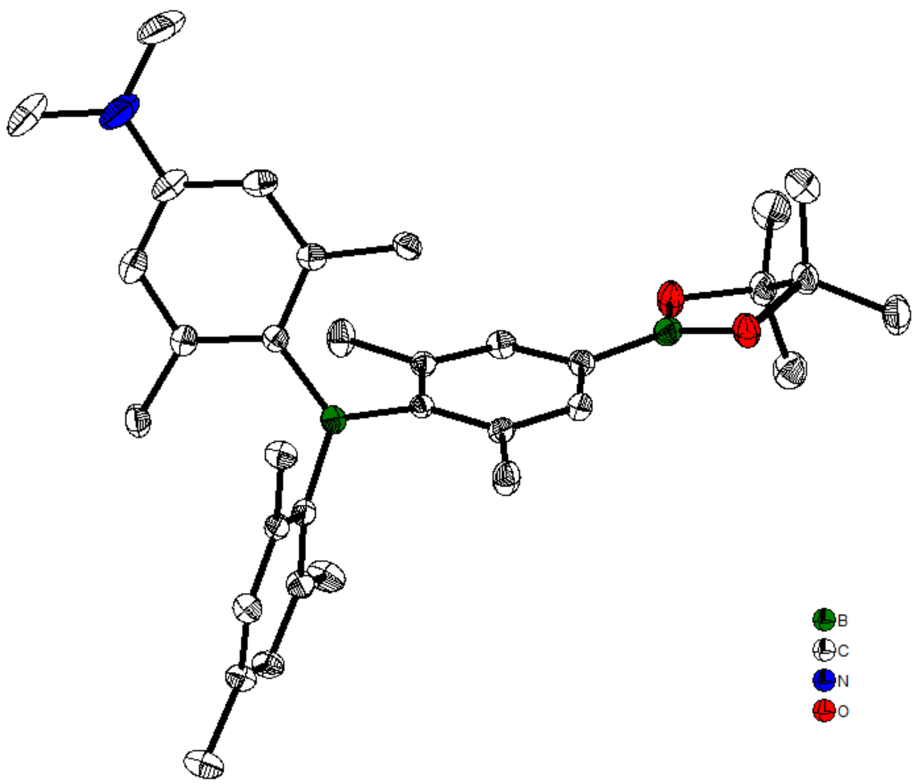

Figure S4. Molecular structure of **BAr^{Bpin}Ar^{Me}Ar^{NMe_2}** in the solid state at 100 K. Atomic displacement ellipsoids are drawn at the 50% probability level, and H atoms are omitted for clarity.

NMR Spectra

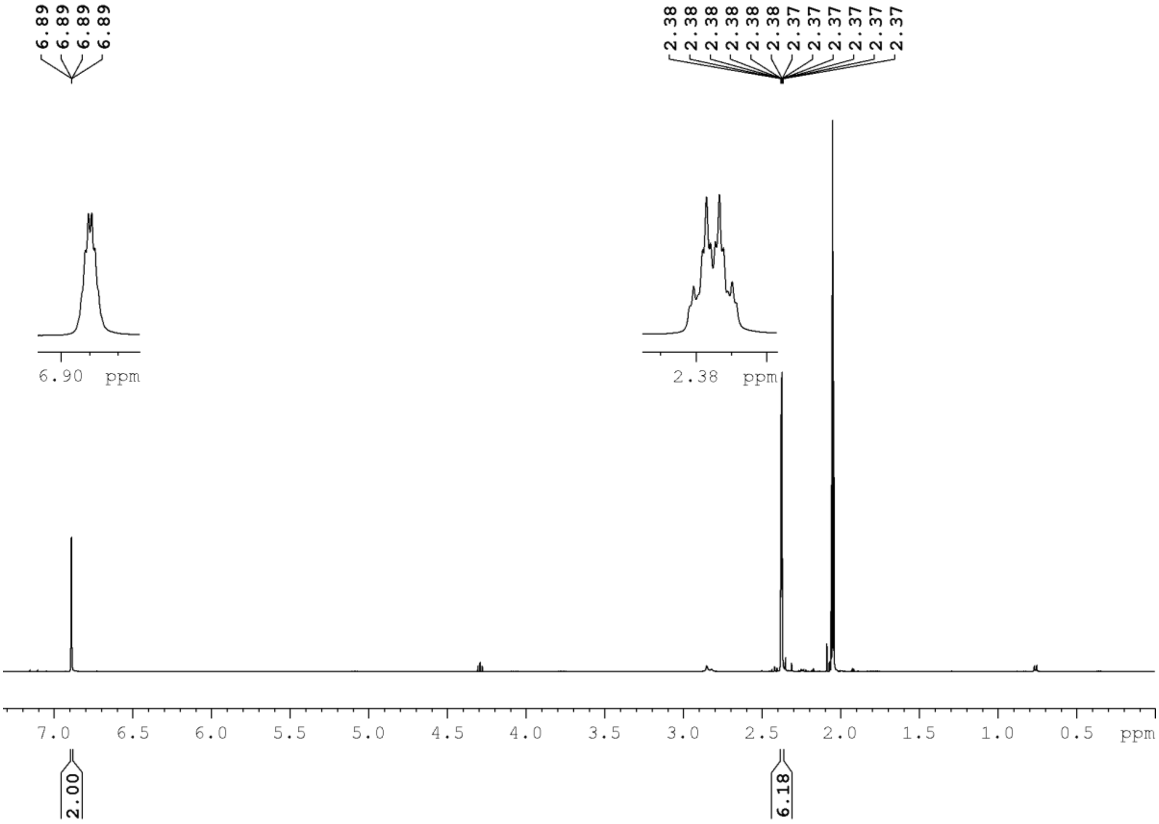

Figure S4. ^{1}H NMR spectrum of **Ar^{Br}BF$_3$K** in acetone-d$_6$ at 500 MHz.

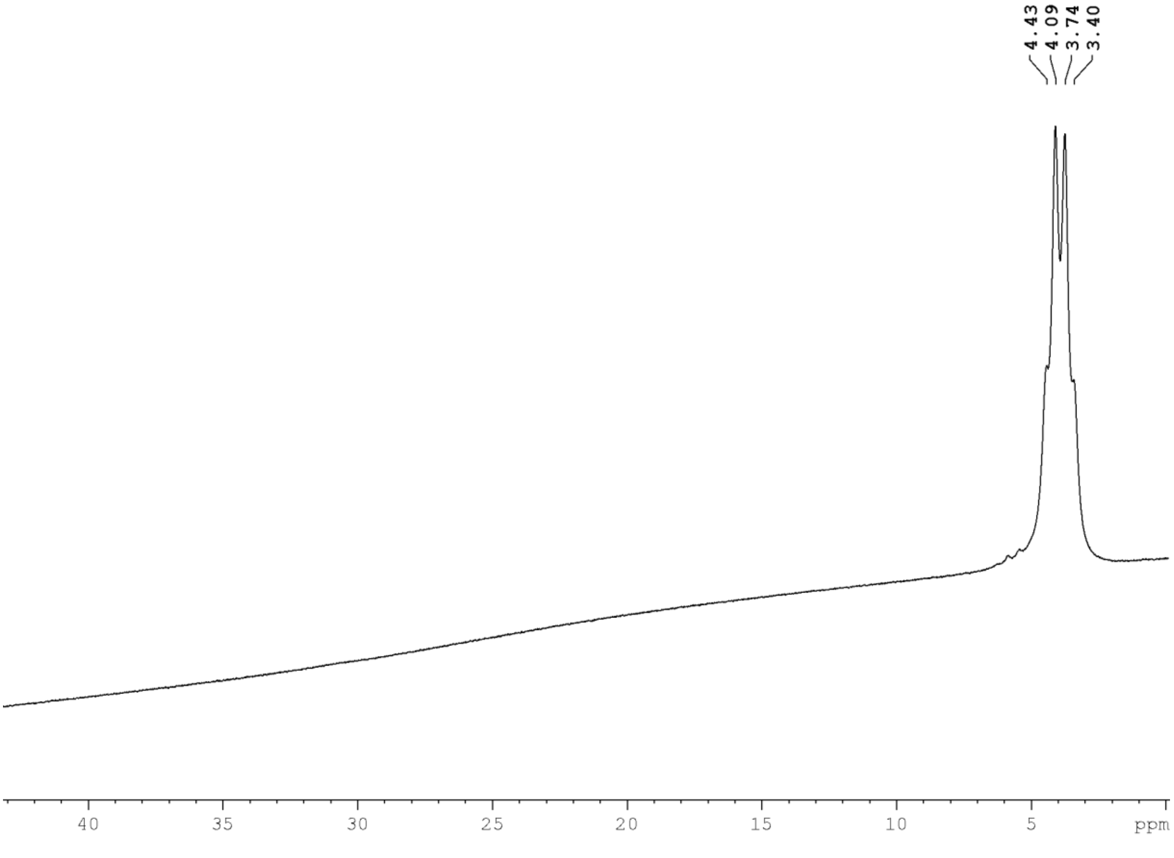

Figure S5. ^{11}B{^{1}H} NMR spectrum of **Ar^{Br}BF$_3$K** in acetone-d$_6$ at 160 MHz.

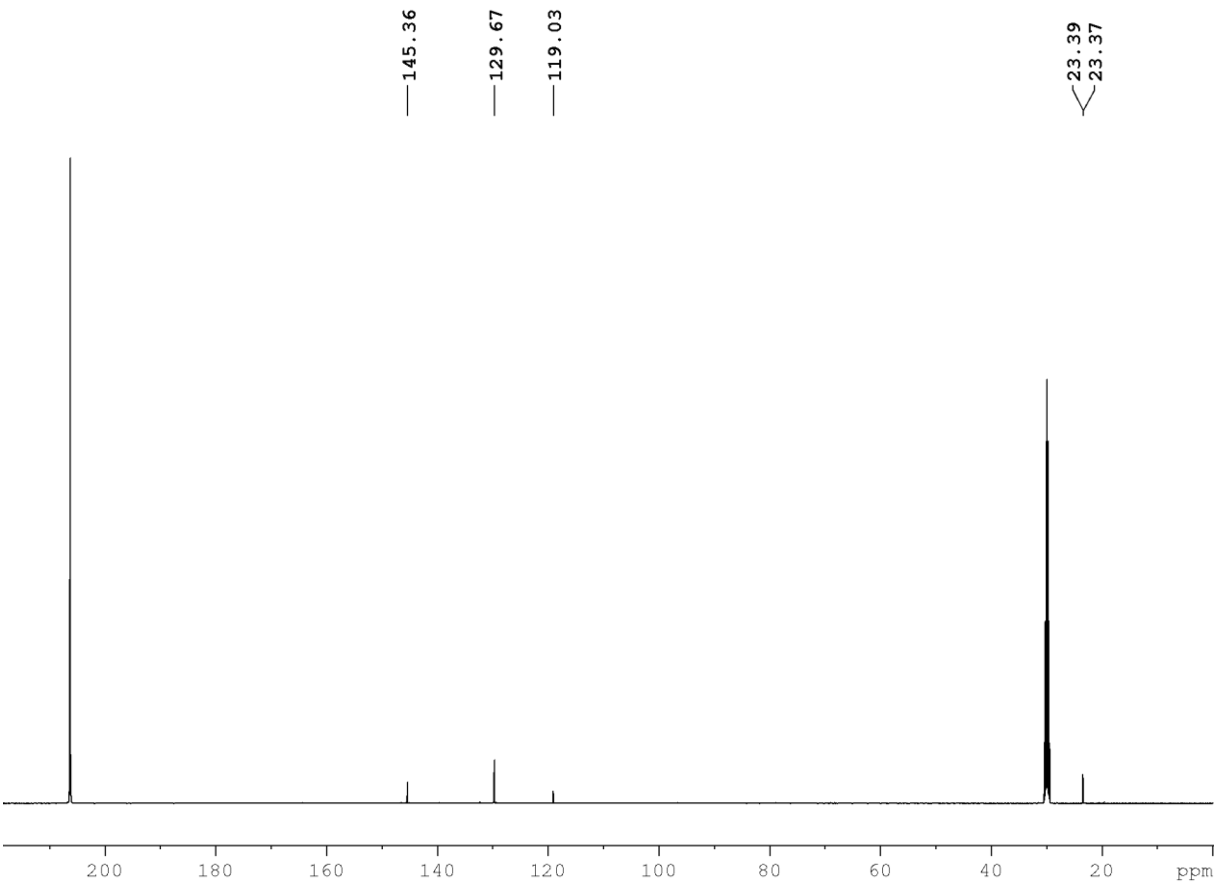

Figure S6. $^{13}C\{^{1}H\}$ NMR spectrum of $\mathbf{Ar^{Br}BF_3K}$ in acetone-d_6 at 125 MHz.

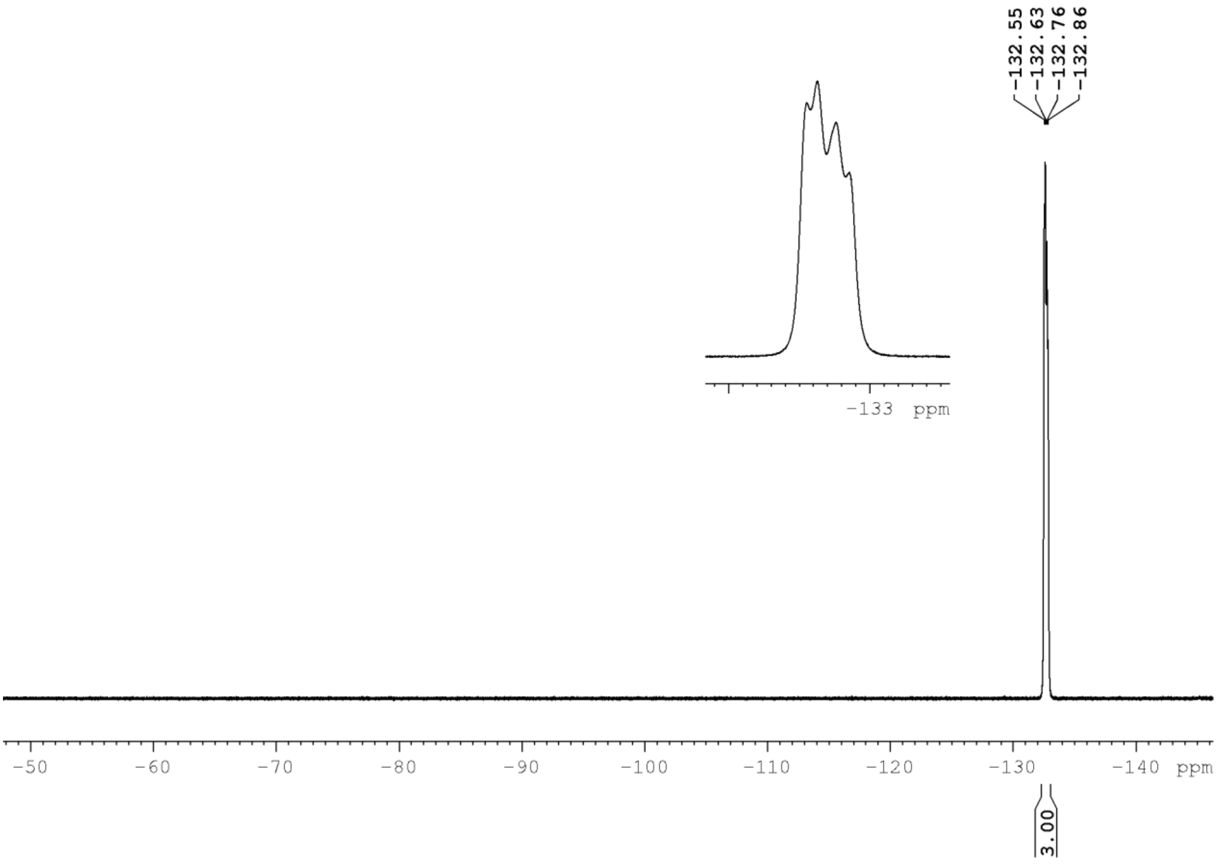

Figure S7. ^{19}F NMR spectrum of $\mathbf{Ar^{Br}BF_3K}$ in acetone-d_6 at 470 MHz.

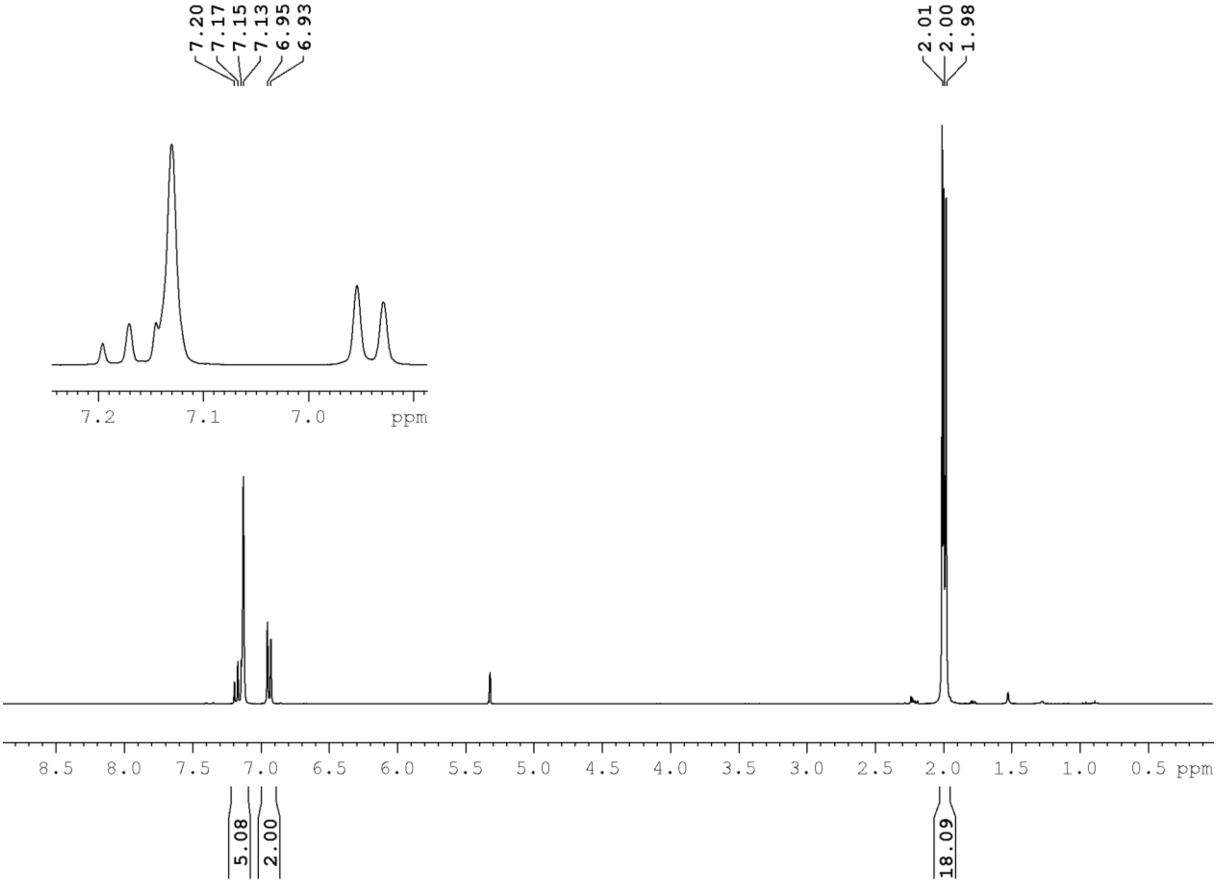

Figure S8. ^{1}H NMR spectrum of **$BAr^{H}Ar^{Br}Ar^{Br}$** in CD_2Cl_2 at 300 MHz.

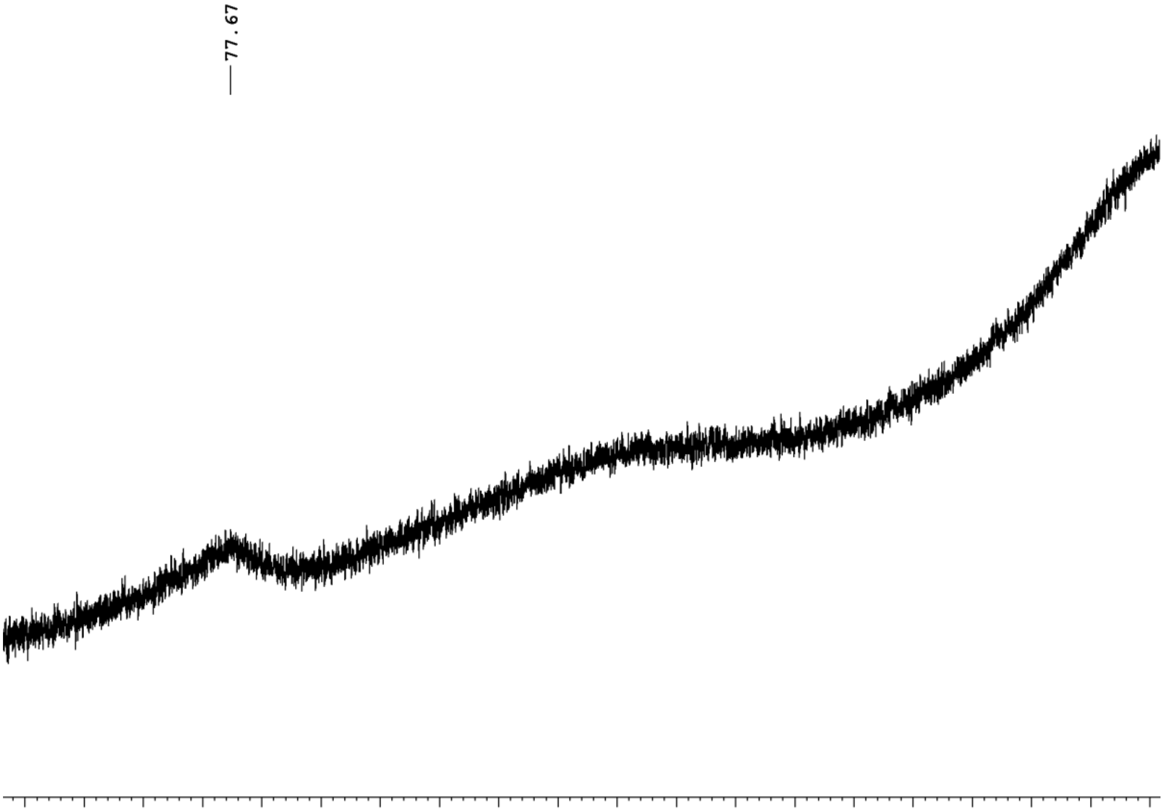

Figure S9. $^{11}B\{^{1}H\}$ NMR spectrum of **$BAr^{H}Ar^{Br}Ar^{Br}$** in CD_2Cl_2 at 96 MHz.

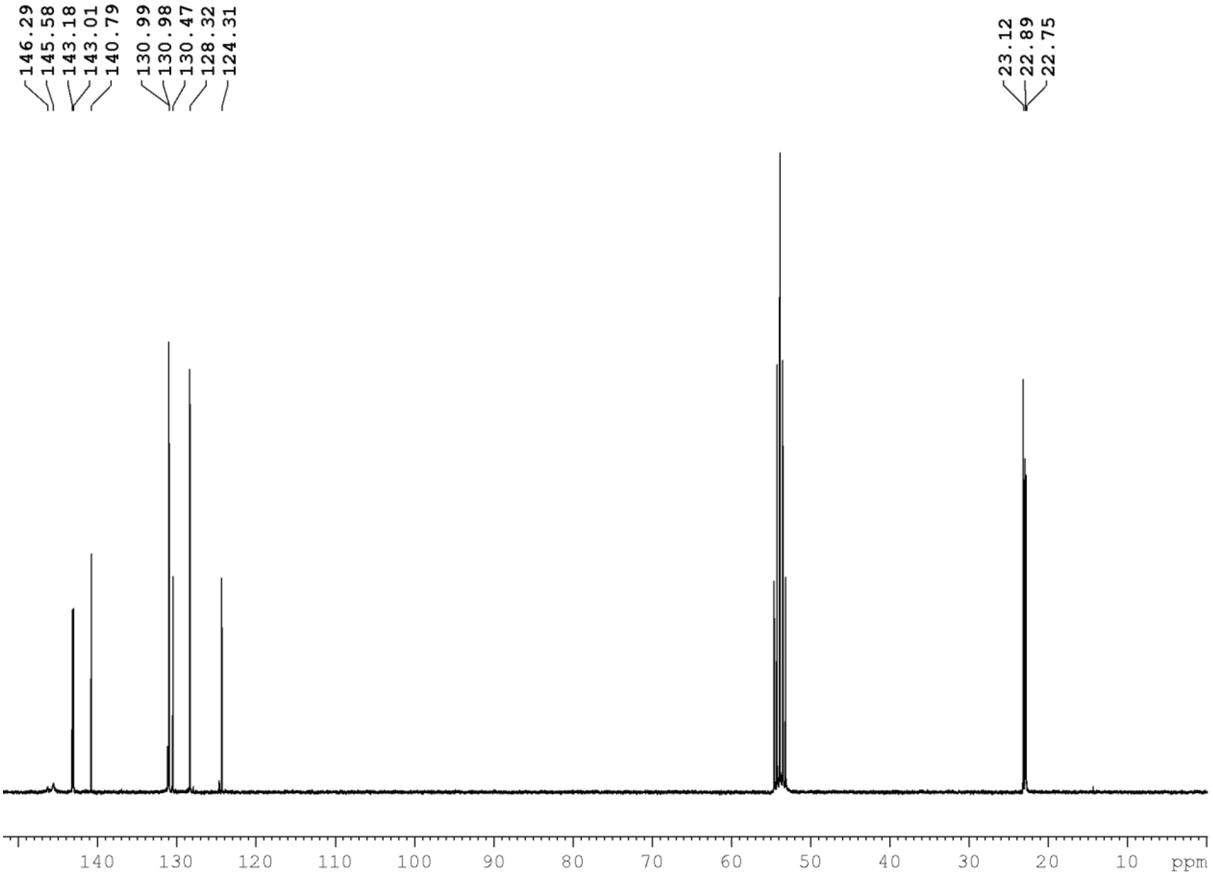

Figure S10. $^{13}C\{^1H\}$ NMR spectrum of **BAr^{H}Ar^{Br}Ar^{Br}** in CD_2Cl_2 at 75 MHz.

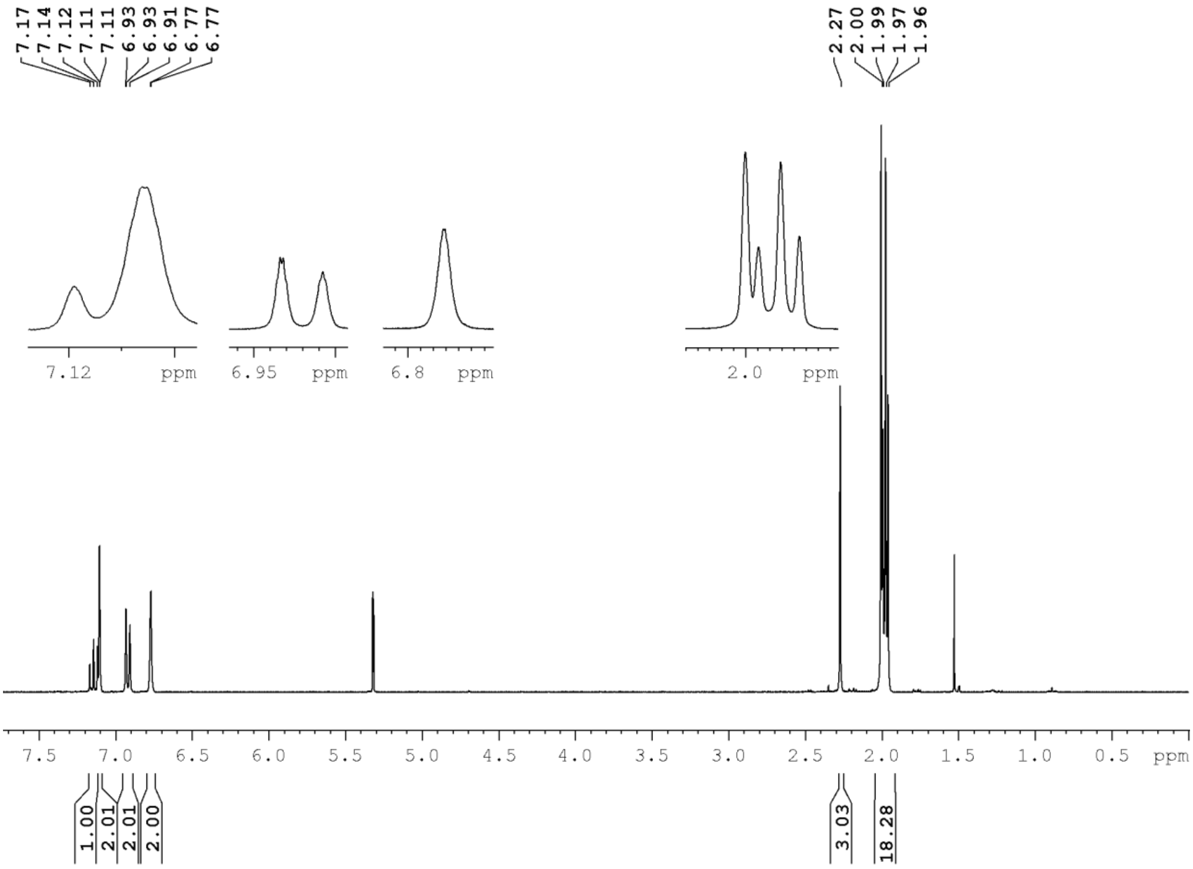

Figure S11. ^{1}H NMR spectrum of **BAr^{H}Ar^{Br}Ar^{Me}** in CD_2Cl_2 at 300 MHz.

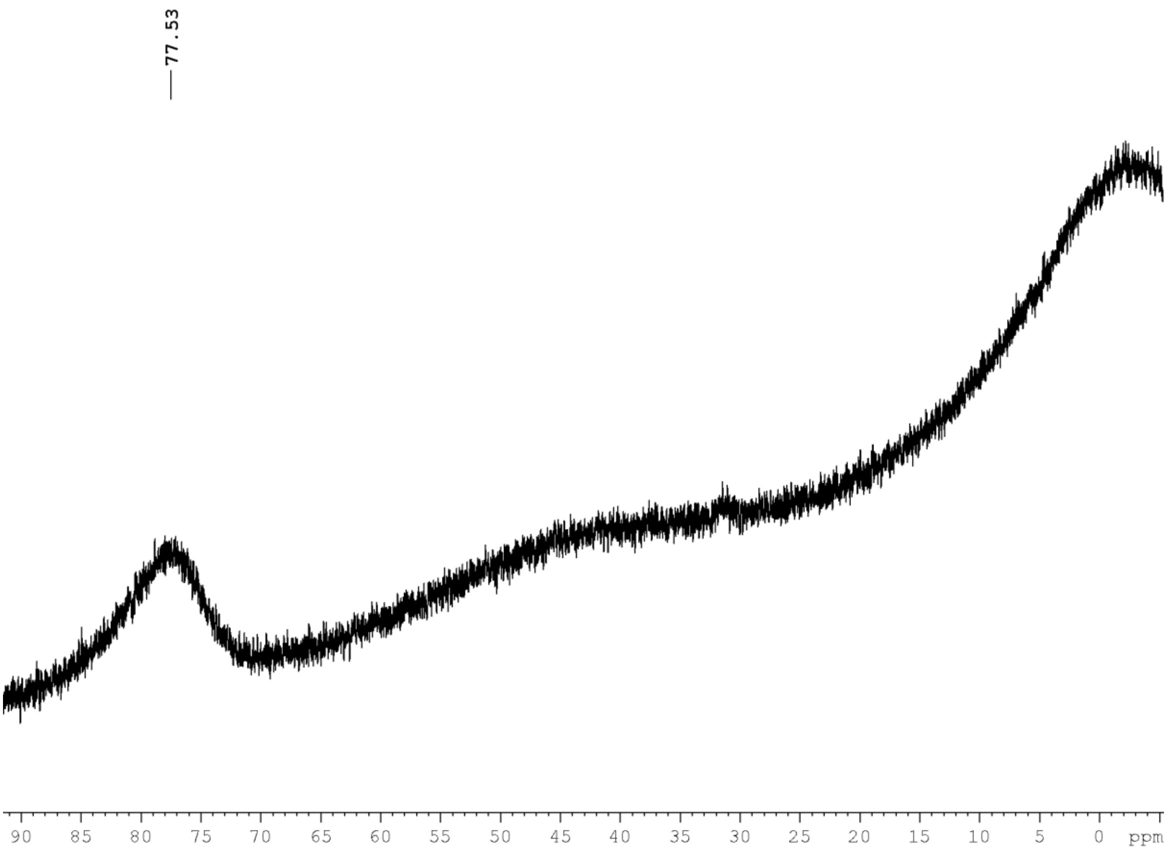

Figure S12. $^{11}B\{^{1}H\}$ NMR spectrum of **BAr^{H}Ar^{Br}Ar^{Me}** in CD_2Cl_2 at 96 MHz.

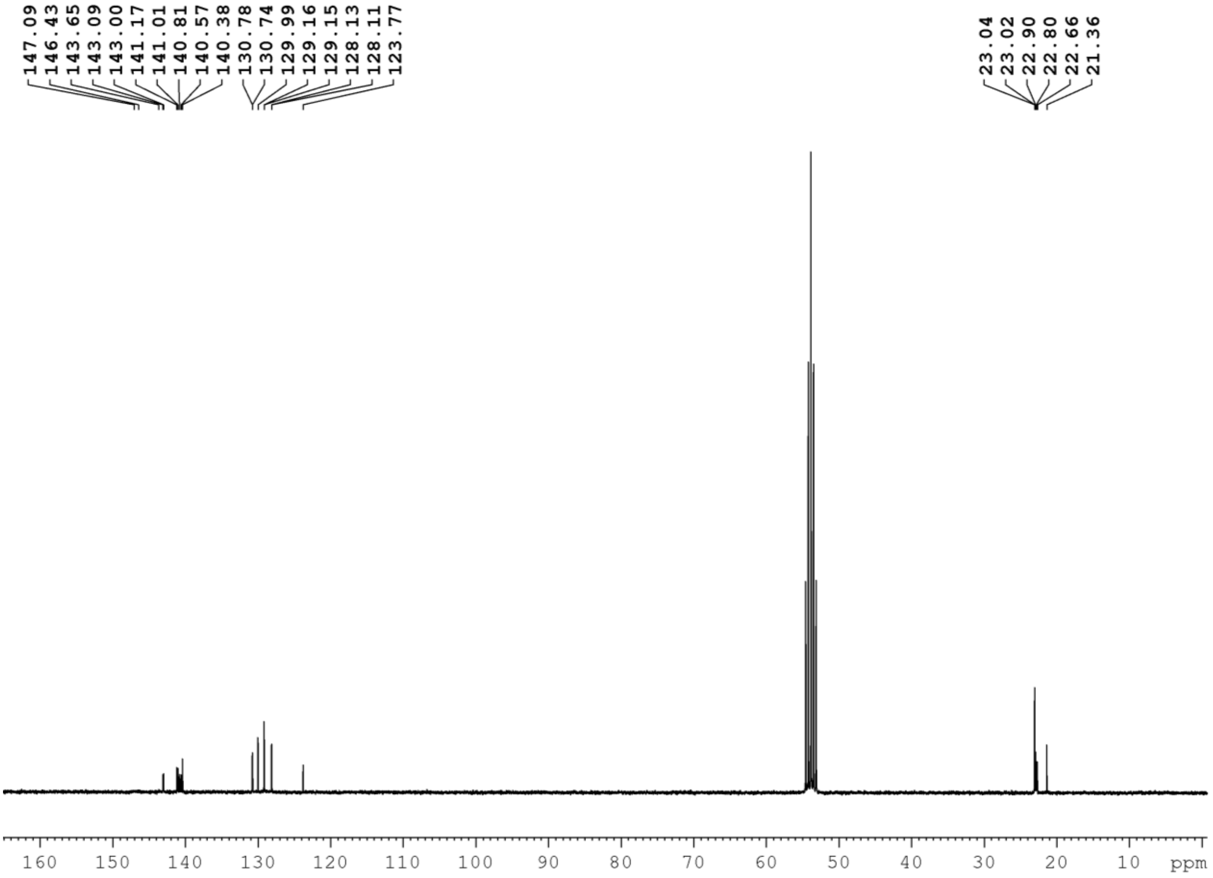

Figure S13. $^{13}C\{^{1}H\}$ NMR spectrum of **BAr^{H}Ar^{Br}Ar^{Me}** in CD_2Cl_2 at 75 MHz.

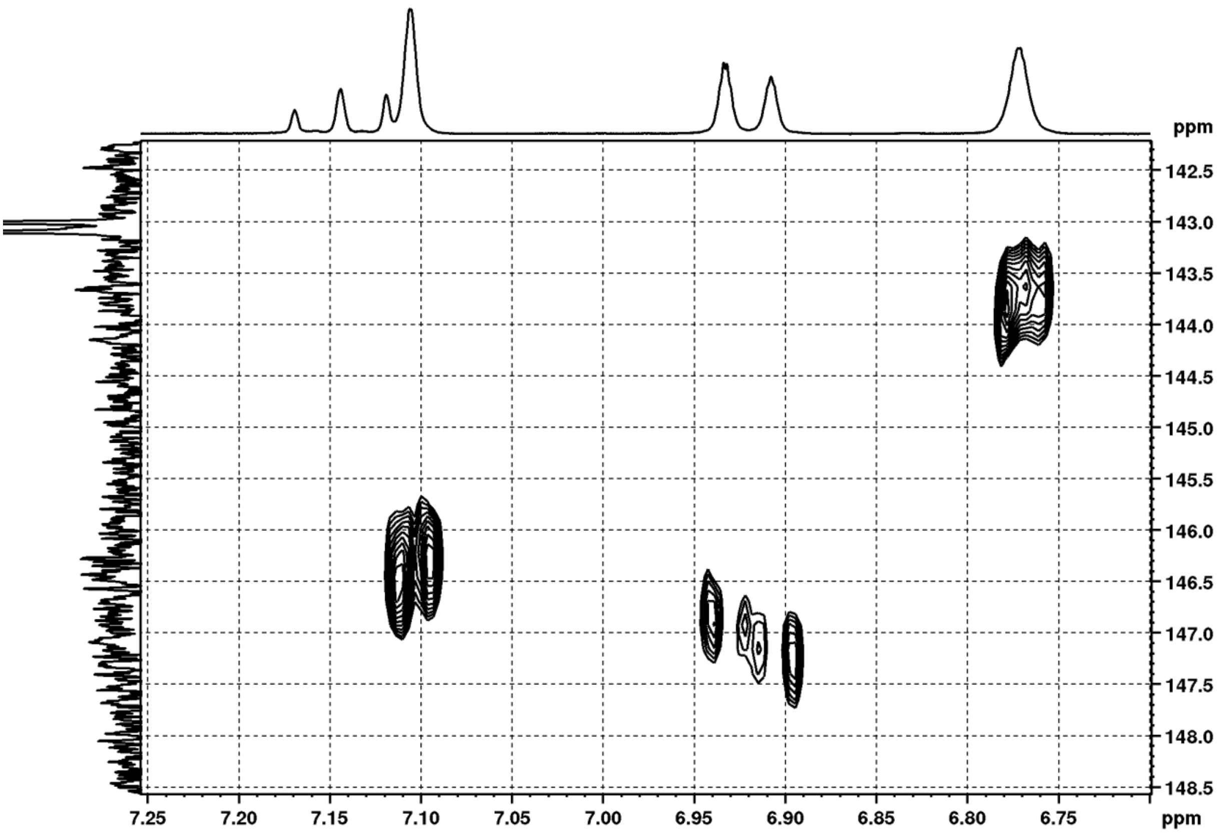

Figure S14. ^{1}H, ^{13}C HMBC NMR spectrum of **BAr^{H}Ar^{Br}Ar^{Me}** in CD_2Cl_2 at 300 MHz.

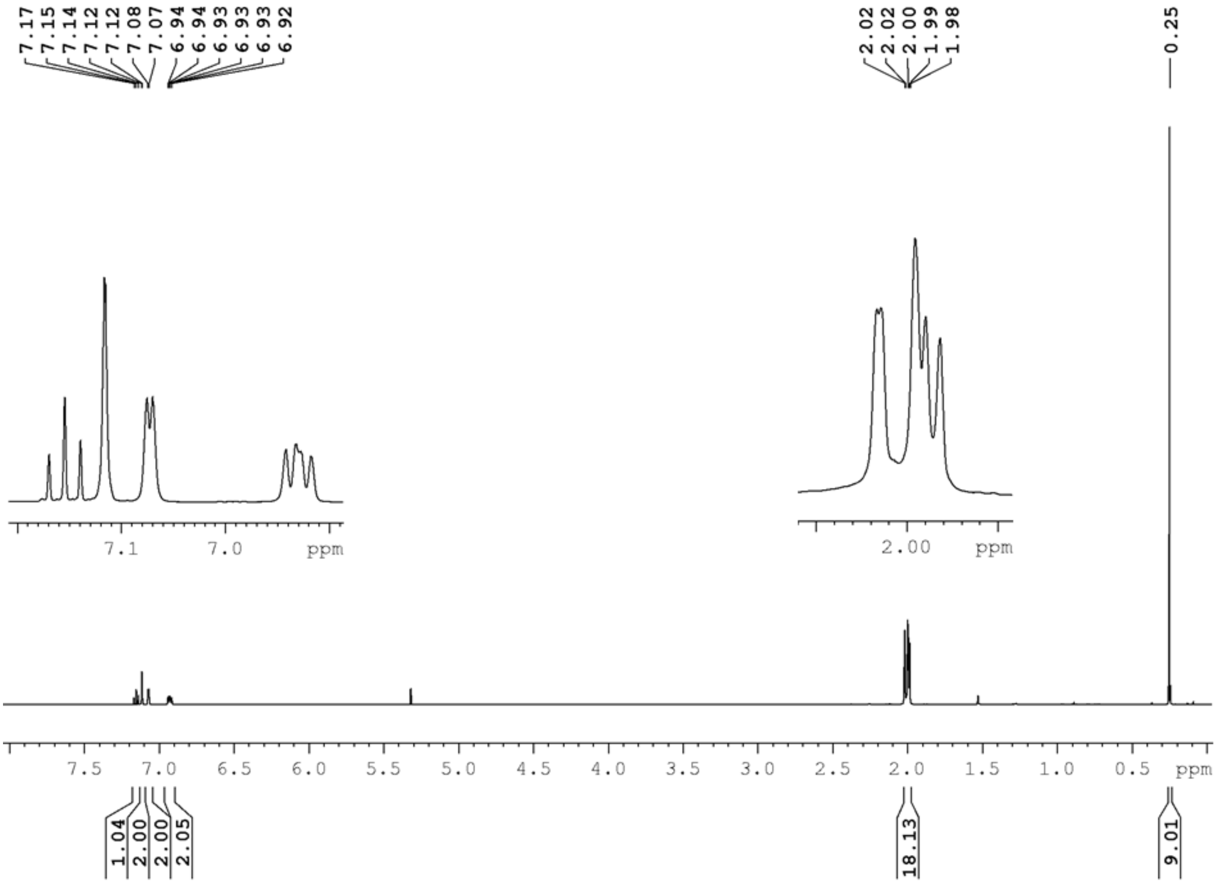

Figure S15. ^{1}H NMR spectrum of **BAr^{H}Ar^{Br}Ar^{SiMe_3}** in CD_2Cl_2 at 500 MHz.

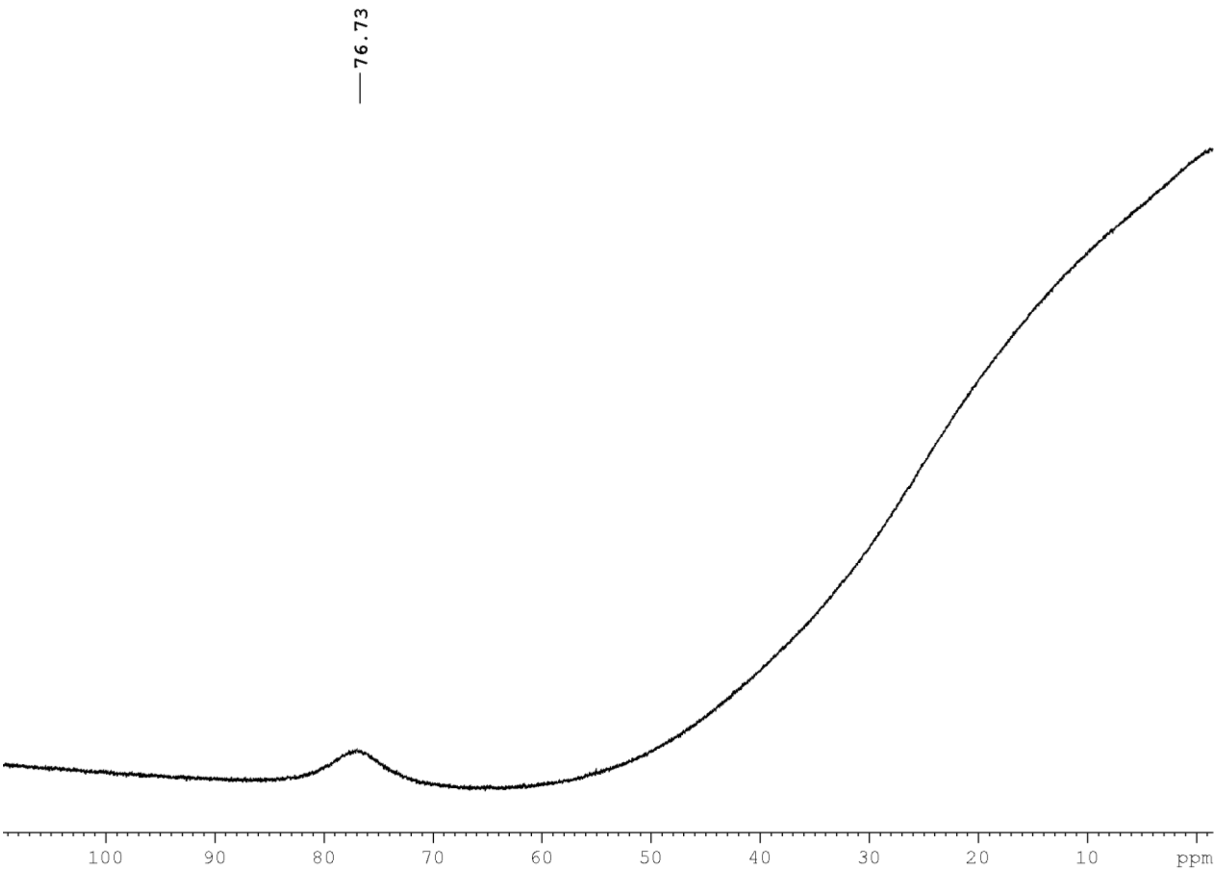

Figure S16. $^{11}B\{^{1}H\}$ NMR spectrum of $\mathbf{BAr^{H}Ar^{Br}Ar^{SiMe_3}}$ in CD_2Cl_2 at 160 MHz.

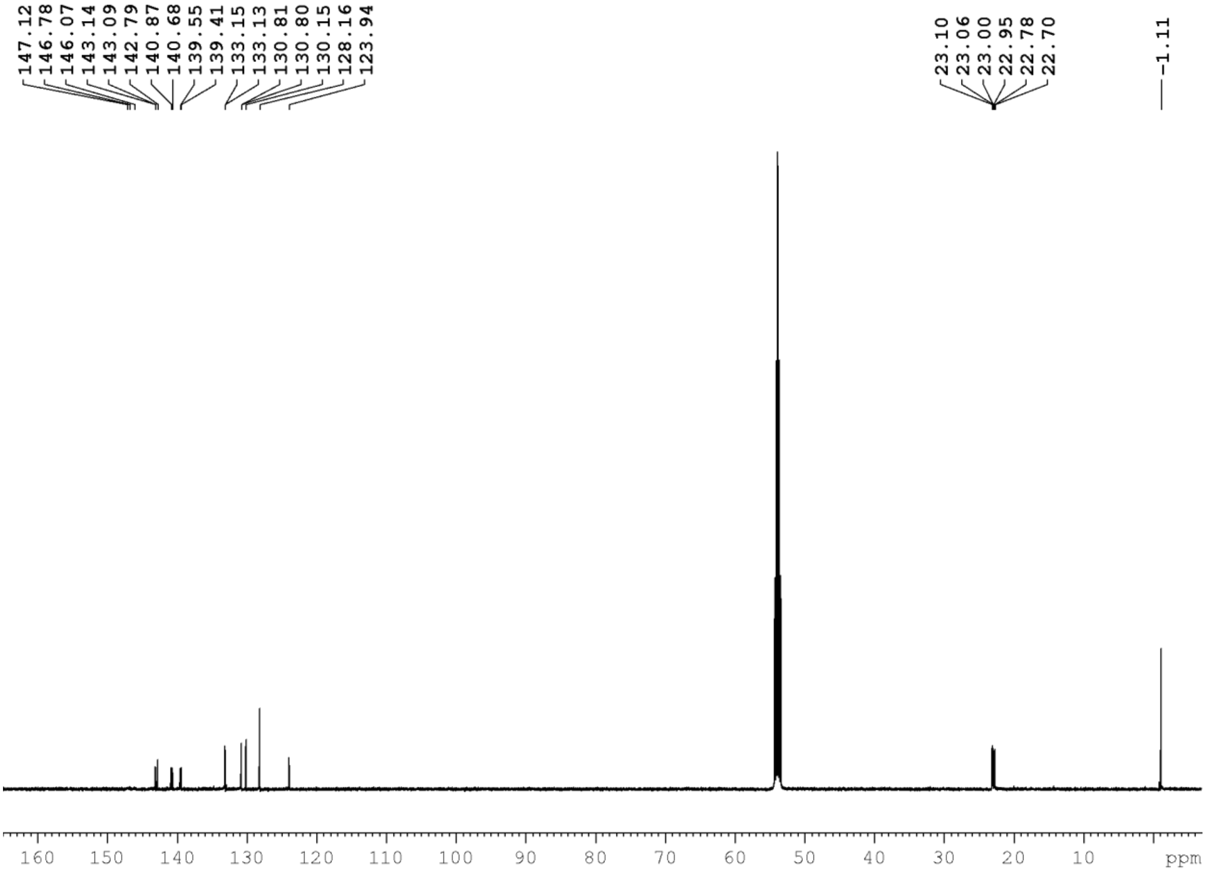

Figure S17. $^{13}C\{^{1}H\}$ NMR spectrum of $\mathbf{BAr^{H}Ar^{Br}Ar^{SiMe_3}}$ in CD_2Cl_2 at 125 MHz.

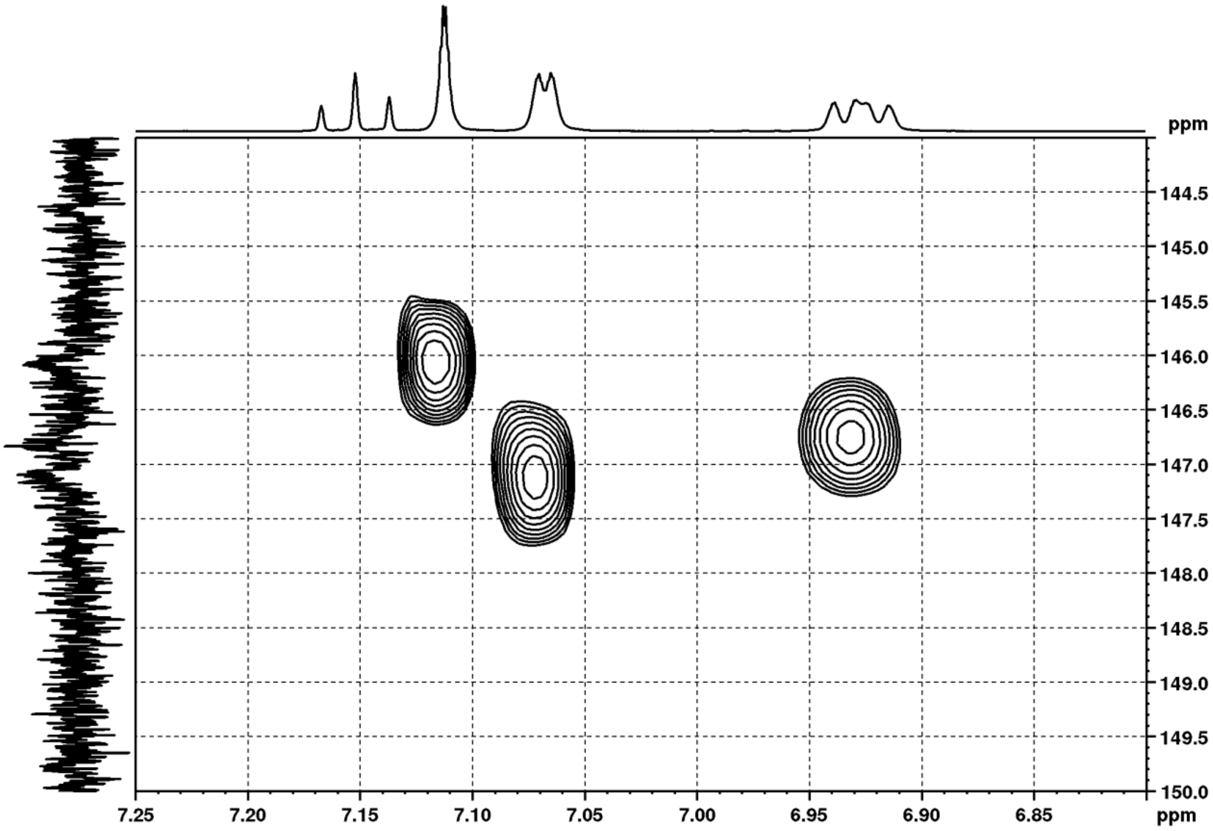

Figure S18. ^{1}H, ^{13}C HMBC NMR spectrum of **BAr^{H}Ar^{Br}Ar^{SiMe_3}** in CD_2Cl_2 at 500 MHz.

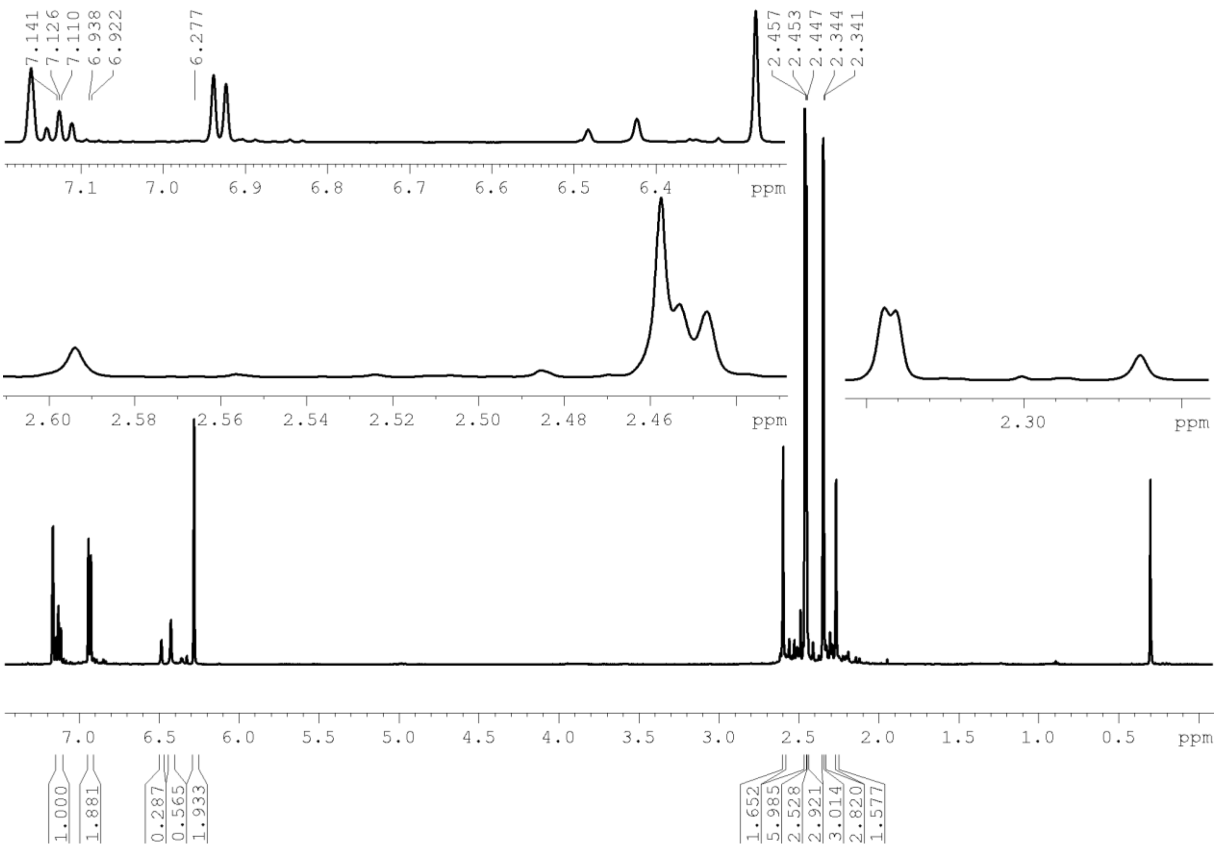

Figure S19. ^{1}H NMR spectrum of **BFAr^{H}Ar^{NMe_2}** in C_6D_6 at 500 MHz.

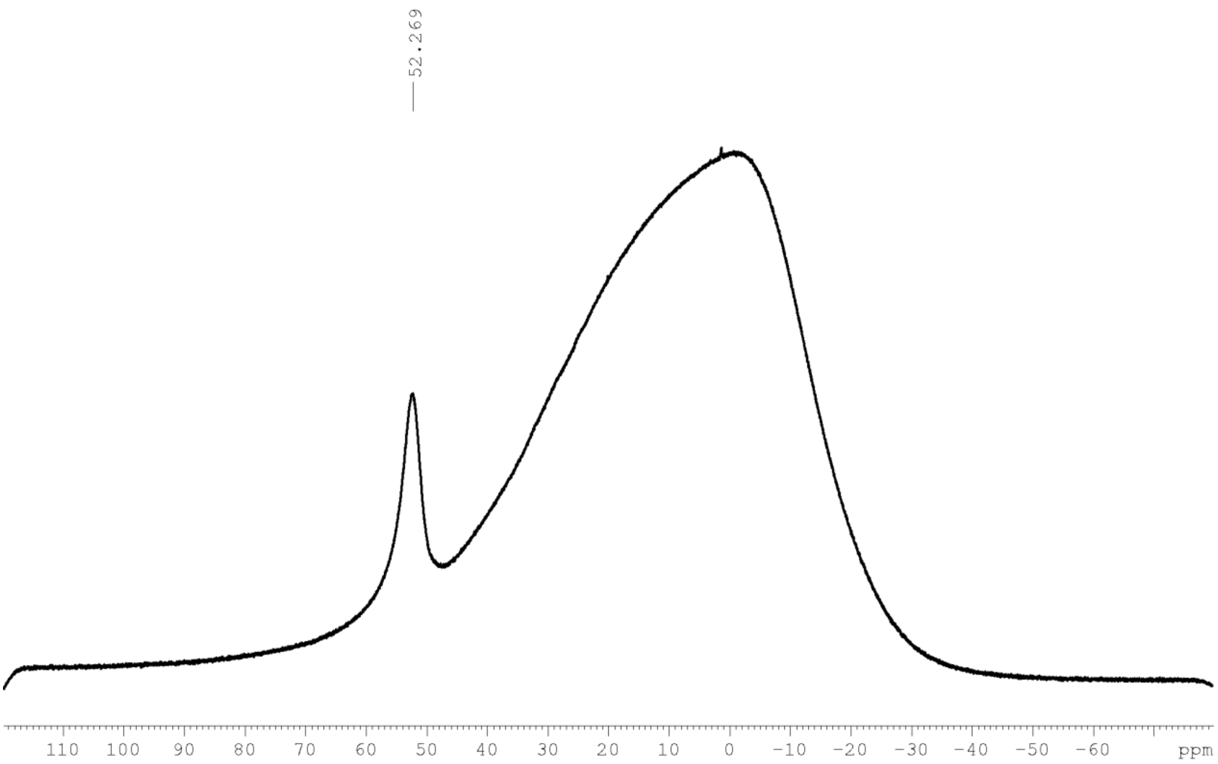

Figure S20. $^{11}B\{^{1}H\}$ NMR spectrum of **BFAr^{H}Ar^{NMe_2}** in C_6D_6 at 160 MHz.

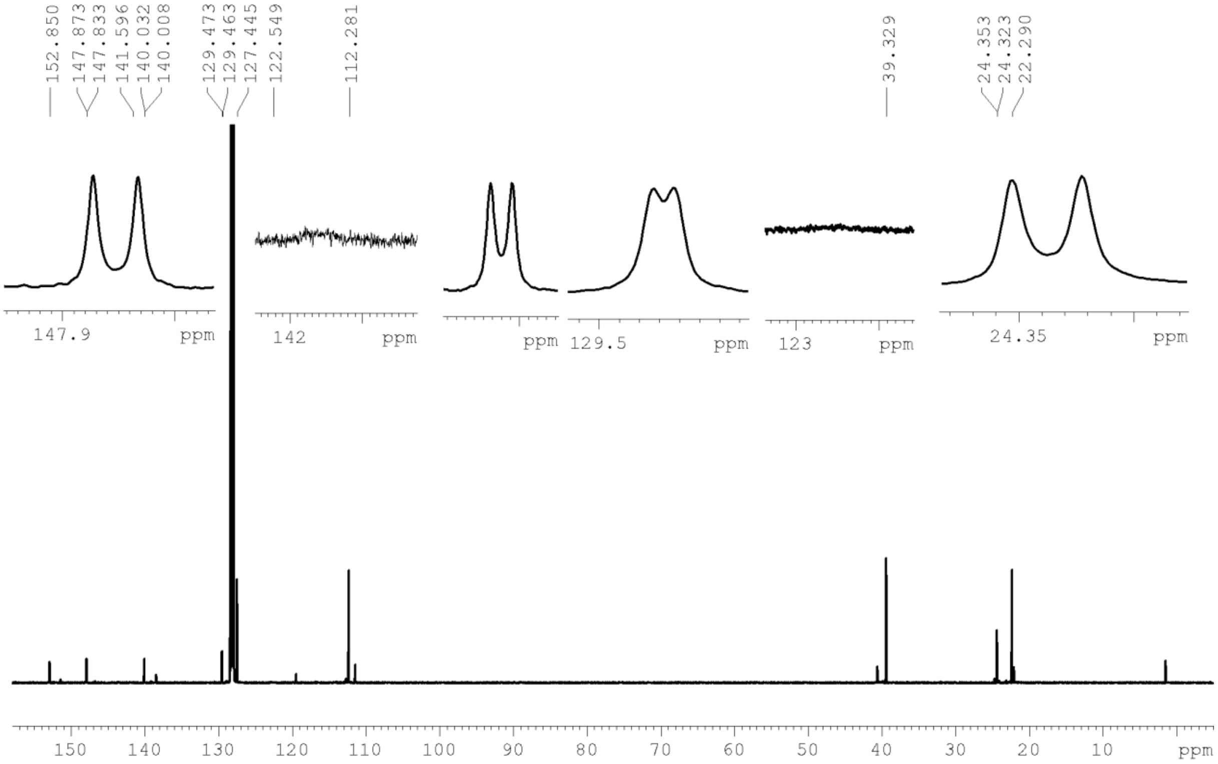

Figure S21. $^{13}C\{^{1}H\}$ NMR spectrum of **BFAr^{H}Ar^{NMe_2}** in C_6D_6 at 125 MHz.

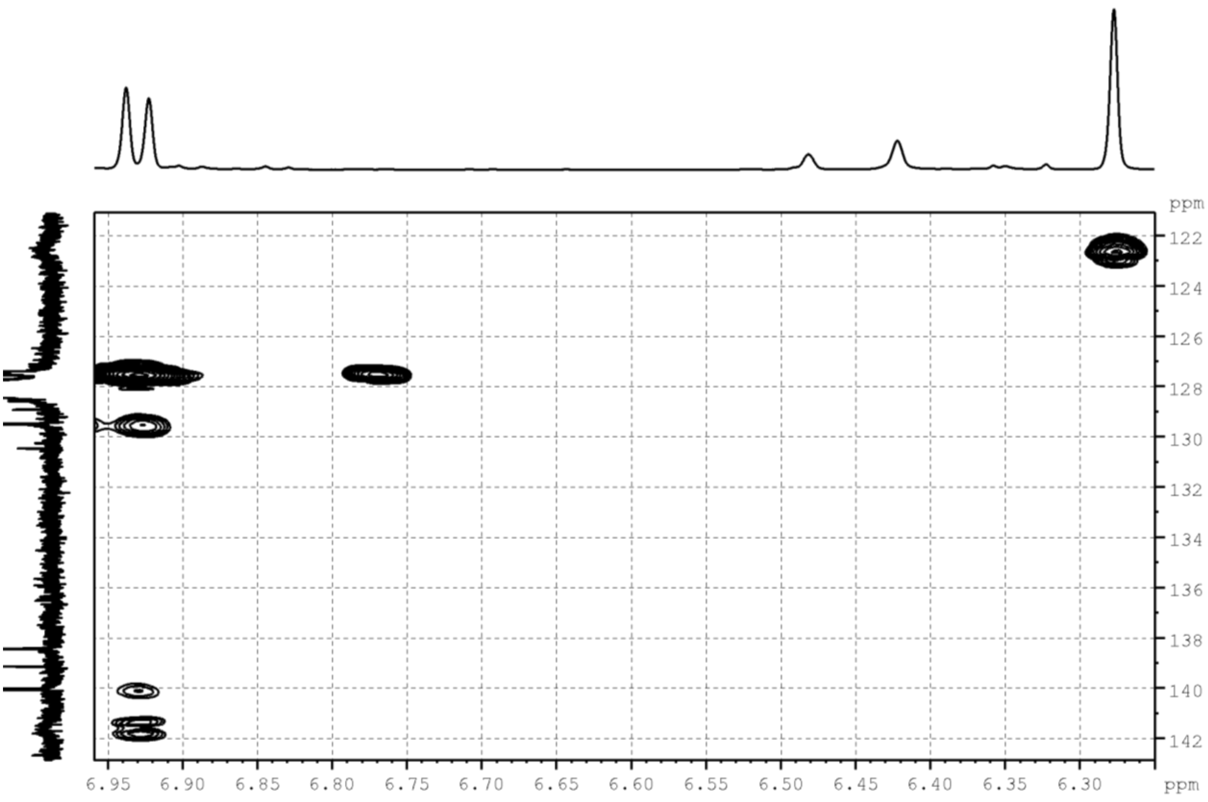

Figure S22. ^{1}H, ^{13}C HMBC NMR spectrum of **BFAr^{H}Ar^{NMe_2}** in C_6D_6 at 500 MHz.

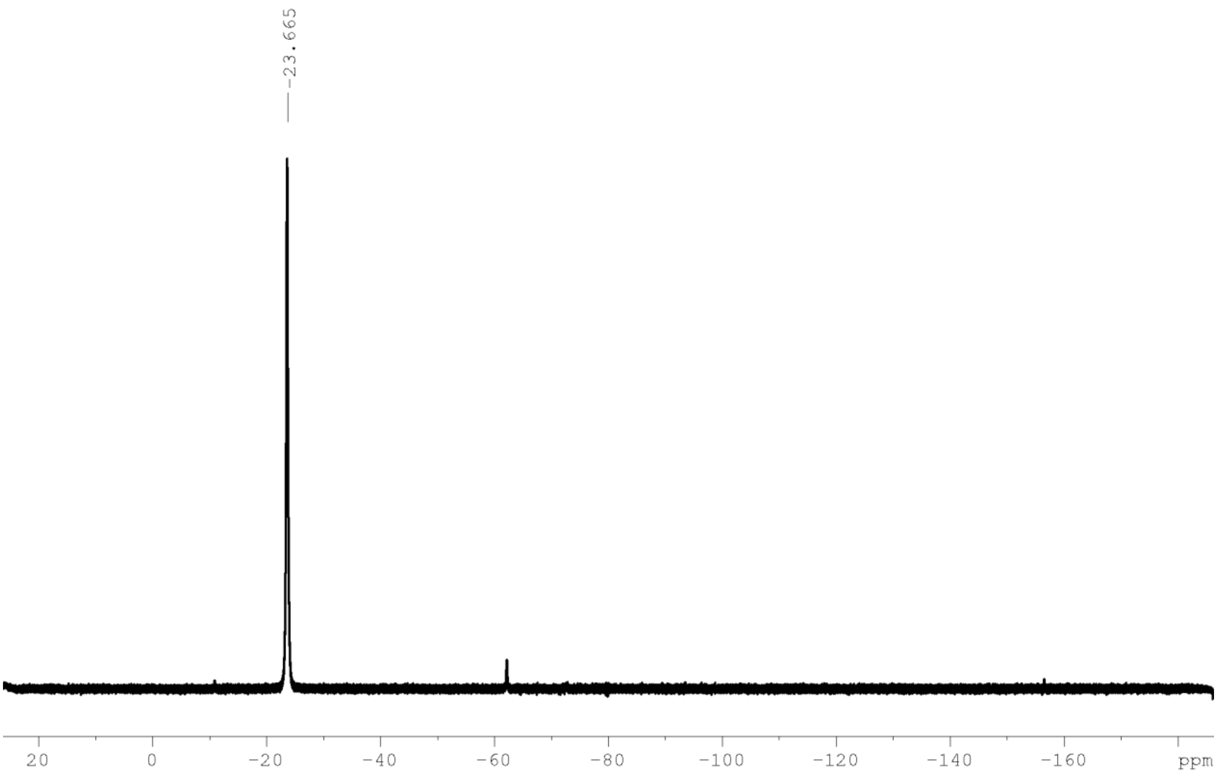

Figure S23. ^{19}F NMR spectrum of **BFAr^{H}Ar^{NMe_2}** in C_6D_6 at 470 MHz.

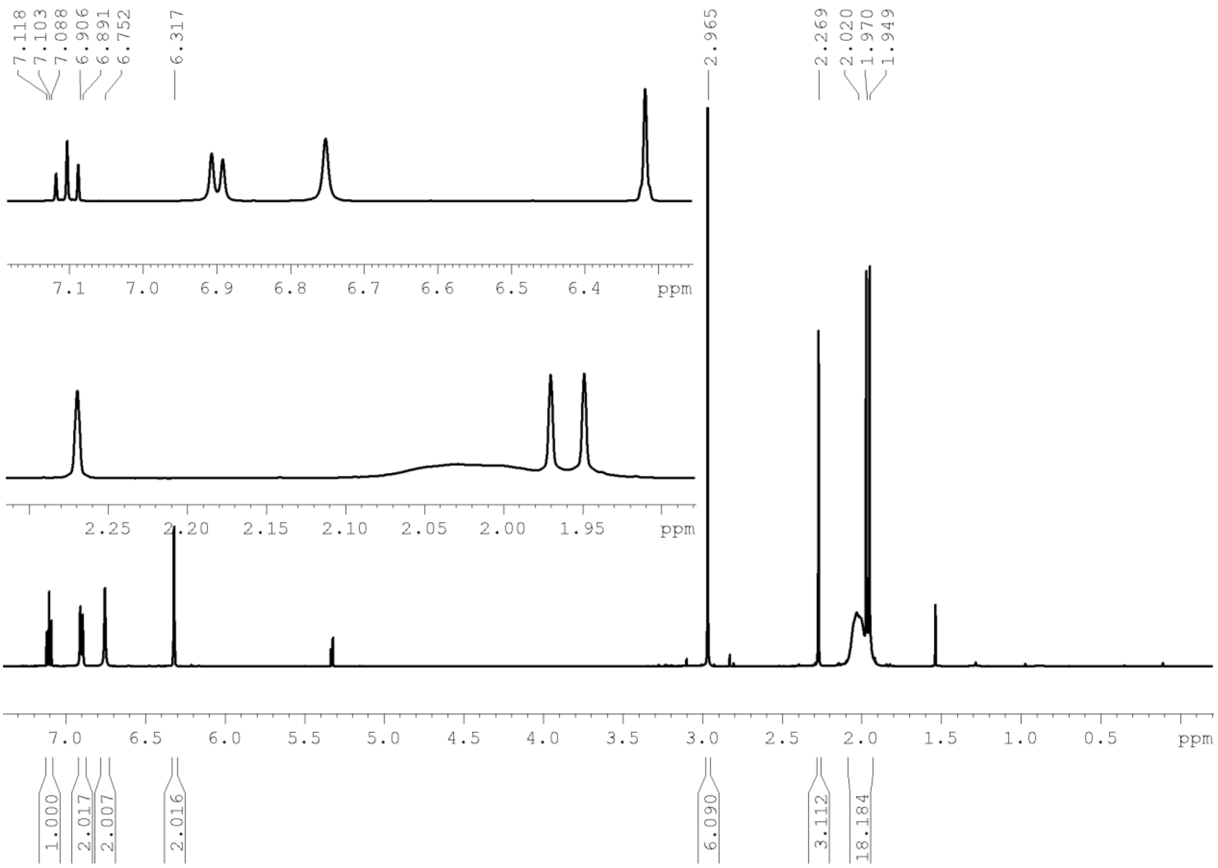

Figure S24. ^{1}H NMR spectrum of $\mathbf{BAr^{H}Ar^{Me}Ar^{NMe_2}}$ in CD_2Cl_2 at 500 MHz.

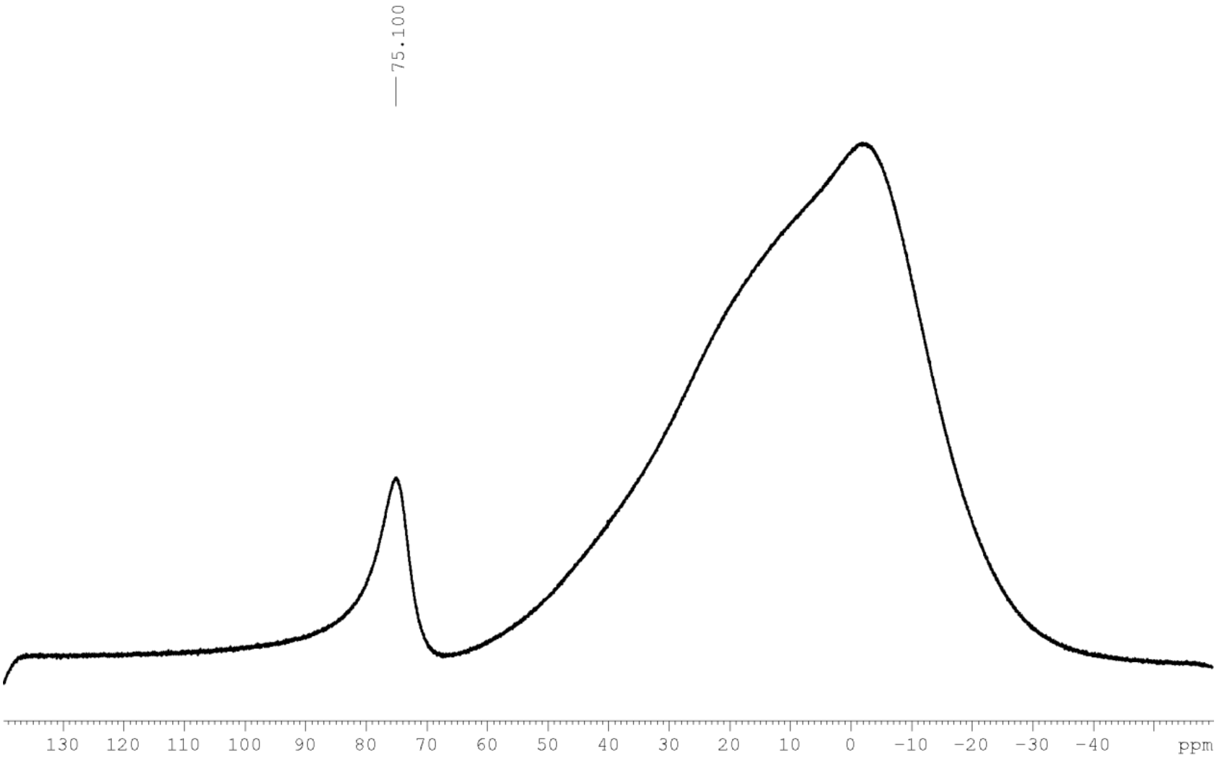

Figure S25. ^{11}B{^{1}H} NMR spectrum of $\mathbf{BAr^{H}Ar^{Me}Ar^{NMe_2}}$ in CD_2Cl_2 at 160 MHz.

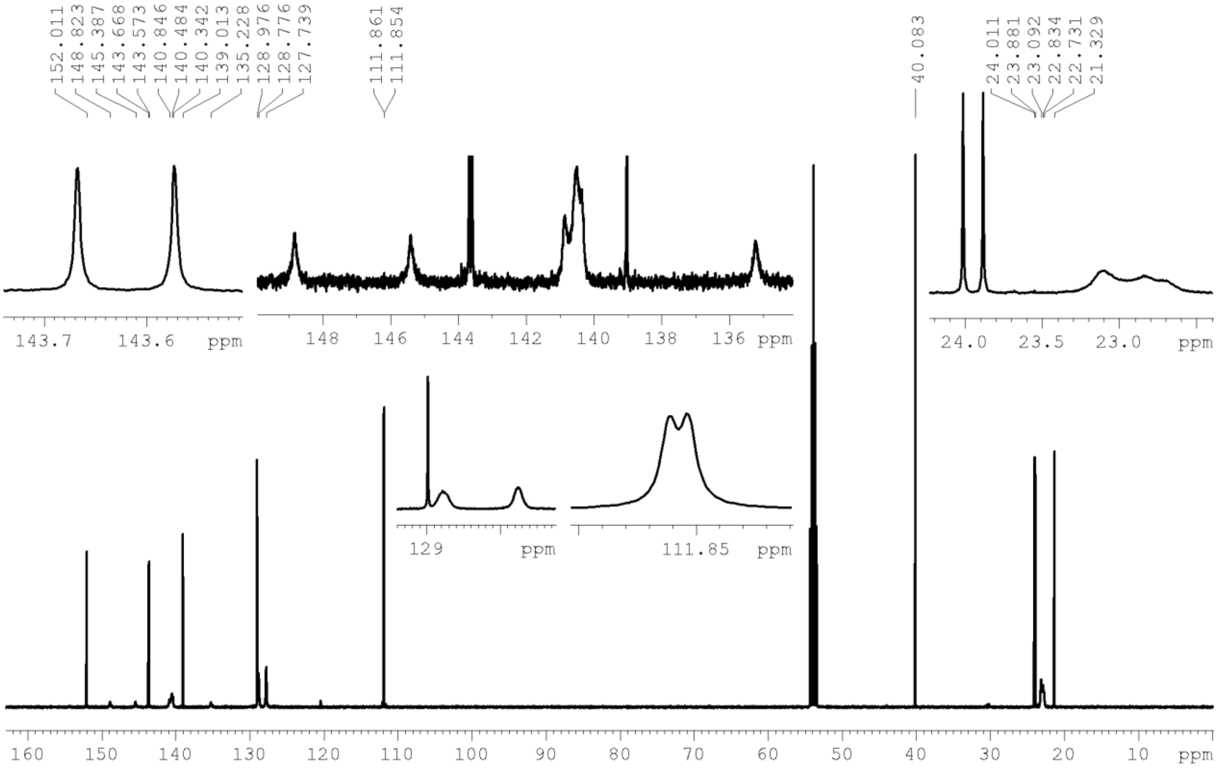

Figure S26. $^{13}C\{^{1}H\}$ NMR spectrum of **BAr^{H}Ar^{Me}Ar^{NMe_2}** in CD_2Cl_2 at 125 MHz.

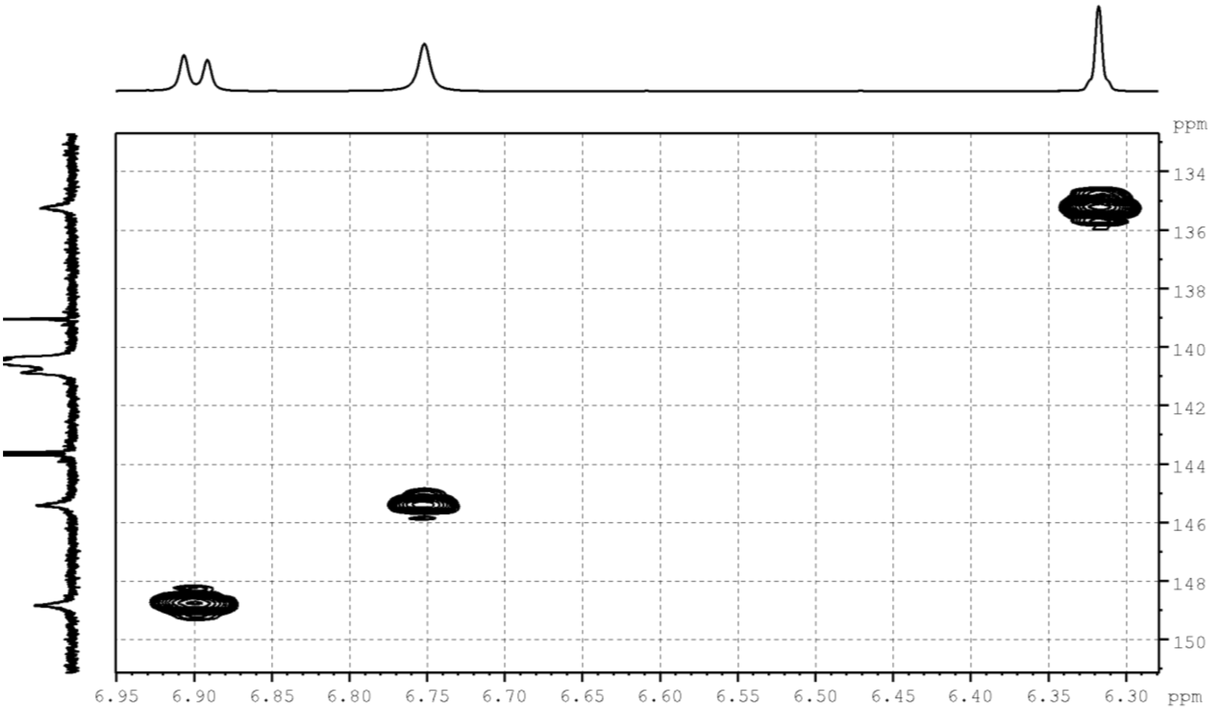

Figure S27. ^{1}H, ^{13}C HMBC NMR spectrum of **BAr^{H}Ar^{Me}Ar^{NMe_2}** in CD_2Cl_2 at 500 MHz.

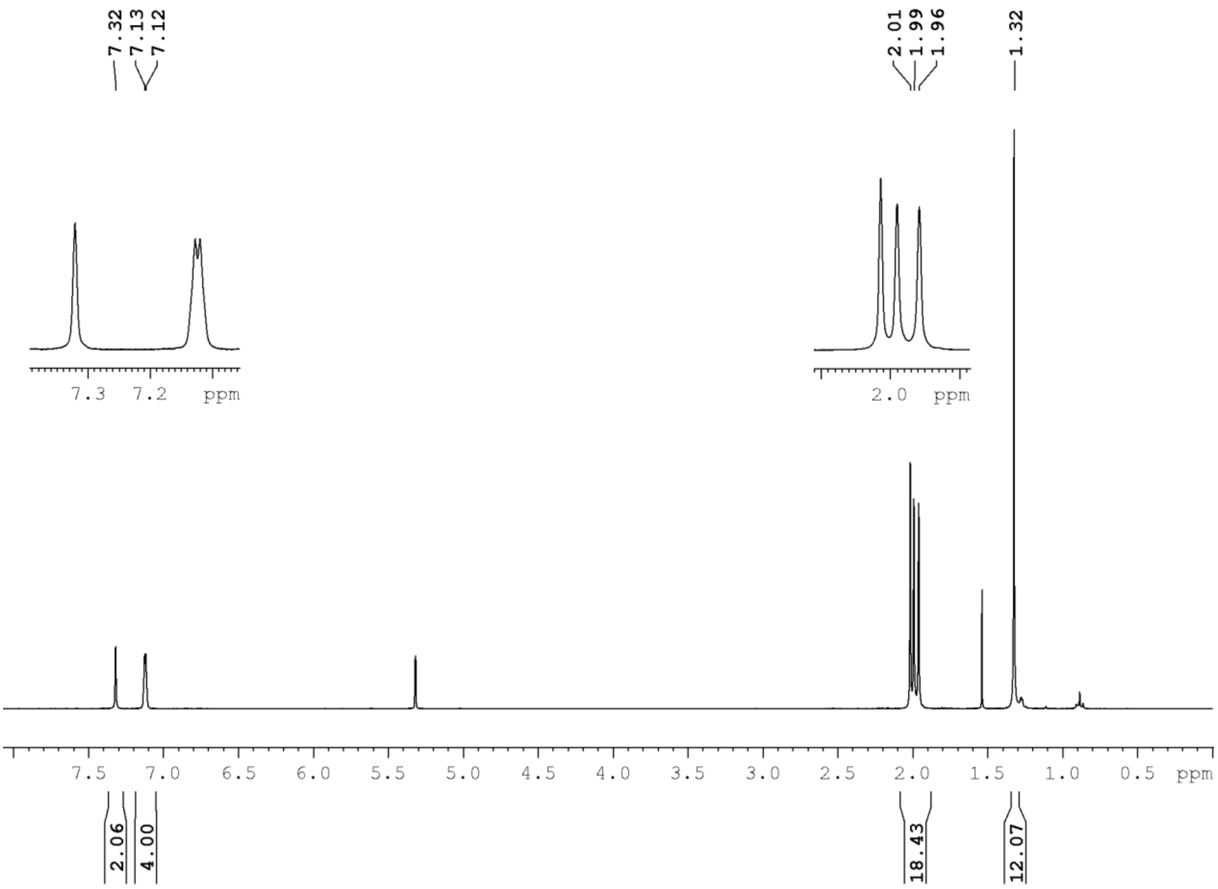

Figure S28. ^{1}H NMR spectrum of **BAr^{Bpin}Ar^{Br}Ar^{Br}** in CD_2Cl_2 at 300 MHz.

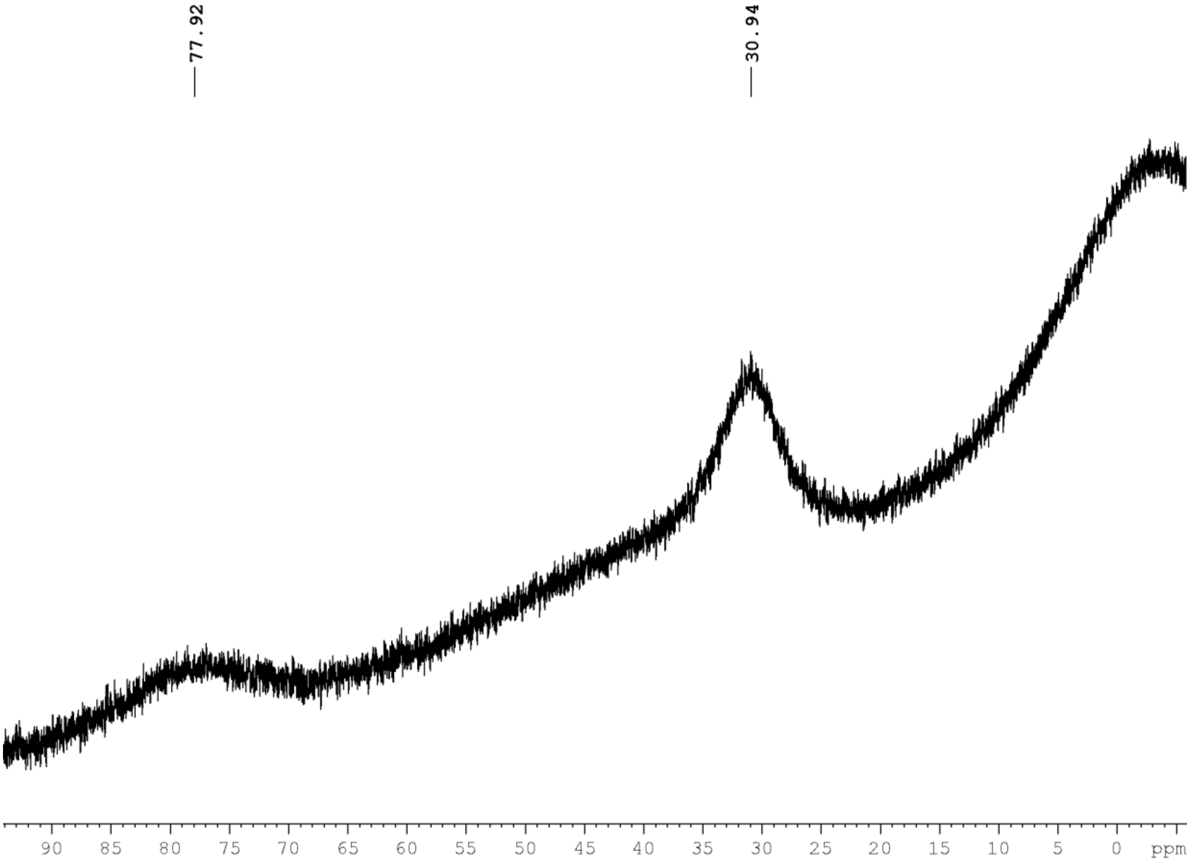

Figure S29. $^{11}B\{^{1}H\}$ NMR spectrum of **BAr^{Bpin}Ar^{Br}Ar^{Br}** in CD_2Cl_2 at 96 MHz.

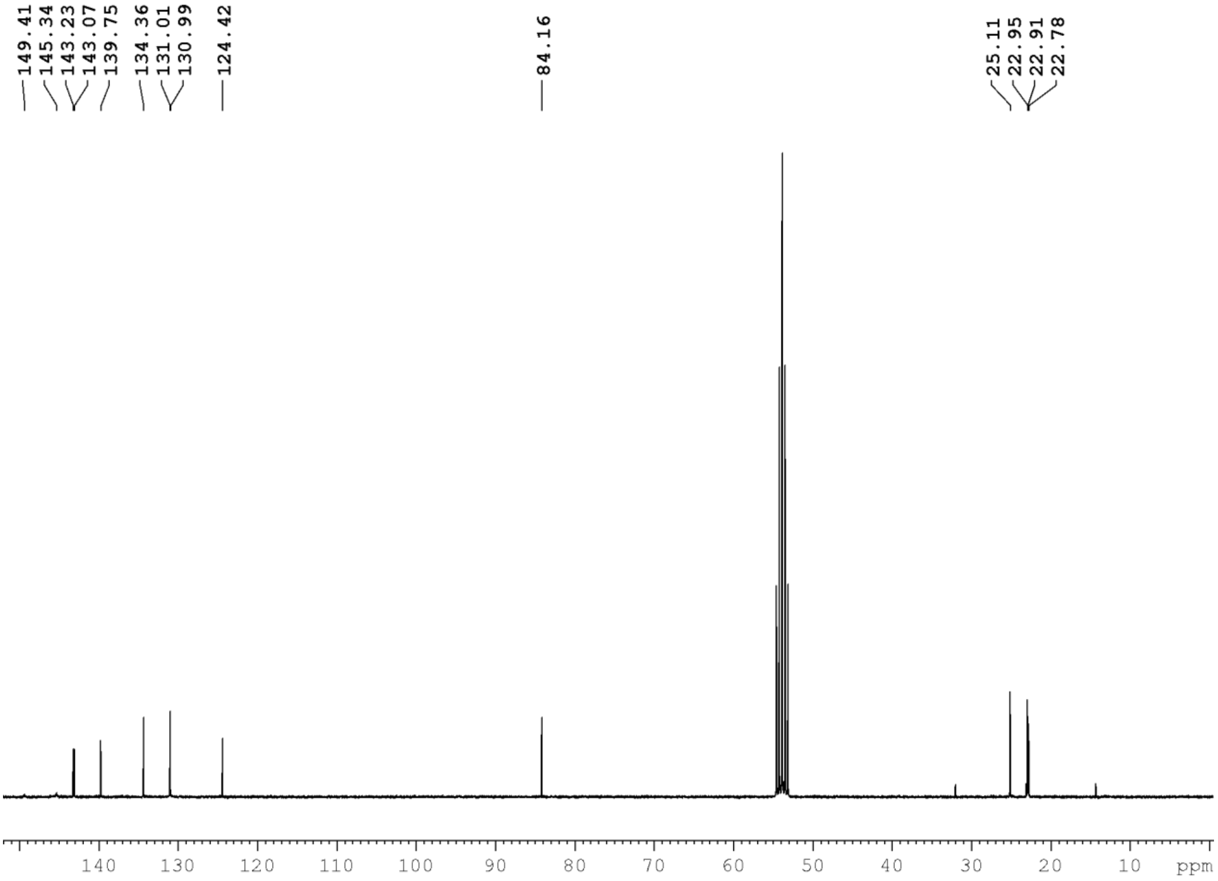

Figure S30. $^{13}C\{^{1}H\}$ NMR spectrum of **BAr^{Bpin}Ar^{Br}Ar^{Br}** in CD_2Cl_2 at 75 MHz.

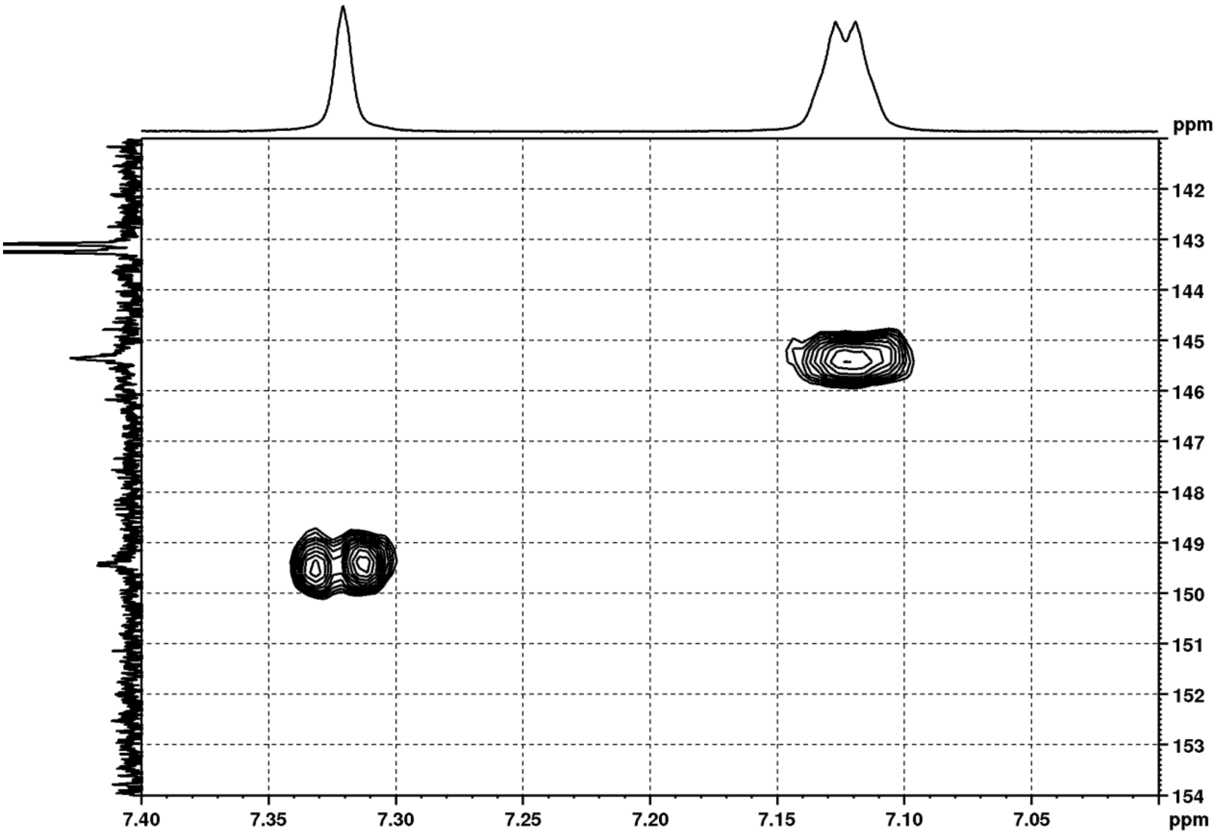

Figure S31. ^{1}H, ^{13}C HMBC NMR spectrum of **BAr^{Bpin}Ar^{Br}Ar^{Br}** in CD_2Cl_2 at 300 MHz.

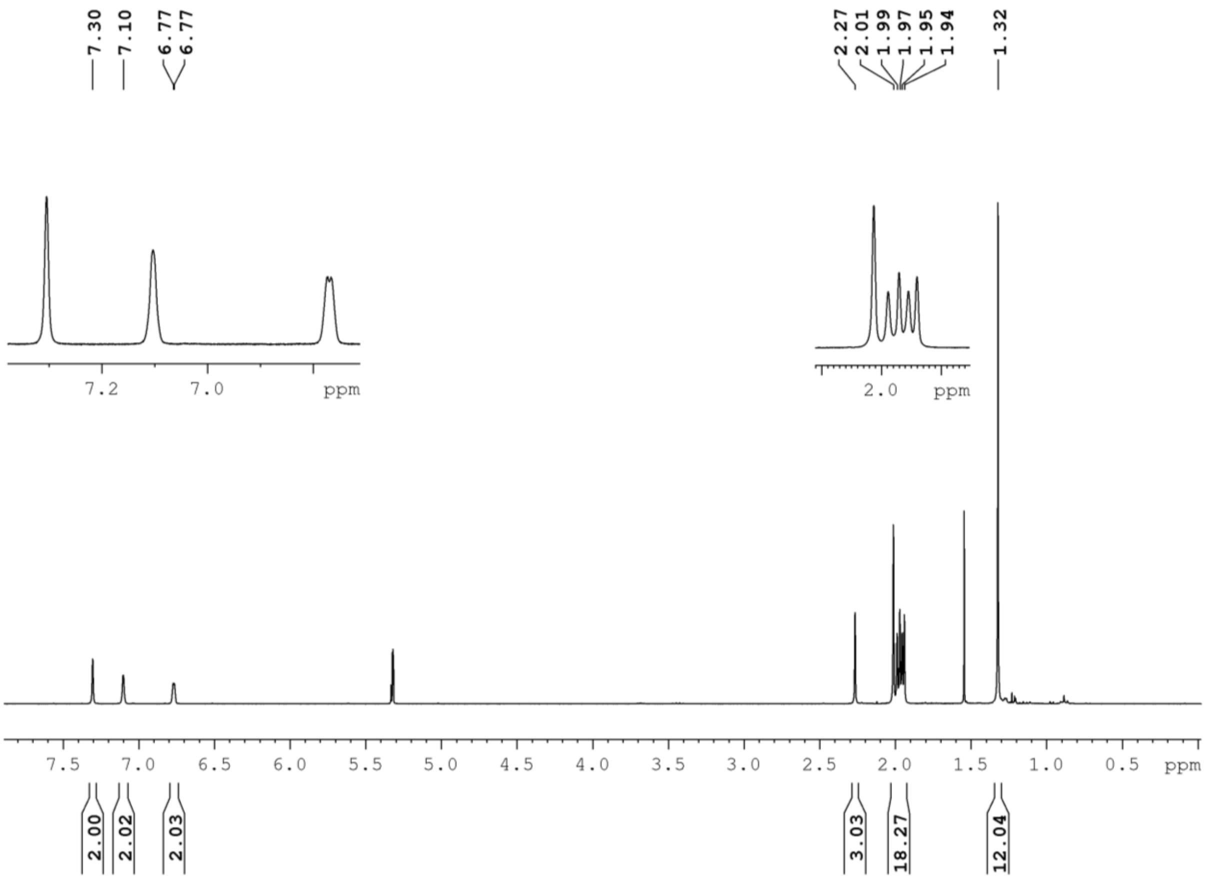

Figure S32. ^{1}H NMR spectrum of **BAr^{Bpin}Ar^{Br}Ar^{Me}** in CD_2Cl_2 at 300 MHz.

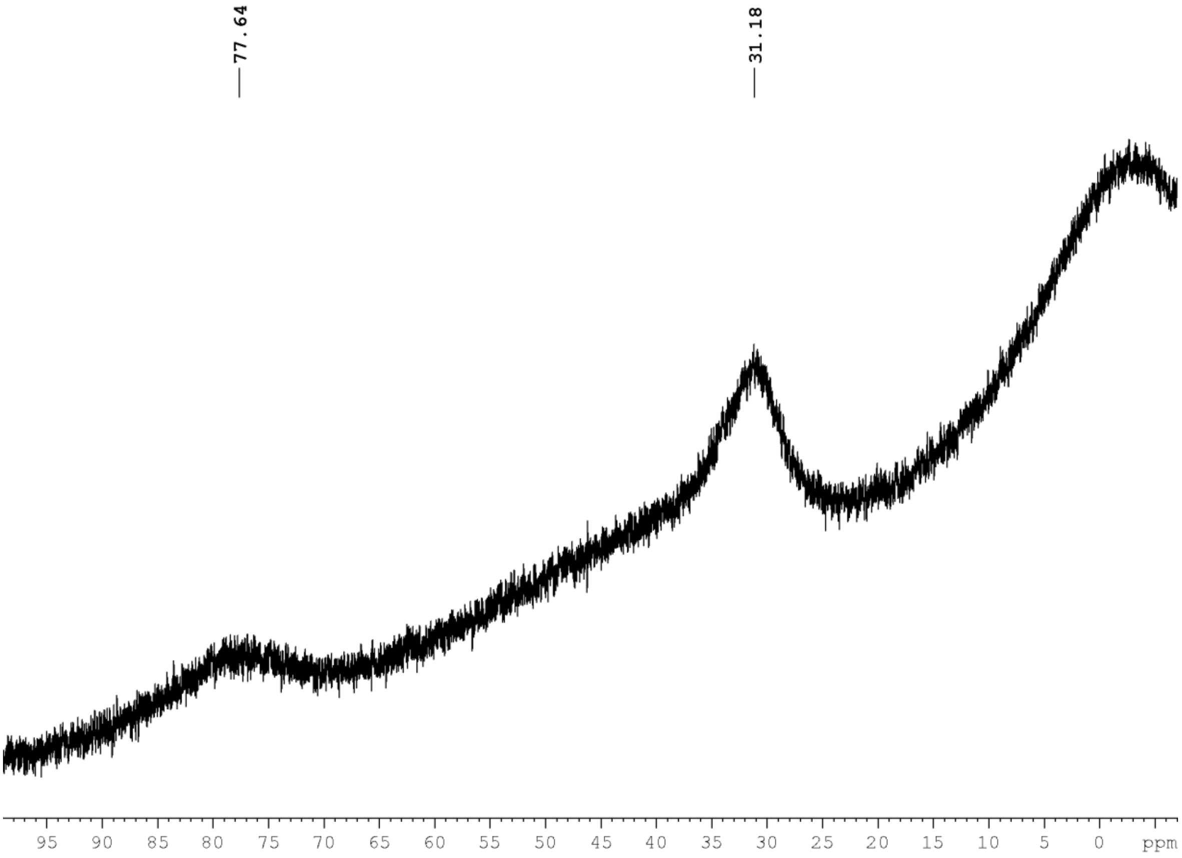

Figure S33. $^{11}B\{^{1}H\}$ NMR spectrum of **BAr^{Bpin}Ar^{Br}Ar^{Me}** in CD_2Cl_2 at 96 MHz.

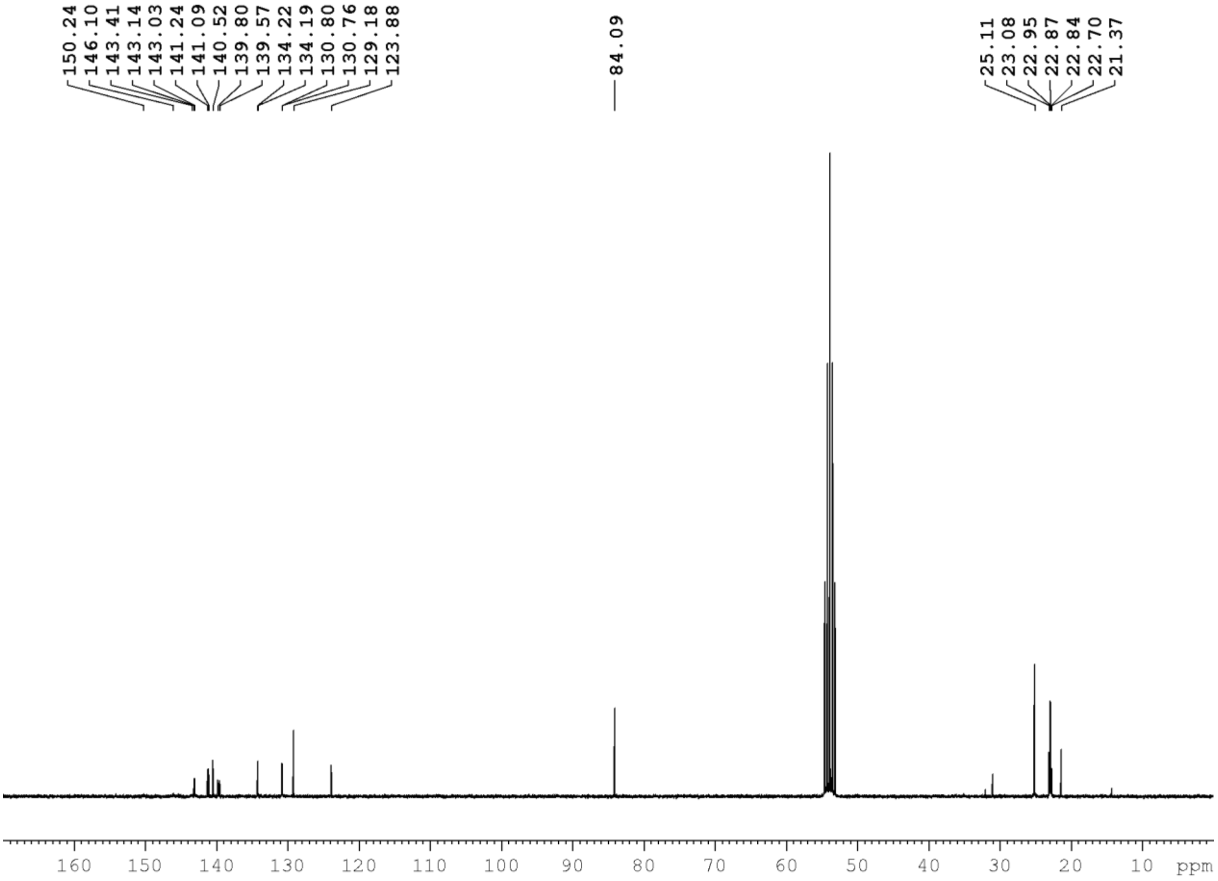

Figure S34. $^{13}C\{^{1}H\}$ NMR spectrum of **BAr^{Bpin}Ar^{Br}Ar^{Me}** in CD_2Cl_2 at 75 MHz.

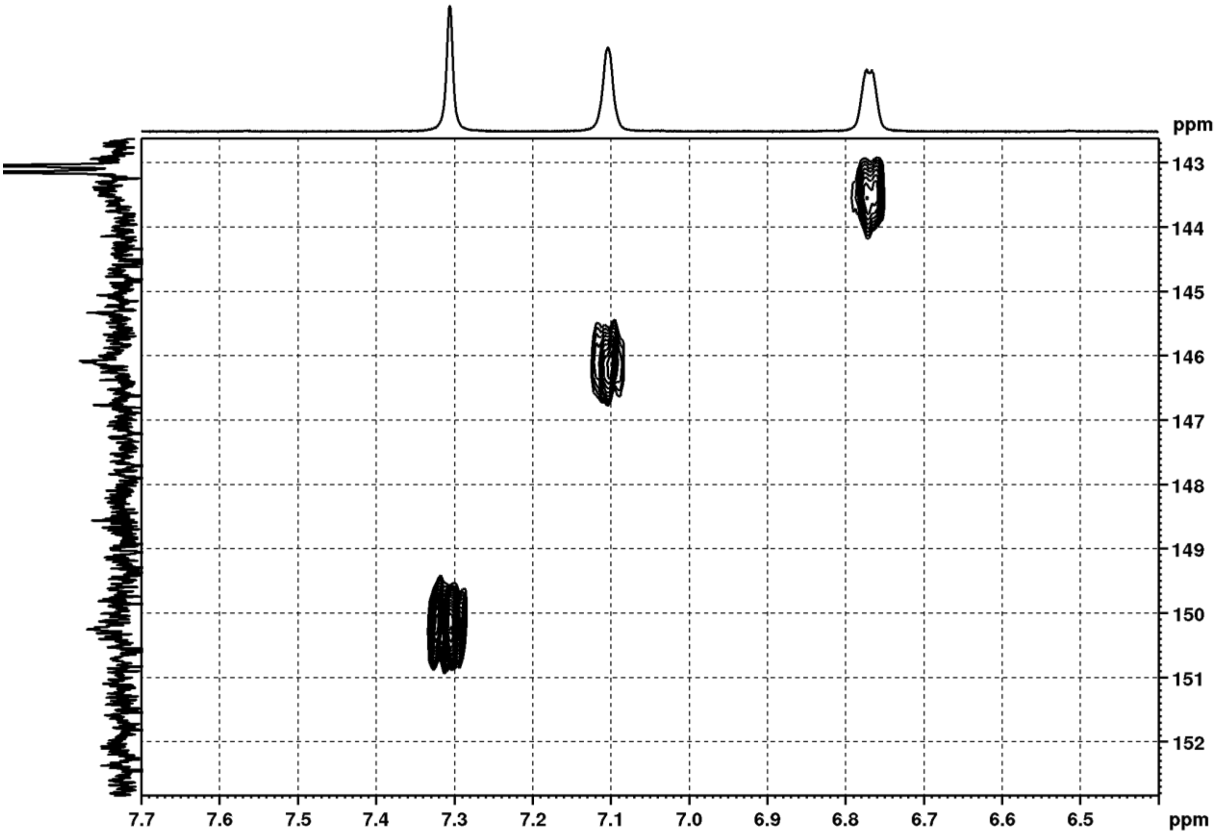

Figure S35. ^{1}H, ^{13}C HMBC NMR spectrum of **BAr^{Bpin}Ar^{Br}Ar^{Me}** in CD_2Cl_2 at 300 MHz.

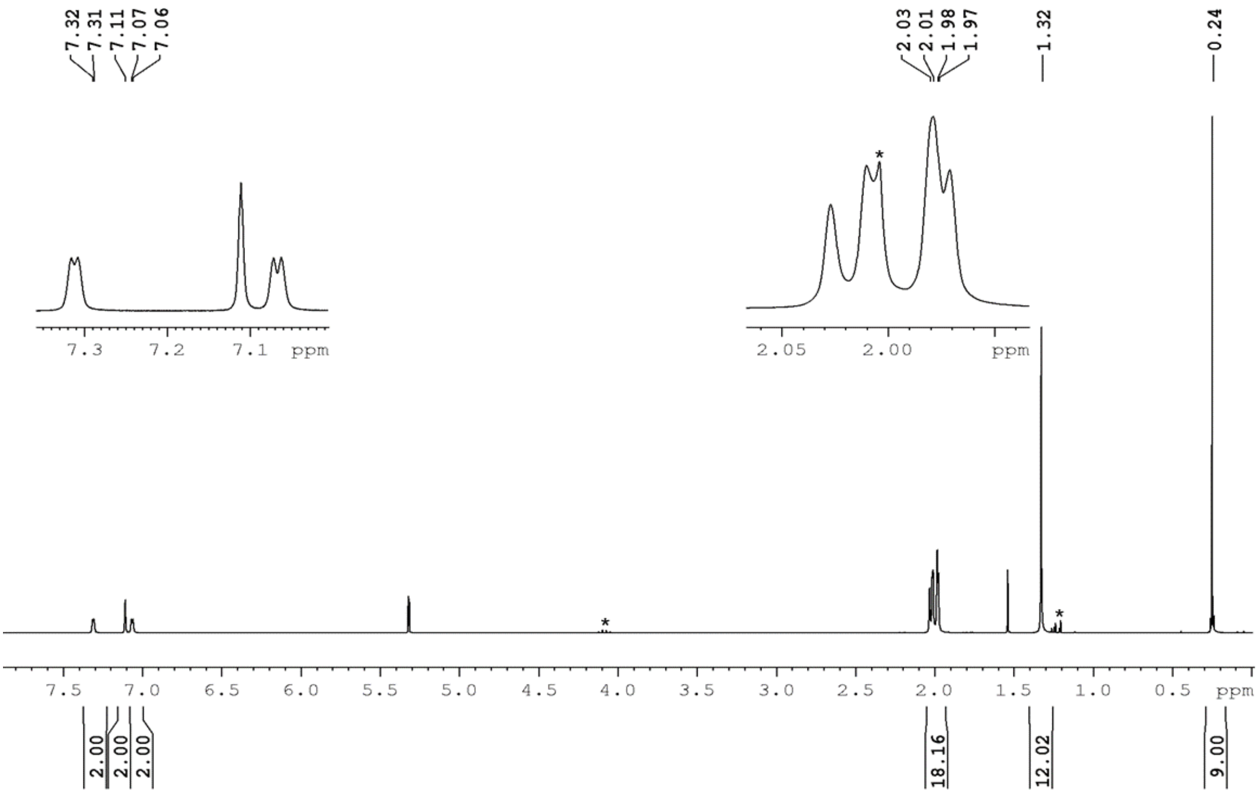

Figure S36. ^{1}H NMR spectrum of **BAr^{Bpin}Ar^{Br}Ar^{SiMe_3}** in CD_2Cl_2 at 300 MHz. Residual solvent peaks (EtOAc) are marked with *.

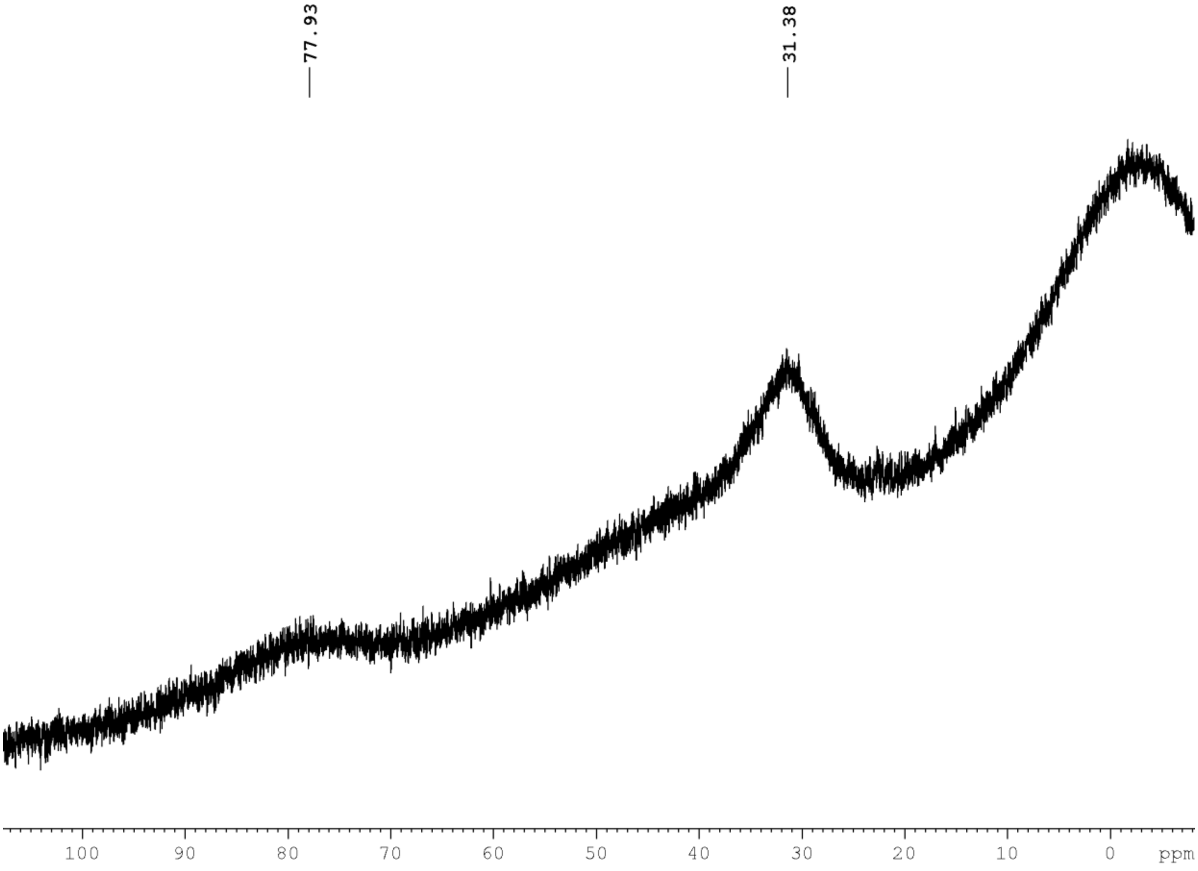

Figure S37. $^{11}B\{^{1}H\}$ NMR spectrum of **BAr^{Bpin}Ar^{Br}Ar^{SiMe_3}** in CD_2Cl_2 at 96 MHz.

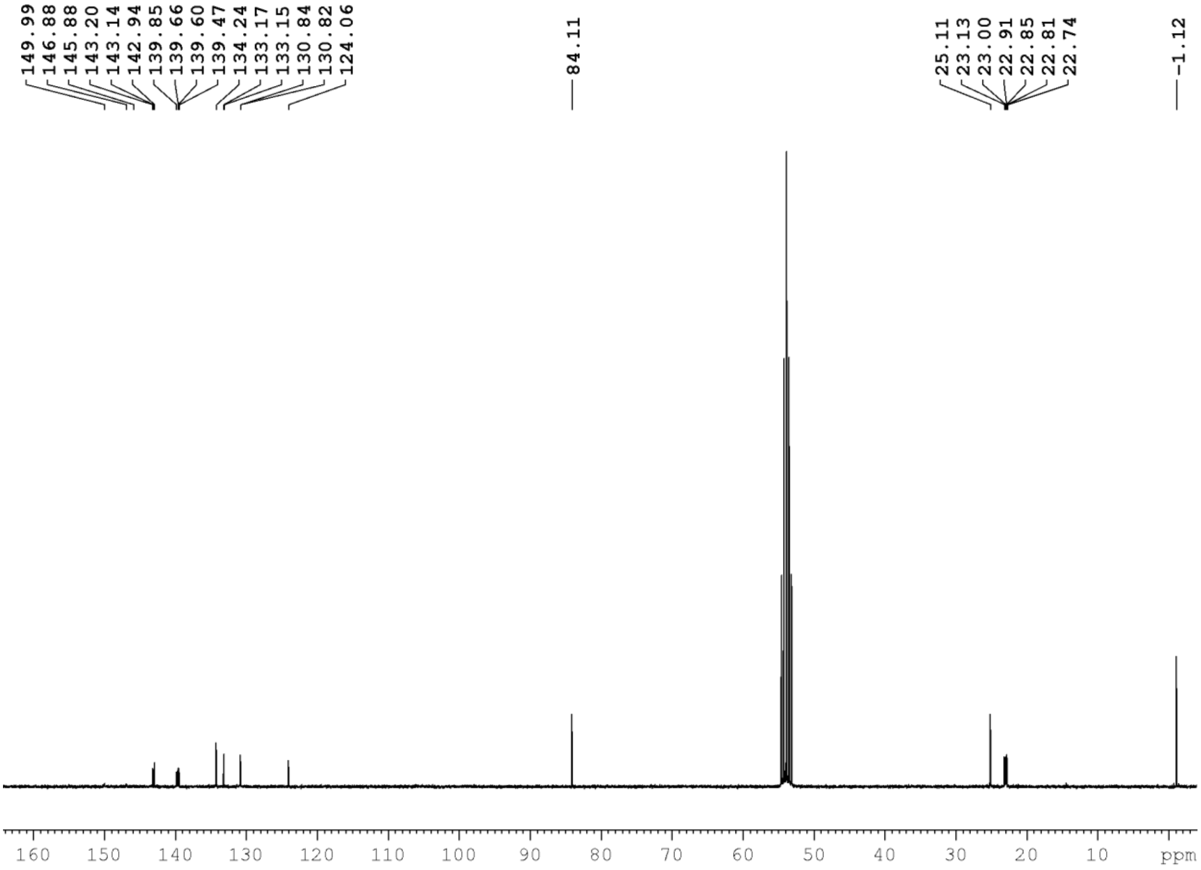

Figure S38. $^{13}C\{^{1}H\}$ NMR spectrum of **BAr^{Bpin}Ar^{Br}Ar^{SiMe_3}** in CD_2Cl_2 at 75 MHz.

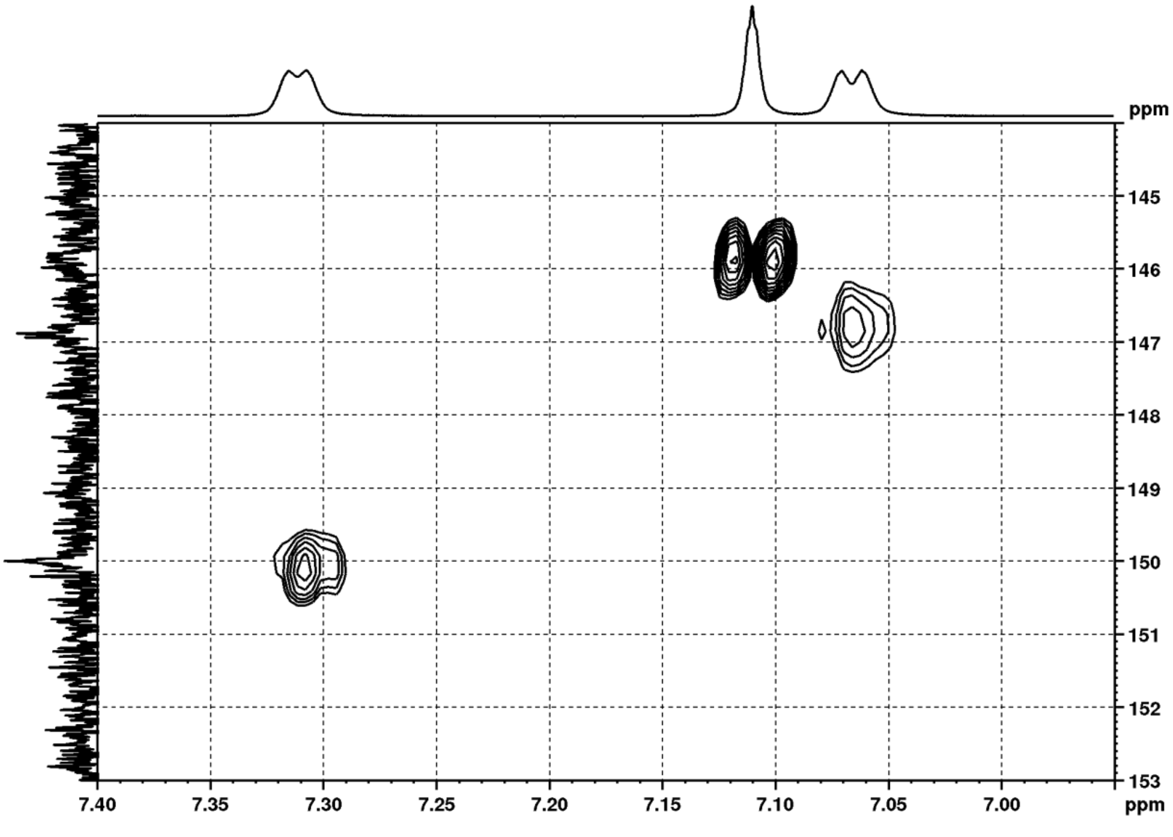

Figure S39. ^{1}H, ^{13}C HMBC NMR spectrum of **BAr^{Bpin}Ar^{Br}Ar^{SiMe_3}** in CD_2Cl_2 at 500 MHz.

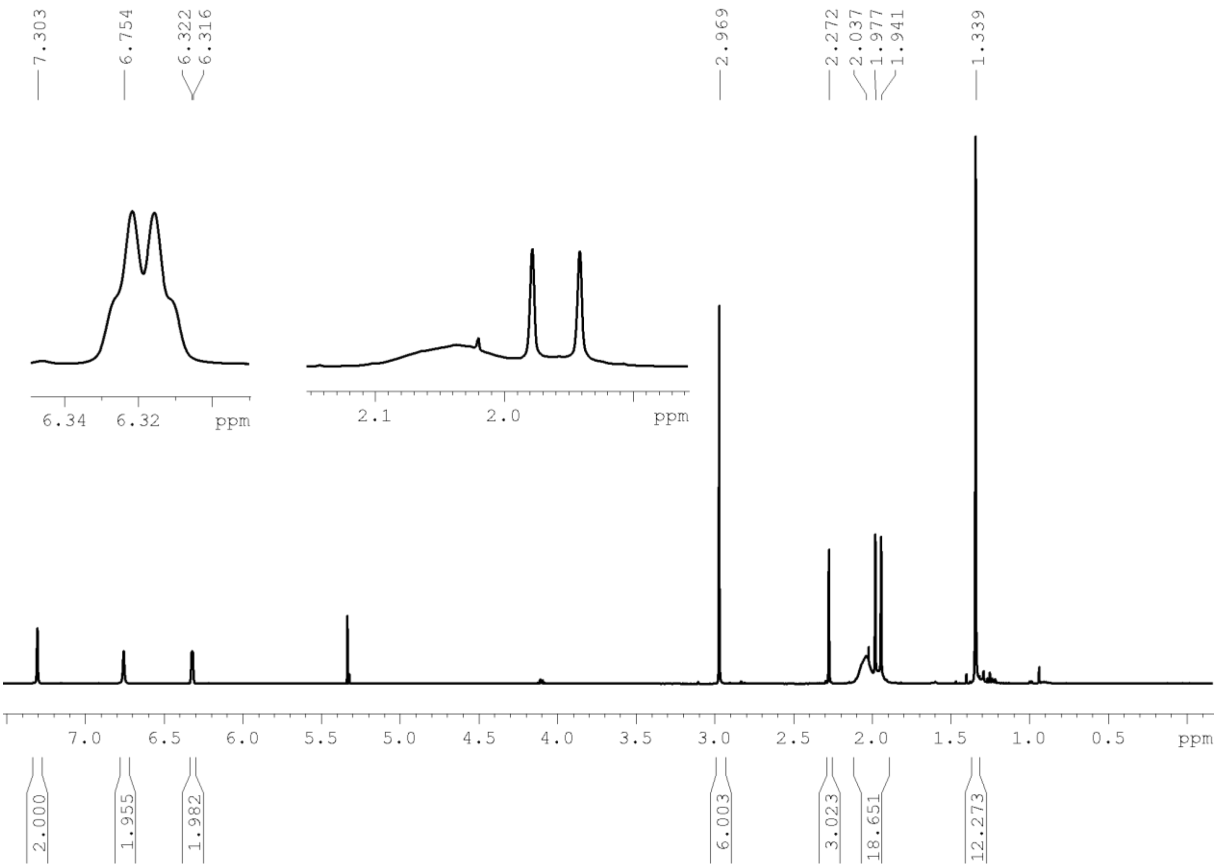

Figure S40. ^{1}H NMR spectrum of **BAr^{Bpin}Ar^{Me}Ar^{NMe_2}** in CD_2Cl_2 at 500 MHz.

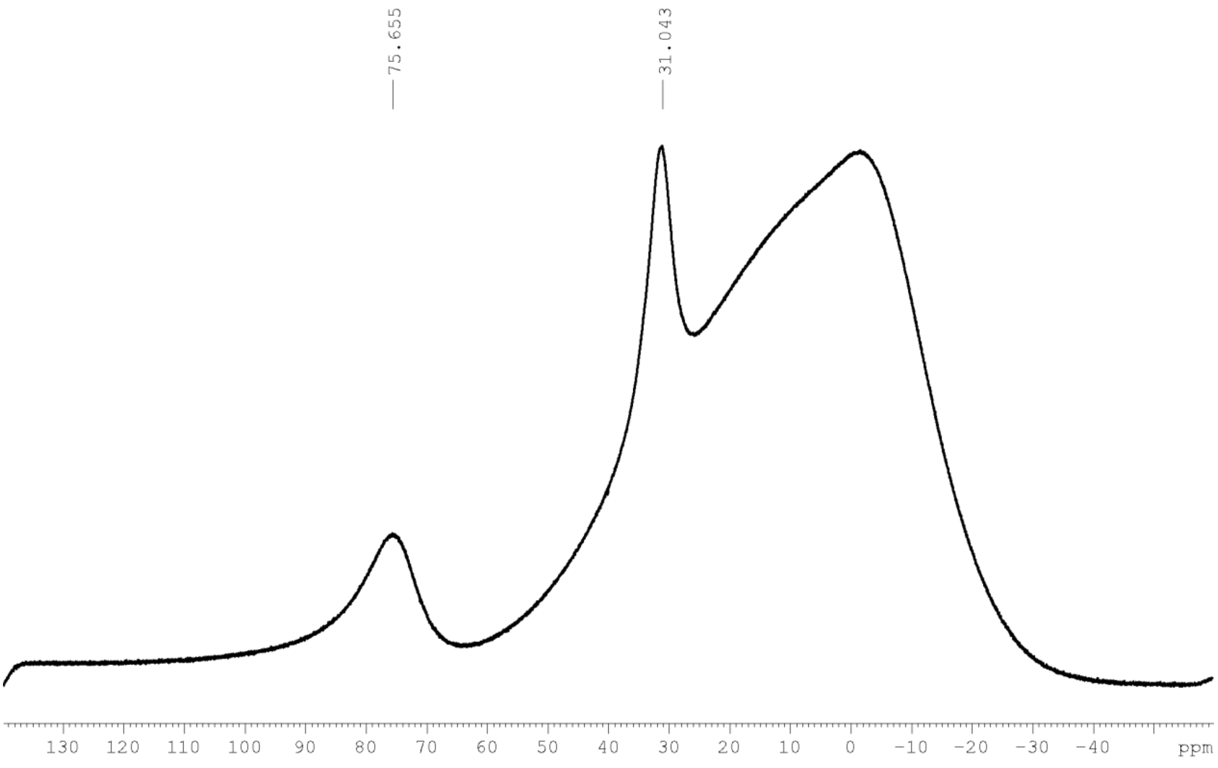

Figure S41. $^{11}B\{^{1}H\}$ NMR spectrum of **BAr^{Bpin}Ar^{Me}Ar^{NMe_2}** in CD_2Cl_2 at 160 MHz.

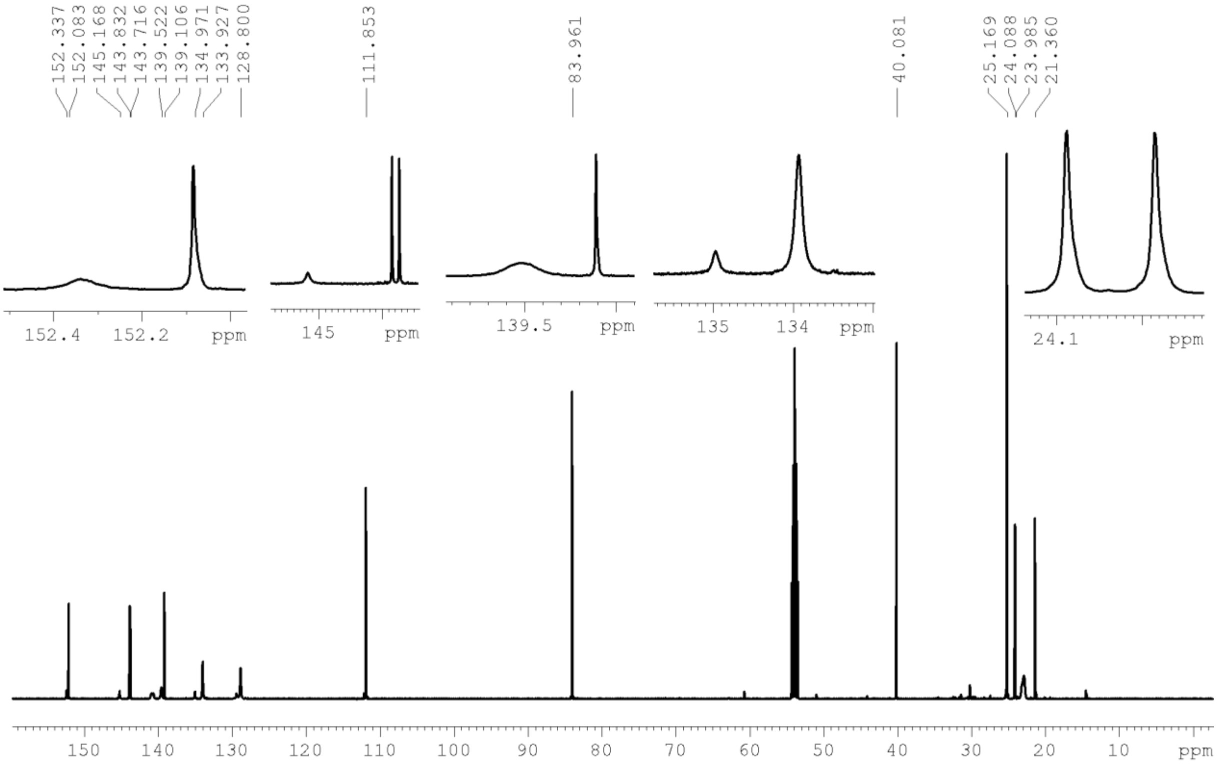

Figure S42. $^{13}C\{^{1}H\}$ NMR spectrum of **BAr^{Bpin}Ar^{Me}Ar^{NMe_2}** in CD_2Cl_2 at 125 MHz.

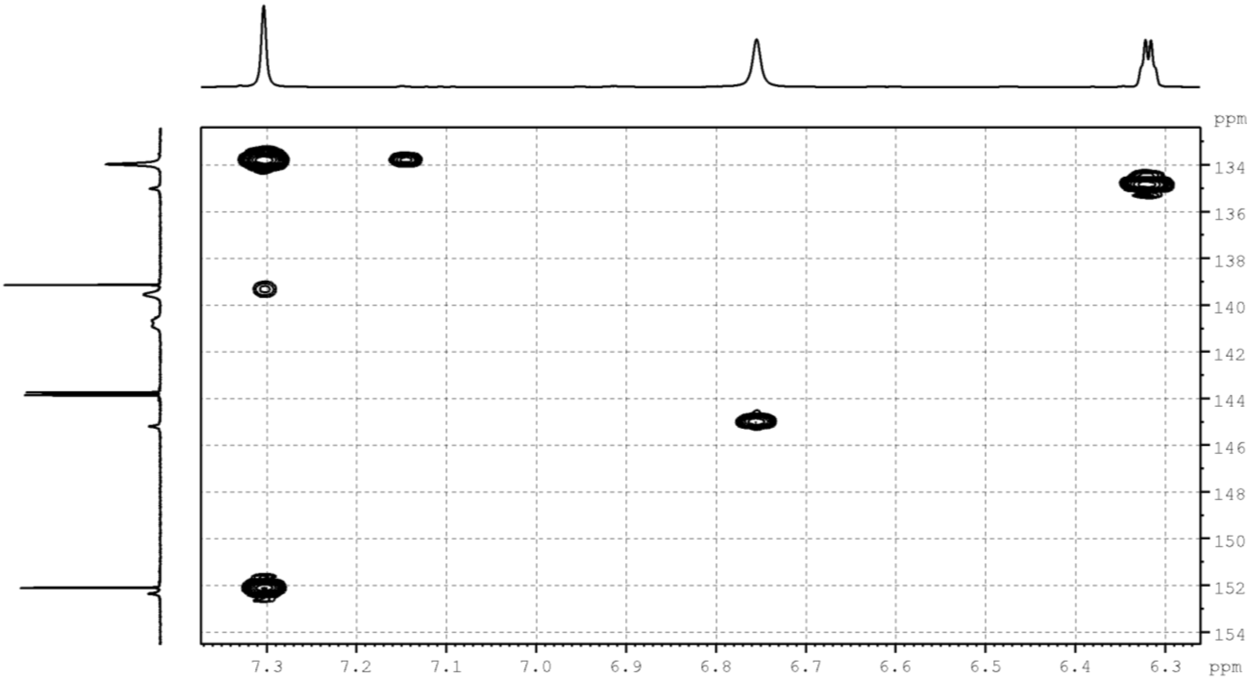

Figure S43. ^{1}H, ^{13}C HMBC NMR spectrum of **BAr^{Bpin}Ar^{Me}Ar^{NMe_2}** in CD_2Cl_2 at 500 MHz.

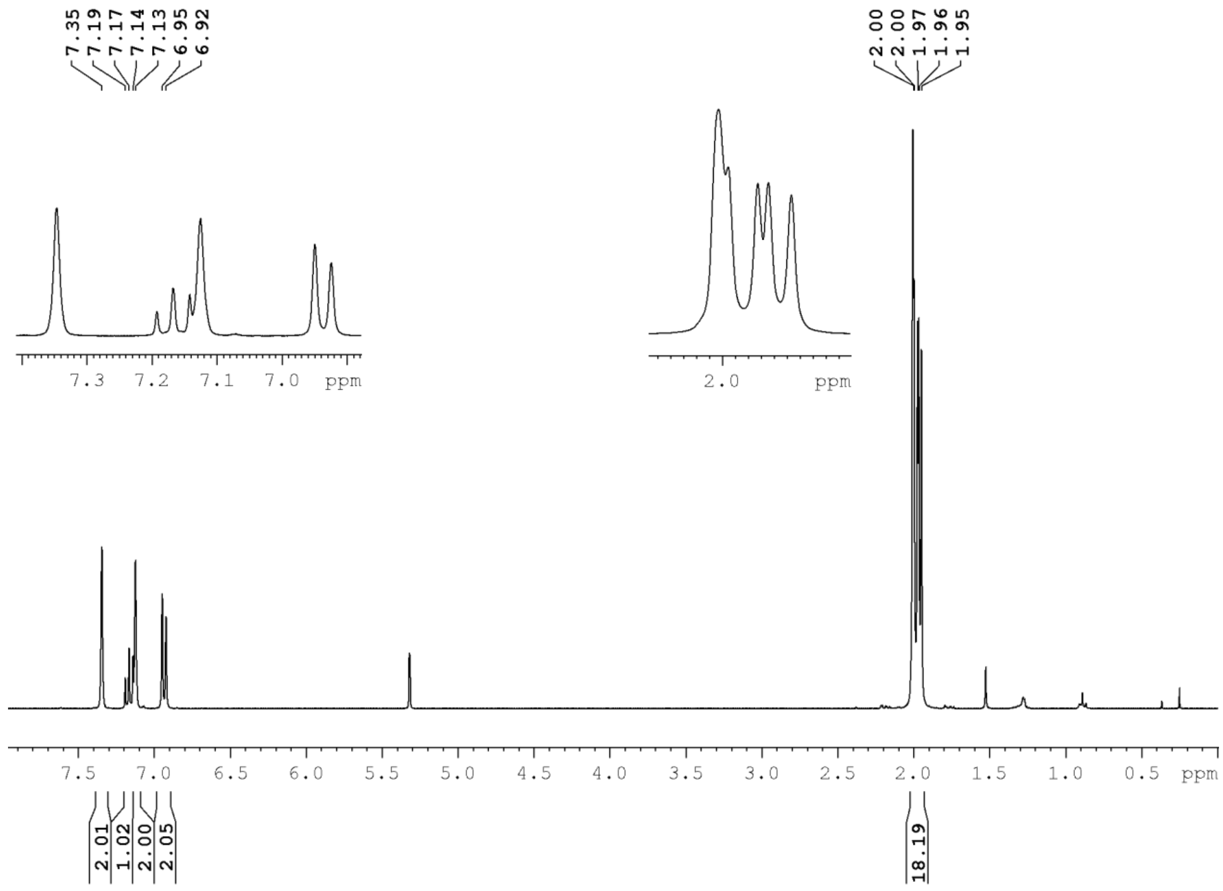

Figure S 44: ^{1}H NMR spectrum of **BAr^{H}Ar^{Br}Ar^{I}** in CD_2Cl_2 at 300 MHz.

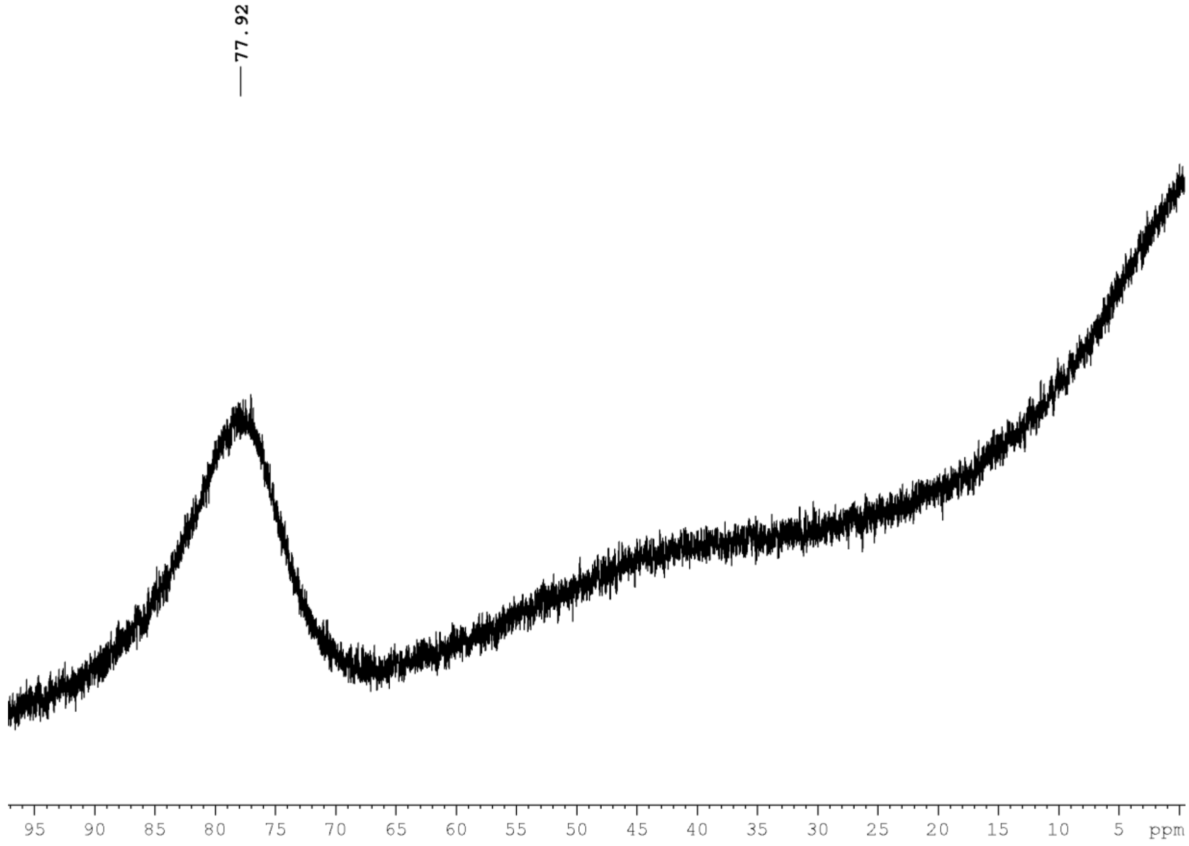

Figure S 45: ^{11}B{^{1}H} NMR spectrum of **BAr^{H}Ar^{Br}Ar^{I}** in CD_2Cl_2 at 96 MHz.

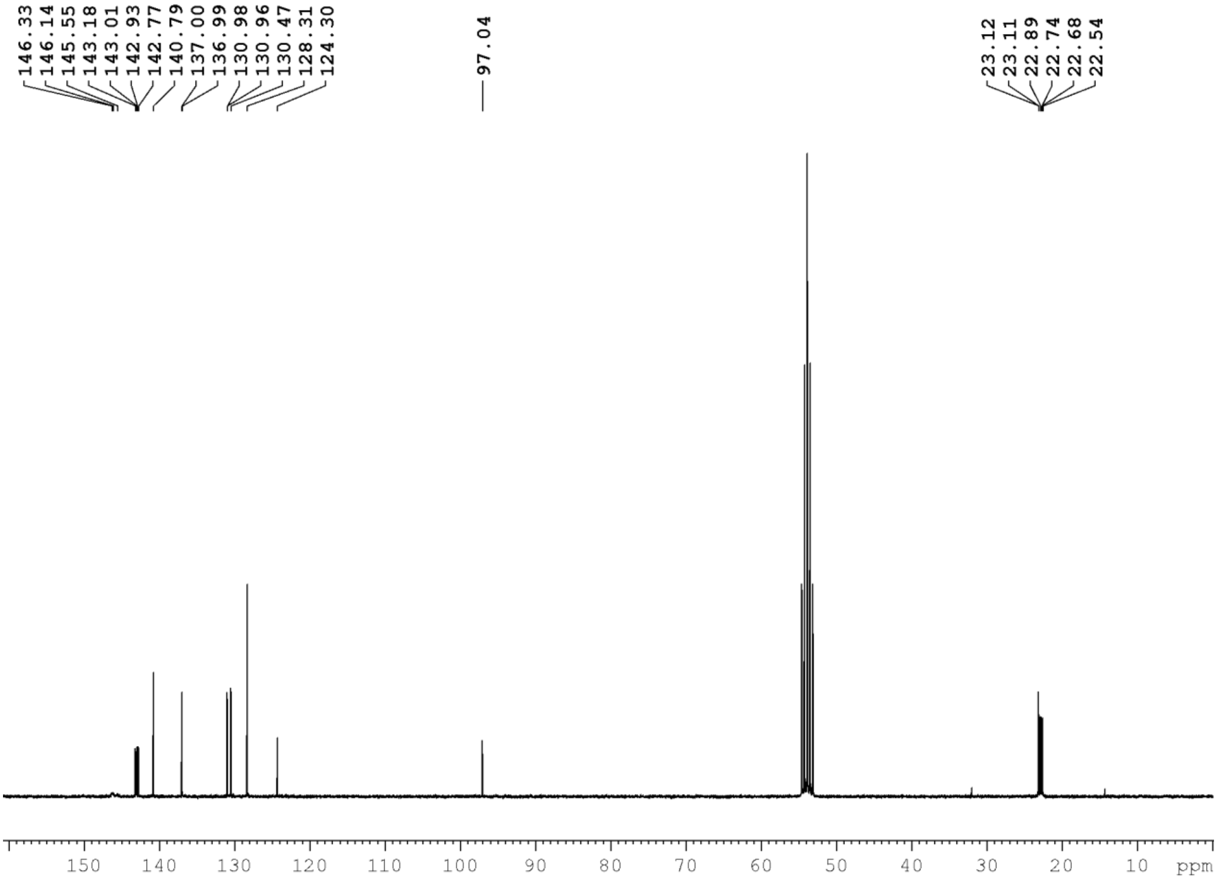

Figure S 46: $^{13}C\{^{1}H\}$ NMR spectrum of **BAr^{H}Ar^{Br}Ar^{I}** in CD_2Cl_2 at 75 MHz.

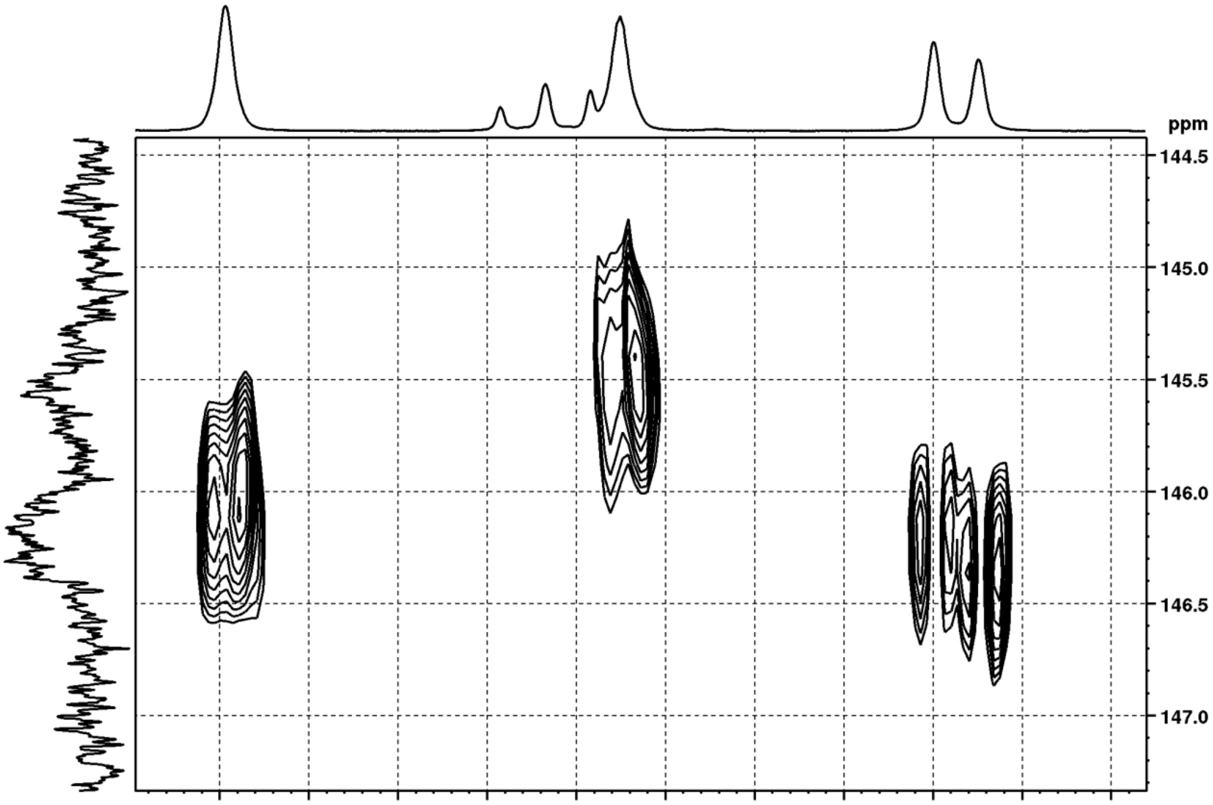

Figure S 47: ^{1}H, ^{13}C HMBC NMR spectrum of **BAr^{H}Ar^{Br}Ar^{I}** in CD_2Cl_2 at 300 MHz.

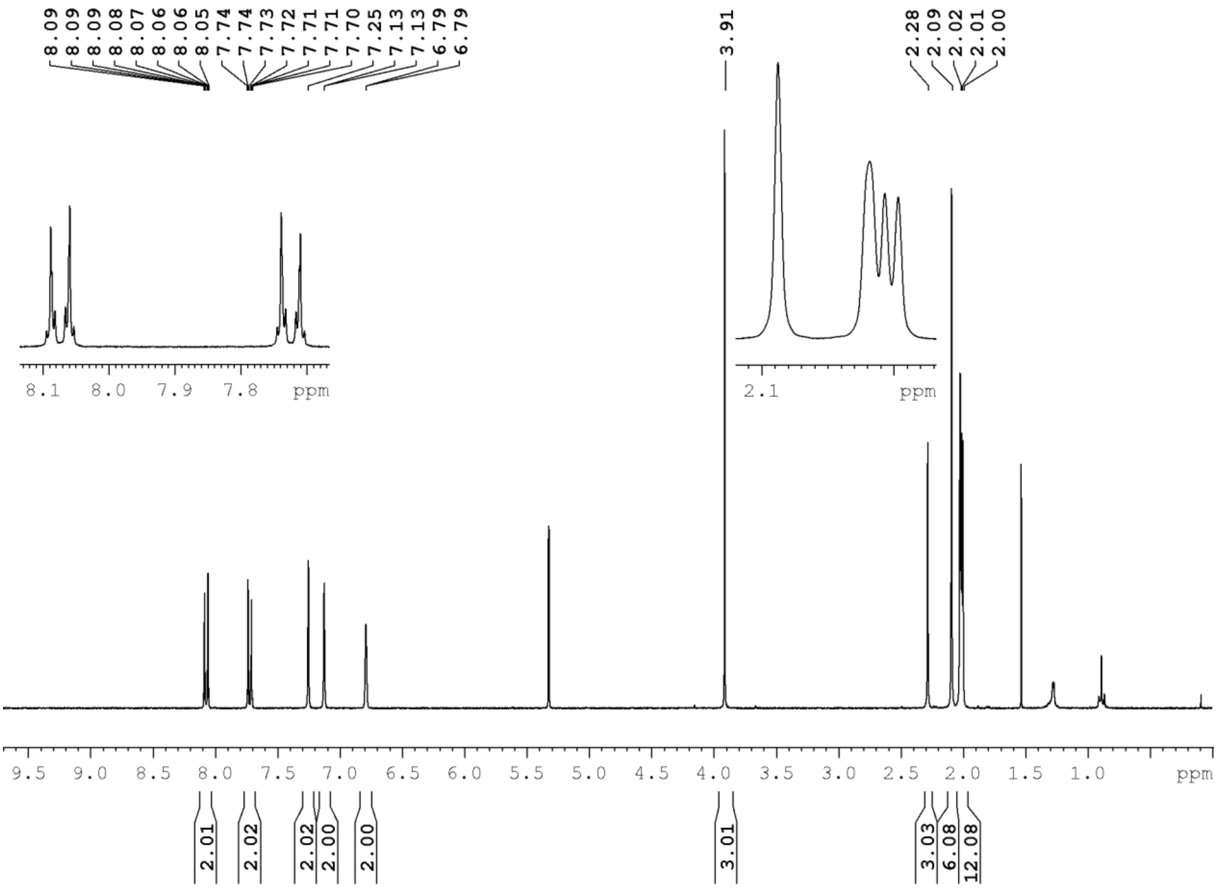

Figure S 48: ^{1}H NMR spectrum of **BAr^{Ph}Ar^{Br}Ar^{I}** in CD_2Cl_2 at 300 MHz.

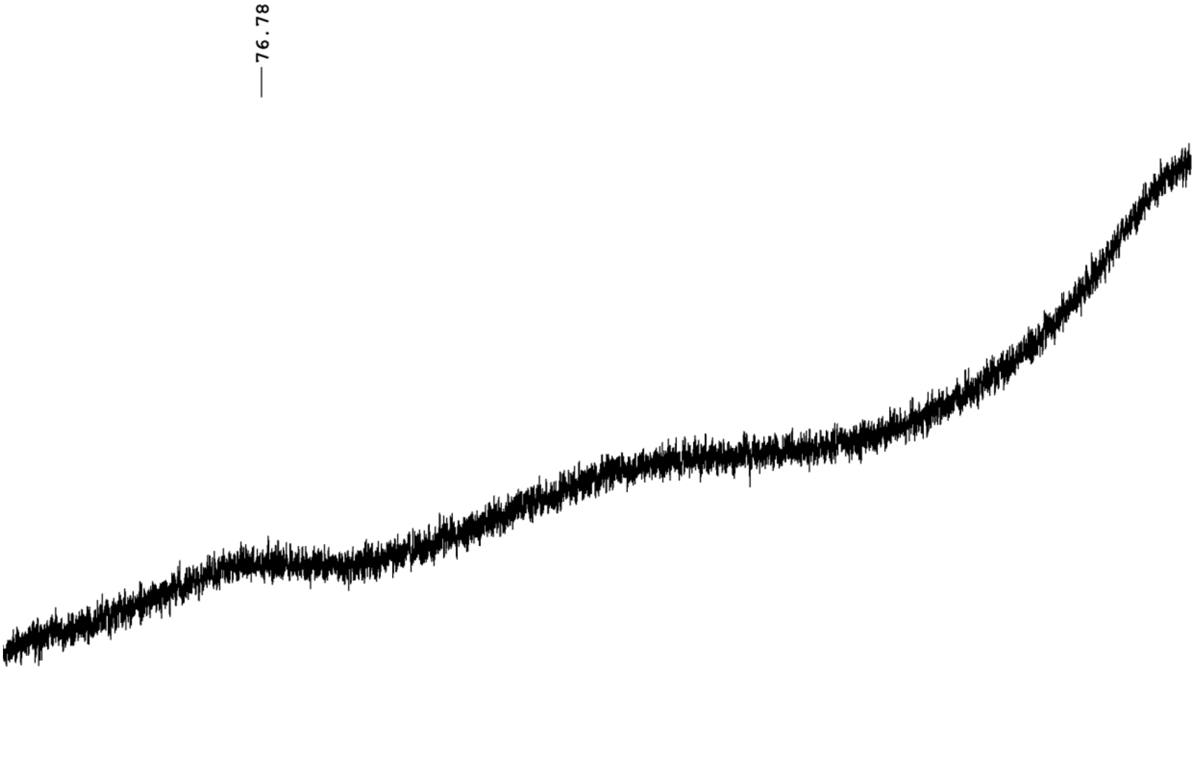

Figure S 49: $^{11}B\{^{1}H\}$ NMR spectrum of **BAr^{Ph}Ar^{Br}Ar^{I}** in CD_2Cl_2 at 96 MHz.

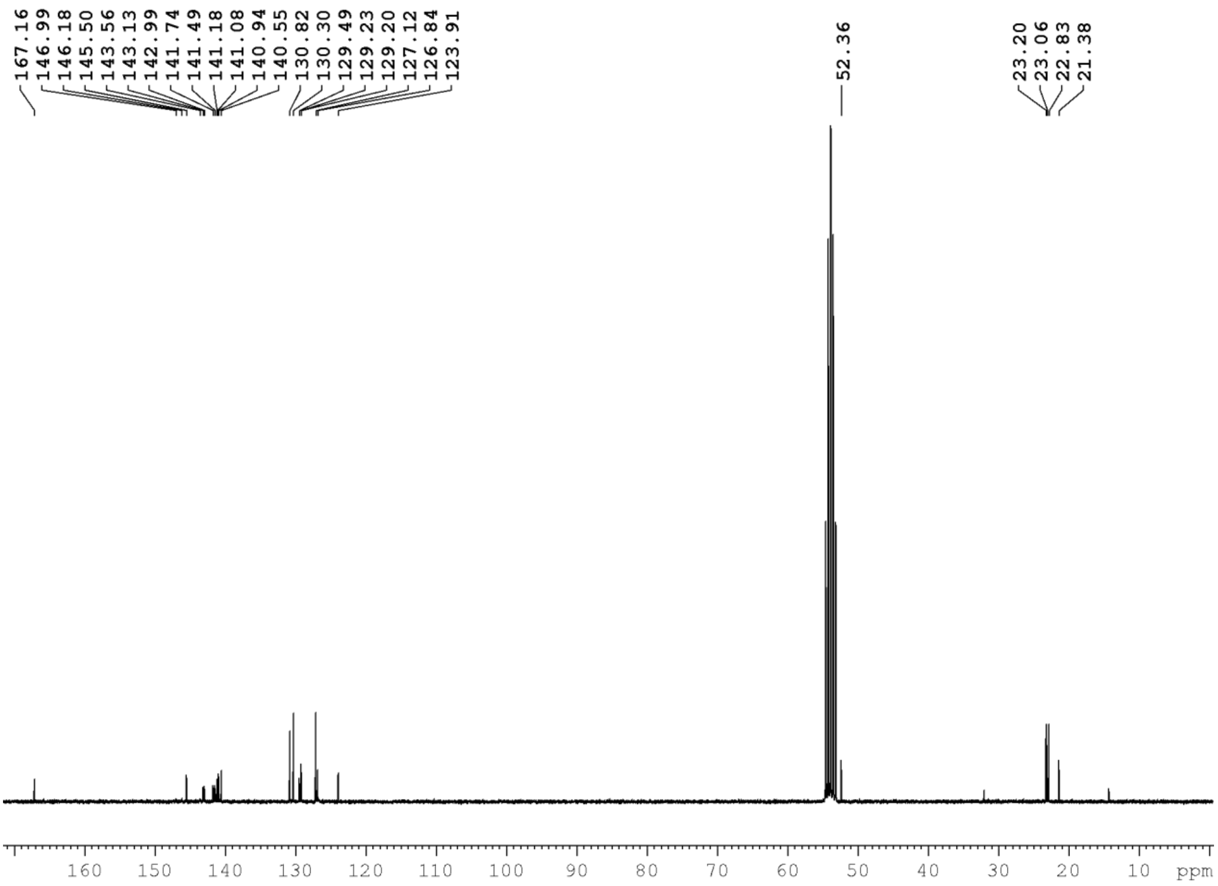

Figure S 50: $^{13}C\{^{1}H\}$ NMR spectrum of $\mathbf{BAr^{Ph}Ar^{Br}Ar^{I}}$ in CD_2Cl_2 at 75 MHz.

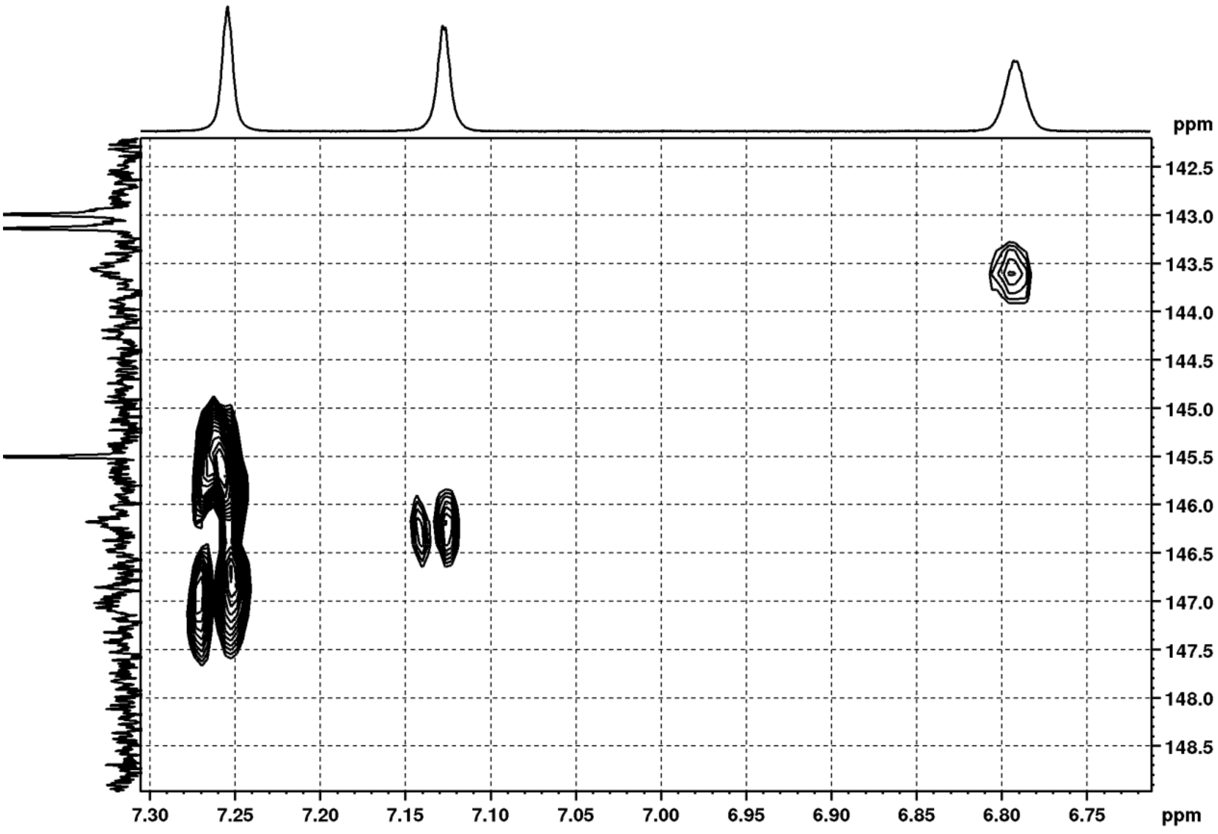

Figure S 51: ^{1}H, ^{13}C HMBC NMR spectrum of $\mathbf{BAr^{Ph}Ar^{Br}Ar^{I}}$ in CD_2Cl_2 at 300 MHz.

References

[1] R. Uson, L. A. Oro, J. A. Cabeza, H. E. B. Bryndza, M. P. Stepro, *Inorg. Synth.* **1985**, *23*, 126-130.

[2] E. Zysman-Colman, K. Arias, J. S. Siegel, *Can. J. Chem.* **2009**, *87*, 440-447.

[3] C.-W. Chiu, Y. Kim, F. P. Gabbai, *J. Am. Chem. Soc.* **2009**, *131*, 60-61.

[4] S. S. Zalesskiy, V. P. Ananikov, *Organometallics* **2012**, *31*, 2302-2309.

[5] G. Sheldrick, *Acta Crystalogr.* **2015**, *A71*, 3-8.

[6] G. Sheldrick, *Acta Crystalogr.* **2008**, *A64*, 112-122.

[7] A. P. Liesen, A. T. Silva, j. C. Sousa, P. H. Menezes, R. A. Oliveira, *Tetrahedron Lett.* **2012**, *53*, 4240-4242.

[8] S. Darses, G. Michaud, J.-P. Genêt, *Eur. J. Org. Chem.* **1999**, *1999*, 1875-1883.

[9] S. Griesbeck, Z. Zhang, M. Gutmann, T. Lühmann, R. M. Edkins, G. Clermont, A. N. Lazar, M. Haehnel, K. Edkins, A. Eichhorn, M. Blanchard-Desce, L. Meinel, T. B. Marder, *Chem. Eur. J.* **2016**, *22*, 14701-14706.
